# Supplementary material for: Profiling Phospholipids within Atlantic Salmon Salmo salar with Regards to a Novel Terrestrial Omega-3 Oil Source
Source: Metabolites. 2022 Sep 10;12(9):851. doi: 10.3390/metabo12090851 (PMC9503986; doi:10.3390/metabo12090851)
Supplement: Supplementary file 1 [file metabolites-12-00851-s001.zip › Supplementary Figures S1 and S2.pdf]

Brain (Area)				Eye (Area)				Gill (Area)				Intestine (Area)				Liver (Area)			
Int.		Final		Int.		Final		Int.		Final		Int.		Final		Int.		Final	
C	G	C	G	C	G	C	G	C	G	C	G	C	G	C	G	C	G	C	G

Simple ID	Primary ID	Secondary ID	Brain				Eye				Gill				Intestine				Liver			
			Int.		Final		Int.		Final		Int.		Final		Int.		Final		Int.		Final	
			Fold	p	Fold	p	Fold	p	Fold	p	Fold	p	Fold	p	Fold	p	Fold	p	Fold	p	Fold	p
PE 34:2	PE 16:1/18:1				-1.3																	
	PE 16:0/18:2				1.1																	
PE O-36:5	PE O-18:3/18:2								3.6	*												
	PE P-16:0/20:4								2.9	**												
PE O-36:3	PE O-18:2/18:1								-1.3													
	PE P-18:0/18:2								1.2													
PE 36:6	PE 16:1/20:5																N/A	*				
	PE 14:0/22:6																N/A	*				
PE 36:4	PE 18:1/18:3				2.3	**																
	PE 16:0/20:4				1.9	**																
PE 36:3	PE 18:1/18:2														-2.6	*	-3.0	*				
	PE 16:0/20:3														1.2		-1.1					
PE 36:2	PE 18:1/18:1				-1.1		-3.5	**	-1.3						-5.3	**	-13.7	*			-8.7	**
	PE 18:0/18:2 (a)	PE 16:0/20:2			1.0		-1.2	*	1.5						1.2		-7.0	**			-1.1	
	PE 18:0/18:2 (b)																1.3					
PE O-38:7	PE O-18:3/20:4								7.0	**												
	PE O-18:2/20:5				1.1				-1.3				-3.1									
	PE P-16:0/22:6				2.0	**			1.1				-1.3									
PE O-38:6	PE P-18:1/20:4						1.3	*														
	PE P-18:0/20:5						-1.3															
PC 30:1	PC 14:0/16:1 (a)										-5.1	*	-4.3	**								
	PC 14:0/16:1 (b)										-4.8	*	N/A	*								
	PC 14:1/16:0										-4.9	**										
PE O-38:5	PE O-18:2/20:3								1.5													
	PE P-18:0/20:4 (a)		1.6																			
	PE P-18:0/20:4 (b)		1.4						3.1	**												
PE 38:7	PE 18:2/20:5	PE 18:3/20:4	1.2		1.9	**			1.6	*							-1.5					
	PE 16:1/22:6		-1.3		-1.1				-1.1								-7.0	*				
PE 38:6	PE 18:2/20:4						3.5	**	3.2	**					1.1		2.1	**	7.7	**	3.8	**
	PE 18:1/20:5	PE 18:2/20:4					-4.4	**	-1.5				-5.1	**	-3.0		-5.9	**	-5.6	**	-2.7	*
	PE 16:0/22:6						-1.6	*	1.2				-1.5		-1.2		-1.2		-1.6		-1.3	
PE 38:5	PE 16:0/22:5	PE 18:1/20:4					1.0						-1.7	**			-1.2		1.7	*	1.4	
	PE 18:0/20:5						-1.6	*					-1.9				-1.4		-1.1		-1.3	
PG 36:6	PG 16:1/20:5																N/A	**				
	PG 14:0/22:6																N/A	**				
PE 38:4	PE 18:1/20:3		1.7	*	2.1	**									-2.0						-2.4	
	PE 16:0/22:4														3.9		2.2	*				
	PE 18:0/20:4		1.7		2.5	*									4.2	*	2.1	**			4.1	**
	PE 18:3/22:5																				3.0	*
PE 38:3	PE 18:2/20:1														-1.8							
	PE 18:0/20:3														1.2							
	PE 18:1/20:2				1.4	*																
	PE 16:1/22:2				1.1																	
PE O-40:7	PE P-18:1/22:5				1.1				-1.2				-2.9	*								
	PE P-18:0/22:6				1.3				1.2				-1.2									
PC 32:2	PC 14:0/18:2														1.1							
	PC 16:1/16:1														-3.9	*						
PC 32:1	PC 16:0/16:1 (a)	PC 14:0/18:1					-2.2	**	-1.8	**	-2.9	**	-2.7	**								
	PC 16:0/16:1 (b)						-1.8	*	1.0		-1.8	**	1.0									
	PC 16:0/16:1 (c)	PC 14:0/18:1					1.1		2.0	**	2.3		1.6	**								
	PC 16:0/16:1 (d)										-7.1	**										
PE 40:9	PE 20:4/20:5		2.1		1.5				1.1										-1.0			
	PE 18:3/22:6		1.5		3.6	**			2.8	**									1.1			
PE 40:8	PE 20:3/20:5	PE 20:4/20:4	1.9	*	3.1	**			3.1	*											1.9	
	PE 18:2/22:6		1.1		1.4	**			1.7	**											1.1	
PE 40:7	PE 18:2/22:5																1.1		1.5		1.1	
	PE 18:3/22:4														2.5							
	PE 20:2/20:5		1.6	*	1.7	**			1.4						-1.3		-1.3		1.3		1.1	
	PE 18:1/22:6 (a)		-1.7		-1.1				-1.2						-2.8	**	-3.1	*	-4.3	**	-2.9	
	PE 18:1/22:6 (b)		-1.7												2.2							
PE 40:6	PE 18:1/22:5 (a)																-2.6	*			-2.6	*
	PE 20:1/20:5																		-2.5	**	-1.4	
	PE 18:1/22:5 (b)																				-5.2	*
	PE 18:0/22:6																1.1		1.0		1.2	
PC 33:1	PC 16:0/17:1 (a)	PC 15:0/18:1									-2.6	**										
	PC 16:0/17:1 (b)										-2.1											
PE 40:5	PE 18:0/22:5 (a)	PE 20:1/20:4													2.3				2.9	**	1.8	*
	PE 20:4/20:1	PE 18:1/22:4							1.0													
	PE 20:0/20:5														1.9				2.0	*	2.2	*
	PE 18:0/22:5 (b)								1.7	*					1.3						-2.0	
PG 38:6	PG 18:1/20:5																-3.6	**			-8.3	**
	PG 16:0/22:6																1.1				-3.4	**
PE 40:4	PE 20:1/20:3																		-2.9	**	N/A	*
	PE 18:1/22:3	PE 20:1/20:3	1.5												6.5	*						
	PE 18:0/22:4		1.4				1.6	**							4.8	*	2.8	**	6.7	**	N/A	**
	PE 20:0/20:4						2.1								10.4	*	3.9	**	13.3	*	N/A	**
PC 34:4	PC 16:1/18:3																-2.2					
	PC 16:0/18:4																-1.0					
PC 34:2	PC 16:1/18:1				-1.4	*					-6.3	**					-7.7	**				
	PC 16:0/18:2 (a)				1.4	**					-1.4	*			-1.5		-1.1					
	PC 16:0/18:2 (b)	PC 14:0/20:2			1.3						3.2	**			4.4	*						
PE 42:10	PE 20:5/22:5		-1.1		-1.1																	
	PE 20:4/22:6 (a)		1.4		2.7	**	1.2		2.7	**					1.7							
	PE 20:4/22:6 (b)		1.7	*	2.0	**	1.4		2.6	**					4.7							
PE 42:8	PE 20:2/22:6 (a)		-1.1						1.5										-1.1			
	PE 20:2/22:6 (b)		-1.3						1.5										2.8	*		
PE 42:6	PE 20:1/22:5						-1.6	*	1.4						-1.1		-1.0		1.1		-1.2	
	PE 20:0/22:6						-1.4	*	1.1						2.8		2.0	*	2.0	*	2.1	**
PE 42:5	PE 20:1/22:4														2.0		2.0					
	PE 20:0/22:5														4.3		3.1	**				
PC 36:6	PC 18:3/18:3								4.8	**							4.3	*	1.6		6.7	*
	PC 16:1/20:5 (a)						-3.2	**														
	PC 16:1/20:5 (b)	PC 18:2/18:4	-2.1	*	-2.1		-2.2		-4.4	*	-10.1	**	-4.1	*	-2.4		-7.2	*	-3.3	**	-3.4	
	PC 14:0/22:6		-2.1		-2.0	*	-3.1	**	-3.0	*	-6.2	**	-3.3	*	-2.5	*	-4.8	*	-3.8	**	-3.6	
PC 36:5	PC 18:2/18:3														1.1		2.0					
	PC 16:0/20:5														1.2		-1.3					
PC 36:4	PC 18:2/18:2	PC 16:1/20:3					-1.4	*	1.3	*	-1.3		-1.3		-1.5		-1.0		-1.6	**	1.0	
	PC 18:1/18:3				1.6	*	-1.8	*	1.3		-1.9	*	1.4		-2.2		-1.5		-2.6	*	-1.0	
	PC 16:0/20:4 (a)				2.1	**	1.4		1.4	*	2.0	*	1.1		1.3		1.0		-1.2		1.0	
	PC 16:0/20:4 (c)																		-1.4			
	PC 16:0/20:4 (b)						2.9	**	2.9	**			1.7	**	4.7	*	3.3	**	5.3	**	3.7	**

Simple ID	Primary ID	Secondary ID	Brain				Eye				Gill				Intestine				Liver			
			Int.		Final		Int.		Final		Int.		Final		Int.		Final		Int.		Final	
			Fold	p	Fold	p	Fold	p	Fold	p	Fold	p	Fold	p	Fold	p	Fold	p	Fold	p	Fold	p
PC 36:3	PC 18:1/18:2								-1.8	**	-2.6	**			-3.2	**	-2.9	**	-3.4	**	-2.0	**
	PC 16:0/20:3 (a)	PC 18:0/18:3 | 18:1/18:2							3.7		-1.1				1.2		1.2		1.0		1.7	
	PC 16:0/20:3 (b)										11.4	**			1.5		2.5		15.9	**		
	PC 16:0/20:3 (c)								1.5	*												
	PC 18:0/18:3								3.1	**												
PC 36:2	PC 18:1/18:1						-3.7	**	-1.6	**	-5.6	**	-3.0	**	-6.9	**	-6.1	**	-6.5	**	-3.9	**
	PC 16:0/20:2 (a)						-1.4		1.4	*	-1.7	*	1.1		-1.1		-1.4	*	-3.0	**	-1.2	
	PC 16:0/20:2 (b)	PC 18:0/18:2					1.6	*	1.9	*	-1.1		1.3	*							1.1	
	PC 18:0/18:2														1.2		1.1		-1.3			
PC O-38:6	PC P-18:0/20:5								-2.2	**			-2.8	*								
	PC O-16:0/22:6								-1.2				-1.6	**								
PC 38:8	PC 18:3/20:5																1.5					
	PC 16:2/22:6																-1.1					
PC 38:7	PC 18:3/20:4																				5.8	*
	PC 18:2/20:5																		1.7	*	1.7	
	PC 16:1/22:6 (a)																		-3.9	**	-2.1	
	PC 16:1/22:6 (b)																		-1.8	*	1.2	
PC 38:6	PC 18:3/20:3																2.0	*				
	PC 18:2/20:4										N/A	**			9.0	*	4.2		9.6	**		
	PC 18:1/20:5										-4.3	**	-3.1	*	-1.6		-3.4	*	-2.0	*		
	PC 16:0/22:6										-1.6	*	-1.1		-1.1		1.0		-1.7	**		
PC 38:5	PC 18:1/20:4 (a)																		-1.4	*		
	PC 18:1/20:4 (b)	PC 16:0/22:5																	1.4			
	PC 16:0/22:5 (a)	PC 18:1/20:4					1.1								1.3							
	PC 18:0/20:5						1.1								2.0				1.5			
	PC 16:0/22:5 (b)														-1.0							
PC 38:4	PC 18:2/20:2														-1.9				-2.1	**	-1.2	
	PC 18:1/20:3												-1.9	*			1.1					
	PC 18:3/20:1	PC 18:1/20:3													-1.1				1.1		1.4	
	PC 16:0/22:4								2.4	**	3.5	*	1.7	*			2.1	**	2.7	**		
	PC 18:0/20:4 (a)	PC 16:0/22:4					3.6	**	2.1	*	1.4		1.7	**	3.8	*	2.6	*	4.5	**	2.0	**
	PC 18:0/20:4 (b)						2.2	**													3.9	**
PC 38:3	PC 18:2/20:1	PC 18:1/20:2													-2.0	*			-3.1	**		
	PC 18:0/20:3														1.4				1.6			
PC 38:2	PC 18:1/20:1																		-3.6	**		
	PC 18:0/20:2																		1.3			
PG 44:11	PG 22:5/22:6 (a)																		1.7			
	PG 22:5/22:6 (b)																		6.6			
PC 40:9	PC 20:4/20:5 (a)						1.7		1.5						2.1		-1.2		3.7	**	2.2	
	PC 18:3/22:6						1.2		3.3	**					1.4		2.1	*	1.2		3.6	**
	PC 20:4/20:5 (b)																		8.6	**		
PC 40:8	PC 18:3/22:5						2.9	**	3.4	**					2.1		2.0	*	2.9	**	4.6	**
	PC 20:3/20:5	PC 20:4/20:4	2.1	*			2.5	**	2.9	**					3.0		2.1		5.3	**	4.6	**
	PC 18:2/22:6 (a)		-1.1				-1.2		1.6						1.1		1.1		-1.2		1.5	
	PC 18:2/22:6 (b)																7.8	**				
PC 40:7	PC 18:2/22:5						1.8	*	2.1	*					1.8		1.3		1.7	*		
	PC 20:2/20:5	C20:3/20:4					1.9	*	1.7						1.8		1.5		2.4	*		
	PC 18:1/22:6 (a)		-1.4				-2.3	**	1.1		-3.2	**	-1.6		-2.6	*	-2.5	*	-3.7	**		
	PC 18:1/22:6 (b)		-2.3	**			-4.2	**	-1.7		-4.8	**	-3.2	*								
PC 40:6	PC 18:1/22:5																-3.1	**				
	PC 20:1/20:5																1.4				1.2	
	PC 18:0/22:6																1.4				1.1	
PC 40:5	PC 18:0/22:5 (a)	PC 16:0/24:5 | 20:1/20:4							1.1						2.2		1.6		1.7	*	1.5	
	PC 20:0/20:5	PC 20:1/20:4							1.7	*					3.4		2.0		3.1	**	3.3	*
	PC 18:0/22:5 (b)																				-2.3	
PI 38:6	PI 18:1/20:5																		-5.5	**	-4.8	**
	PI 16:0/22:6																		-4.0	**	-2.1	**
PC 40:4	PC 20:1/20:3 (a)						N/A	**							-1.7				-1.2		3.0	*
	PC 20:1/20:3 (b)	PC 18:0/22:4 | 18:1/22:3	2.0	*											3.3	*	2.6	**				
	PC 18:0/22:4																		4.6	**	3.7	**
	PC 20:0/20:4		-1.1				N/A	*							11.6		4.3	**	11.8	*	10.1	**
PI 38:5	PI 18:1/20:4	PI 18:1/20:4																	-3.6	**	-2.7	**
	PI 18:0/20:5	PI 18:0/20:5																	-8.2	**	-6.8	**
PI 38:4	PI 18:0/20:4 (a)				1.2																	
	PI 18:0/20:4 (b)				2.2	*																
PC 40:2	PC 20:1/20:1																		N/A	**		
	PC 18:1/22:1																		N/A	**		
PC 42:10	PC 20:5/22:5		1.4				2.4	*	1.9						2.5		-1.1		5.0	**	2.7	*
	PC 20:4/22:6 (a)		1.5				1.2		2.3	*					1.6		1.3	*	2.0	*	3.0	*
	PC 20:4/22:6 (b)		2.0	*			2.7	**	3.2	*					4.7	*	3.5	*	5.9	**	3.6	**
PC 42:9	PC 20:4/22:5						6.9	**							4.7	*	1.7	*			7.4	**
	PC 20:5/22:4																4.0	*	21.2	**		
	PC 20:3/22:6						1.9								1.1		2.2	**	1.8	*	3.5	**
PC 42:7	PC 20:2/22:5																				1.4	
	PC 20:1/22:6																				1.4	
PC 42:6	PC 20:0/22:6 (a)	PC 20:2/22:4													-1.1				1.1		1.3	
	PC 20:2/22:4																1.0					
	PC 20:1/22:5														-1.1		1.2		-1.5		1.4	
	PC 20:0/22:6 (b)														2.2		3.9	**	1.9		3.3	*
PC 42:4	PC 20:1/22:3		1.5																			
	PC 18:1/24:3	PC 22:3/20:1			2.4	**																
	PC 18:3/24:1		1.5		1.8	**																
PC 42:3	PC 18:1/24:2				1.3	*																
	PC 18:2/24:1				1.3																	
PI 40:4	PI 18:0/22:4																		2.2	*		
	PI 20:0/20:4																		4.5	**		
PC 44:10	PC 22:5/22:5																		5.3	**	2.7	**
	PC 20:5/24:5																				2.2	
	PC 22:4/22:6																		12.2	**	5.7	**
PC 44:7	PC 20:5/24:2				1.6																	
	PC 22:6/22:1				-1.3	*																
PC 44:5	PC 20:4/24:1 (a)		1.1		2.0	*																
	PC 20:4/24:1 (b)		2.0	*	2.0	**																
	PC 20:5/24:0		1.1		1.1																	
PC 44:4	PC 20:3/24:1				1.8	*																
	PC 20:4/24:0				2.9	*																
PC 44:3	PC 20:2/24:1 (a)	PC 18:1/26:2			1.4																	
	PC 20:2/24:1 (b)	PC 18:2/26:1			1.2																	
PC 46:6	PC 24:1/22:5				1.3																	
	PC 20:5/26:2				1.0																	
PC 46:5	PC 20:4/26:1 (a)	PC 22:4/24:1	1.6		2.7	**																
	PC 20:4/26:1 (b)	PC 22:5/24:0			1.9	**																
	PC 22:5/24:0		-1.2																			

Supplementary Figure S1. Individual lipid isomers detected using LC-MS/MS triple quad mass spectrometry, across 5 tissues and two timepoints. Data is given as absolute peak area (red low, green high area counts) and fold change within a lipid structure (i.e. PC 42:10). The secondary ID indicates a co-eluting peak was detected. For fold changes, green represents GM diet up regulation, with red representing control diet up regulation. For t-test scores, ** < 0.01, * 0.01-0.05, with red values being those which fell below the Benjamini-Hochberg multiple-test critical value.

Simple ID	Primary ID	Secondary ID	Brain (%)						Eye (%)						Gill (%)						Intestine (%)						Liver (%)						Brain		Eye		Gill		Intestine		Liver	
			Int.			Final			Int.			Final			Int.			Final			Int.			Final			Int.			Final			Int.	Final	Int.	Final	Int.	Final	Int.	Final	Int.	Final
			C	G	p	C	G	p	C	G	p	C	G	p	C	G	p	C	G	p	C	G	p	C	G	p	C	G	p	C	G	p	Percentage change									
PE 34:2	PE 16:1/18:1					67.1	61.3																											-5.8								
	PE 16:0/18:2					32.9	38.7																											5.8								
PE O-36:5	PE O-18:3/18:2											44.8	49.7																							4.9						
	PE P-16:0/20:4											55.2	50.3																							-4.9						
PE O-36:3	PE O-18:2/18:1											94.7	92.0																							-2.7						
	PE P-18:0/18:2											5.3	8.0																							2.7						
PE 36:6	PE 16:1/20:5																							39.2	0.0	**														-39.2		
	PE 14:0/22:6																							60.8	0.0	**														-60.8		
PE 36:4	PE 18:1/18:3					24.9	27.6																											2.7								
	PE 16:0/20:4					75.1	72.4																											-2.7								
PE 36:3	PE 18:1/18:2																				77.4	52.3	**	71.9	50.9	**													-25.1	-21.0		
	PE 16:0/20:3																				22.6	47.7	**	28.1	49.1	**													25.1	21.0		
PE 36:2	PE 18:1/18:1					93.5	92.4		91.4	78.6	*	92.7	86.5	*							71.2	27.8	**	64.3	18.3	**				76.7	29.2	**		-1.0	-12.8	-6.2			-43.5	-46.0		-47.5
	PE 18:0/18:2 (a)	PE 16:0/20:2				6.5	7.6		8.6	21.4	*	7.3	13.5	*							28.8	72.2	**	21.5	12.4					23.3	70.8	**		1.0	12.8	6.2			43.5	-9.1		47.5
	PE 18:0/18:2 (b)																							14.2	69.3	**														55.2		
PE O-38:7	PE O-18:3/20:4											2.5	17.2	**																						14.7						
	PE O-18:2/20:5					85.0	75.7	**				76.5	59.6	**				61.8	42.5	*														-9.3		-16.9		-19.4				
	PE P-16:0/22:6					15.0	24.3	**				21.0	23.2					38.2	57.5	*														9.3		2.2		19.4				
PE O-38:6	PE P-18:1/20:4								61.8	72.7																									10.8							
	PE P-18:0/20:5								38.2	27.3																									-10.8							
PC 30:1	PC 14:0/16:1 (a)														35.1	34.7		69.4	100.0	**																	-0.3	30.6				
	PC 14:0/16:1 (b)														40.0	41.1		30.6	0.0	**																	1.0	-30.6				
	PC 14:1/16:0														24.9	24.2																					-0.7					
PE O-38:5	PE O-18:2/20:3											48.8	31.0	**																						-17.8						
	PE P-18:0/20:4 (a)		32.7	34.4																													1.7									
	PE P-18:0/20:4 (b)		67.3	65.6								51.2	69.0	**																			-1.7			17.8						
PE 38:7	PE 18:2/20:5	PE 18:3/20:4	49.0	58.5		42.4	59.9	**				78.0	86.1	*										80.6	94.9	**							9.5	17.6		8.1				14.2		
	PE 16:1/22:6		51.0	41.5		57.6	40.1	**				22.0	13.9	*										19.4	5.1	**							-9.5	-17.6		-8.1				-14.2		
PE 38:6	PE 18:2/20:4								3.7	20.6	**	5.6	15.5	**							7.9	10.3		2.9	9.9	**	2.5	31.1	**	4.4	23.6	**			17.0	9.9			2.4	7.0	28.6	19.2
	PE 18:1/20:5	PE 18:2/20:4							31.0	11.7	**	34.0	19.2	**				21.0	7.5	**	17.8	7.8		37.3	9.9	**	42.7	12.6	**	40.4	19.6	**			-19.3	-14.8		-13.5	-10.0	-27.4	-30.1	-20.8
	PE 16:0/22:6								65.4	67.7		60.4	65.3					79.0	92.5	**	74.3	81.9		59.9	80.3	**	54.8	56.3		55.2	56.8				2.3	4.9		13.5	7.6	20.4	1.5	1.6
PE 38:5	PE 16:0/22:5	PE 18:1/20:4							42.1	54.3	*							56.4	58.4					34.1	37.4	*	25.0	37.4	**	26.5	40.1	**			12.3			2.0		3.3	12.3	13.6
	PE 18:0/20:5								57.9	45.7	*							43.6	41.6					65.9	62.6	*	75.0	62.6	**	73.5	59.9	**			-12.3			-2.0		-3.3	-12.3	-13.6
PG 36:6	PG 16:1/20:5																							49.8	0.0	**														-49.8		
	PG 14:0/22:6																							50.2	0.0	**														-50.2		
PE 38:4	PE 18:1/20:3		36.9	37.3		42.0	37.3														16.9	2.3	**							27.1	4.0	**	0.4	-4.7					-14.6			-23.1
	PE 16:0/22:4																				8.1	8.7		11.1	11.2														0.6	0.1		
	PE 18:0/20:4		63.1	62.7		58.0	62.7														75.0	89.0	**	88.9	88.8					67.2	90.7	**	-0.4	4.7					14.0	-0.1		23.5
	PE 18:3/22:5																													5.6	5.3											-0.3
PE 38:3	PE 18:2/20:1																				61.5	39.8	*																-21.6			
	PE 18:0/20:3																				38.5	60.2	*																21.6			
	PE 18:1/20:2					36.3	41.3																											5.0								
	PE 16:1/22:2					63.7	58.7																											-5.0								
PE O-40:7	PE P-18:1/22:5					47.3	41.8					35.4	28.0					30.5	15.9	*														-5.5		-7.3		-14.6				
	PE P-18:0/22:6					52.7	58.2					64.6	72.0					69.5	84.1	*														5.5		7.3		14.6				
PC 32:2	PC 14:0/18:2																				43.2	78.1	**																34.9			
	PC 16:1/16:1																				56.8	21.9	**																-34.9			
PC 32:1	PC 16:0/16:1 (a)	PC 14:0/18:1							66.0	57.5	**	74.5	58.1	**	50.8	38.5	**	70.0	44.4	**															-8.5	-16.4	-12.3	-25.6				
	PC 16:0/16:1 (b)								27.2	28.3		21.1	30.0	*	36.9	45.2	**	25.5	43.4	**															1.1	8.8	8.3	17.9				
	PC 16:0/16:1 (c)	PC 14:0/18:1							6.8	14.2	**	4.3	11.9	**	2.7	13.4	**	4.5	12.2	**															7.4	7.6	10.7	7.7				
	PC 16:0/16:1 (d)														9.5	2.9	**																				-6.7					
PE 40:9	PE 20:4/20:5		12.5	16.4		35.5	18.4					14.9	6.5	*													6.5	5.6					3.9	-17.0		-8.5					-0.9	
	PE 18:3/22:6		87.5	83.6		64.5	81.6					85.1	93.5	*													93.5	94.4					-3.9	17.0		8.5					0.9	
PE 40:8	PE 20:3/20:5	PE 20:4/20:4	20.2	31.5	**	25.7	42.8	**				6.7	11.4	*																4.8	7.6		11.3	17.1		4.7						2.8
	PE 18:2/22:6		79.8	68.5	**	74.3	57.2	**				93.3	88.6	*																95.2	92.4		-11.3	-17.1		-4.7						-2.8
PE 40:7	PE 18:2/22:5																							6.4	18.4	**	3.3	15.5	**	5.3	14.1	*								12.0	12.3	8.8
	PE 18:3/22:4																				1.8	10.4	**																8.6			
	PE 20:2/20:5		1.7	4.3	*	3.4	6.0	*				4.5	7.2	**							3.3	5.8	**	5.5	10.2	*	5.3	20.1	**	4.9	13.7	*	2.6	2.6		2.7			2.5	4.7	14.8	8.9
	PE 18:1/22:6 (a)		94.3	91.6	*	96.6	94.0	*				95.5	92.8	**							93.4	76.5	**	88.1	71.4	**	91.5	64.4	**	89.9	72.2	**	-2.7	-2.6		-2.7			-16.9	-16.7	-27.1	-17.7
	PE 18:1/22:6 (b)		4.0	4.1																	1.5	7.3	**										0.1						5.8			
PE 40:6	PE 18:1/22:5 (a)																							13.7	5.2	**				11.0	4.3	**								-8.5		-6.7
	PE 20:1/20:5																										19.7	8.5	**	12.7	9.6										-11.2	-3.1
	PE 18:1/22:5 (b)																													5.4	1.1	**										-4.3
	PE 18:0/22:6																							86.3	94.8	**	80.3	91.5	**	70.9	85.0	**								8.5	11.2	14.1
PC 33:1	PC 16:0/17:1 (a)	PC 15:0/18:1													71.8	67.3																					-4.5					
	PC 16:0/17:1 (b)														28.2	32.7																					4.5					
PE 40:5	PE 18:0/22:5 (a)	PE 20:1/20:4																			66.1	76.1	*				81.2	86.5	*	75.7	82.3								10.0		5.3	6.6
	PE 20:4/20:1	PE 18:1/22:4										37.6	26.4	*																						-11.1						
	PE 20:0/20:5																				11.9	10.5					18.8	13.5	*	9.9	13.3								-1.4		-5.3	3.4
	PE 18:0/22:5 (b)											62.4	73.6	*							22.0	13.4	*							14.4	4.4	**				11.1			-8.6			-9.9
PG 38:6	PG 18:1/20:5																							42.4	15.7	**				28.9	15.2	*								-26.7		-13.7
	PG 16:0/22:6																							57.6	84.3	**				71.1	84.8	*								26.7		13.7
PE 40:4	PE 20:1/20:3																										76.9	11.3	**	0.0	19.8	**									-65.6	19.8
	PE 18:1/22:3	PE 20:1/20:3	40.9	39.6																	21.5	23.4											-1.3						1.9			
	PE 18:0/22:4		59.1	60.4					78.2	72.7											66.4	55.4	**	65.3	57.6		14.8	44.2	**	0.0	37.7	**	1.3		-5.4				-11.1	-7.7	29.4	37.7
	PE 20:0/20:4								21.8	27.3											12.1	21.2	**	34.7	42.4		8.3	44.5	**	0.0	42.5	**			5.4				9.1	7.7	36.2	42.5
PC 34:4	PC 16:1/18:3																							59.2	54.0															-5.1		
	PC 16:0/18:4																							40.8	46.0															5.1		
PC 34:2	PC 16:1/18:1					30.7	18.2	**							15.7	3.3	**							19.7	3.4	**								-12.4			-12.4			-16.4		
	PC 16:0/18:2 (a)					43.4	53.0	*							79.6	76.7					97.9	87.0	**	80.3	96.6	**								9.6			-2.9		-10.9	16.4		
	PC 16:0/18:2 (b)	PC 14:0/20:2				26.0	28.8								4.7	20.0	**				2.1	13.0	**											2.8			15.3		10.9			
PE 42:10	PE 20:5/22:5		22.6	14.5	*	30.8	14.9																										-8.2	-15.9								
	PE 20:4/22:6 (a)		47.8	49.4		37.0	51.4		59.4	57.2		62.6	63.3								82.4	64.2	**										1.7	14.4	-2.3	0.7			-18.3			
	PE 20:4/22:6 (b)		29.6	36.1		32.2	33.8		40.6	42.8		37.4	36.7								17.6	35.8	**										6.5	1.6	2.3	-0.7			18.3			
PE 42:8	PE 20:2/22:6 (a)		64.6	65.2								86.9	86.1														93.2	82.2	**				0.5			-0.8					-11.0	
	PE 20:2/22:6 (b)		35.4	34.8								13.1	13.9														6.8	17.8	**				-0.5			0.8					11.0	
PE 42:6	PE 20:1/22:5								14.1	12.2		19.5	23.0								26.4	10.4	**	39.3	24.9	**	24.7	14.7	**	14.9	6.3	*			-1.9	3.5			-16.0	-14.4	-10.0	-8.5
	PE 20:0/22:6								85.9	87.8		80.5	77.0								73.6	89.6	**	60.7	75.1	**	75.3	85.3	**	85.1	93.7	*			1.9	-3.5			16.0	14.4	10.0	8.5
PE 42:5	PE 20:1/22:4																				84.3	74.1		69.1	59.4	*													-10.3	-9.7		
	PE 20:0/22:5																				15.7	25.9		30.9	40.6	*													10.3	9.7		
PC 36:6	PC 18:3/18:3											4.5	44.5	**										4.5	53.9	**	4.3	21.6	**	3.0	41.4	**				40.0				49.4	17.3	38.4
	PC 16:1/20:5 (a)								26.9	24.4																									-2.5							
	PC 16:1/20:5 (b)	PC 18:2/18:4	22.1	24.0		30.7	28.9		13.8	19.9		42.2	20.1	**	19.6	33.3		30.6	25.9		26.0	26.8		46.2	17.4	**	19.0	17.8		25.1	16.0	**	1.9	-1.8	6.1	-22.1	13.7	-4.7	0.8	-28.8	-1.3	-9.1
	PC 14:0/22:6		77.9	76.0		69.3	71.1		59.3	55.6		53.3	35.4	*	80.4	66.7		69.4	74.1		74.0	73.2		49.4	28.7	**	76.7	60.7	**	71.9	42.6	**	-1.9	1.8	-3.7	-17.9	-13.7	4.7	-0.8	-20.7	-16.0	-29.2
PC 36:5	PC 18:2/18:3																				6.6	5.4		5.8	13.9	**													-1.2	8.2		
	PC 16:0/20:5																				93.4	94.6		94.2	86.1	**													1.2	-8.2		
PC 36:4	PC 18:2/18:2	PC 16:1/20:3							16.1	6.5	**	12.4	7.9	**	12.3	6.4	*	6.7	3.4		32.5	13.4	**	20.4	14.5	**	28.9	15.6	**	25.1	16.8	**			-9.6	-4.6	-5.9	-3.3	-19.1	-5.9	-13.4	-8.3
	PC 18:1/18:3					14.3	11.9		31.7	10.3	**	26.6	16.3	**	26.8	9.3	**	16.6	14.4		36.5	10.5	**	42.2	20.4	**	43.3	14.5	**	38.4	23.7	*		-2.3	-21.3	-10.3	-17.5	-2.2	-26.0	-21.8	-28.8	-14.7
	PC 16:0/20:4 (a)					85.7	88.1		7.8	6.3		9.4	6.2	**	60.9	84.3	**	9.1	6.2	*	7.8	6.3		16.1	12.2	**	11.8	9.1		15.8	10.2	*		2.3	-1.5	-3.1	23.4	-2.9	-1.5	-3.8	-2.7	-5.6
	PC 16:0/20:4 (c)																										3.2	1.9													-1.3	
	PC 16:0/20:4 (b)								44.5	76.9	**	51.6	69.6	**				67.6	76.0		23.2	69.9	**	21.3	52.8	**	12.8	58.9	**	20.7	49.3	**			32.4	18.0		8.4	46.7	31.5	46.1	28.7

Simple ID	Primary ID	Secondary ID	Brain (%)						Eye (%)						Gill (%)						Intestine (%)						Liver (%)						Brain		Eye		Gill		Intestine		Liver	
			Int.			Final			Int.			Final			Int.			Final			Int.			Final			Int.			Final			Int.	Final	Int.	Final	Int.	Final	Int.	Final	Int.	Final
			C	G	p	C	G	p	C	G	p	C	G	p	C	G	p	C	G	p	C	G	p	C	G	p	C	G	p	C	G	p	Percentage change									
PC 36:3	PC 18:1/18:2											62.8	31.4	**	79.1	50.6	**				70.3	36.2	**	61.3	28.1	**	70.0	33.7	**	65.3	36.8	**				-31.5	-28.5		-34.1	-33.3	-36.3	-28.5
	PC 16:0/20:3 (a)	PC 18:0/18:3|18:1/18:2										5.6	17.6	*	19.9	31.1	*				22.8	47.0	**	34.4	58.3	**	29.4	49.8	**	34.7	63.2	**				12.0	11.2		24.2	23.9	20.4	28.5
	PC 16:0/20:3 (b)														1.0	18.3	**				6.9	16.7	**	4.3	13.7	*	0.6	16.5	**								17.3		9.9	9.4	15.9	
	PC 16:0/20:3 (c)											25.2	34.0	*																						8.8						
	PC 18:0/18:3											6.4	17.0	**																						10.6						
PC 36:2	PC 18:1/18:1								70.5	34.5	**	68.5	44.8	**	66.3	30.9	**	70.5	39.7	**	59.5	16.6	**	71.6	28.6	**	51.4	23.7	**	59.0	26.4	**			-36.1	-23.7	-35.4	-30.7	-42.9	-43.0	-27.8	-32.6
	PC 16:0/20:2 (a)								12.5	15.6		11.0	15.2	*	12.0	17.6		8.2	15.3	**	8.5	13.8	*	9.6	17.5		22.8	21.5		13.5	19.8				3.1	4.2	5.6	7.1	5.3	7.9	-1.4	6.3
	PC 16:0/20:2 (b)	PC 18:0/18:2							17.0	49.9	**	20.6	40.0	**	21.7	51.5	**	21.3	44.9	**										27.5	53.8	**			33.0	19.5	29.8	23.6				26.3
	PC 18:0/18:2																				32.0	69.6	**	18.8	53.9	**	25.7	54.9	**										37.6	35.2	29.2	
PC O-38:6	PC P-18:0/20:5											68.0	52.5	*				36.7	24.1																	-15.4		-12.5				
	PC O-16:0/22:6											32.0	47.5	*				63.3	75.9																	15.4		12.5				
PC 38:8	PC 18:3/20:5																							89.4	94.0															4.5		
	PC 16:2/22:6																							10.6	6.0															-4.5		
PC 38:7	PC 18:3/20:4																													1.7	6.5	*										4.8
	PC 18:2/20:5																										53.6	85.2	**	64.0	77.5	*									31.7	13.4
	PC 16:1/22:6 (a)																										31.4	7.4	**	24.1	8.0	**									-24.0	-16.1
	PC 16:1/22:6 (b)																										15.1	7.4	**	10.2	8.1										-7.7	-2.1
PC 38:6	PC 18:3/20:3																							2.1	5.1	**														3.0		
	PC 18:2/20:4														0.0	7.3	**				0.8	8.0	**	1.2	6.3	*	1.1	15.7	**								7.3		7.2	5.1	14.6	
	PC 18:1/20:5														20.2	8.0	**	19.4	7.7	**	16.0	10.8	*	34.4	12.1	**	17.3	12.5	*								-12.2	-11.7	-5.3	-22.4	-4.8	
	PC 16:0/22:6														79.8	84.7	*	80.6	92.3	**	83.1	81.2		62.3	76.5	**	81.6	71.9	**								4.9	11.7	-1.9	14.3	-9.8	
PC 38:5	PC 18:1/20:4 (a)																										7.7	4.1	**												-3.7	
	PC 18:1/20:4 (b)	PC 16:0/22:5																									51.5	51.3													-0.2	
	PC 16:0/22:5 (a)	PC 18:1/20:4							58.5	58.7											43.2	35.2	*												0.2				-8.0			
	PC 18:0/20:5								41.5	41.3											47.0	58.8	**				40.8	44.7							-0.2				11.8		3.9	
	PC 16:0/22:5 (b)																				9.8	6.0	**																-3.8			
PC 38:4	PC 18:2/20:2																				26.9	6.9	**				35.4	10.3	**	25.5	10.6	*							-20.0		-25.1	-14.8
	PC 18:1/20:3																	14.2	4.9	**				42.5	24.3	**												-9.3		-18.2		
	PC 18:3/20:1	PC 18:1/20:3																			30.2	13.5	**				34.8	22.4	**	27.5	17.9	*							-16.7		-12.3	-9.6
	PC 16:0/22:4											14.4	16.4		15.4	30.8	**	16.8	19.0					17.1	19.8		11.0	17.8	**							2.1	15.3	2.3		2.7	6.8	
	PC 18:0/20:4 (a)	PC 16:0/22:4							11.6	17.4	*	85.6	83.6		84.6	69.2	**	69.1	76.1		42.9	79.6	**	40.3	55.9	**	18.8	49.4	**	17.7	17.8				5.8	-2.1	-15.3	7.0	36.7	15.5	30.6	0.1
	PC 18:0/20:4 (b)								88.4	82.6	*																			29.4	53.7	**			-5.8							24.3
PC 38:3	PC 18:2/20:1	PC 18:1/20:2																			78.0	55.8	**				90.5	65.6	**										-22.2		-24.9	
	PC 18:0/20:3																				22.0	44.2	**				9.5	34.4	**										22.2		24.9	
PC 38:2	PC 18:1/20:1																										82.9	52.8	**												-30.1	
	PC 18:0/20:2																										17.1	47.2	**												30.1	
PG 44:11	PG 22:5/22:6 (a)																										97.3	90.7	**												-6.5	
	PG 22:5/22:6 (b)																										2.7	9.3	**												6.5	
PC 40:9	PC 20:4/20:5 (a)								16.0	22.6		31.8	17.9	**							15.8	23.3	*	32.4	16.6	*	12.3	26.3	**	27.5	20.5				6.6	-13.9			7.5	-15.8	14.0	-7.0
	PC 18:3/22:6								84.0	77.4		68.2	82.1	**							84.2	76.7	*	67.6	83.4	*	85.0	60.4	**	72.5	79.5				-6.6	13.9			-7.5	15.8	-24.7	7.0
	PC 20:4/20:5 (b)																										2.7	13.3	**												10.6	
PC 40:8	PC 18:3/22:5								3.9	10.0	**	9.7	16.3								4.8	7.5	*	14.2	20.4	**	4.8	11.2	**	7.1	16.0	**			6.1	6.6			2.6	6.1	6.4	8.9
	PC 20:3/20:5	PC 20:4/20:4	14.6	28.7	*				12.0	27.1	**	17.8	25.3	*							10.1	22.9	**	16.3	22.2		7.2	30.7	**	12.9	28.2	**	14.1		15.1	7.5			12.8	5.8	23.5	15.2
	PC 18:2/22:6 (a)		85.4	71.3	*				84.1	62.9	**	72.5	58.4	*							85.1	69.6	**	68.5	52.5	**	88.0	58.1	**	80.0	55.9	**	-14.1		-21.2	-14.0			-15.5	-16.0	-29.9	-24.1
	PC 18:2/22:6 (b)																							0.9	4.9	**														4.0		
PC 40:7	PC 18:2/22:5								3.5	14.6	**	6.7	15.7	**							6.1	18.9	**	12.1	28.4	**	8.2	25.4	**						11.1	9.0			12.9	16.4	17.2	
	PC 20:2/20:5	C20:3/20:4							3.7	16.6	**	6.8	13.1	**							6.9	23.4	**	5.3	13.6	**	7.4	32.3	**						12.9	6.3			16.5	8.3	25.0	
	PC 18:1/22:6 (a)		63.0	73.9	**				35.1	36.3		27.4	33.3		36.4	45.2		31.6	49.9		87.0	57.7	**	82.6	57.9	**	84.4	42.3	**				11.0		1.2	5.9	8.8	18.3	-29.3	-24.7	-42.2	
	PC 18:1/22:6 (b)		37.0	26.1	**				57.7	32.5	**	59.1	37.9	**	63.6	54.8		68.4	50.1														-11.0		-25.2	-21.2	-8.8	-18.3				
PC 40:6	PC 18:1/22:5																							29.6	8.7	**														-20.8		
	PC 20:1/20:5																							16.8	21.2	*				22.0	24.4									4.4		2.3
	PC 18:0/22:6																							53.7	70.1	**				78.0	75.6									16.4		-2.3
PC 40:5	PC 18:0/22:5 (a)	PC 16:0/24:5 | 20:1/20:4										64.2	53.7	*							80.2	73.0	**	80.4	76.6		81.5	70.3	**	80.1	76.5					-10.5			-7.2	-3.8	-11.1	-3.6
	PC 20:0/20:5	PC 20:1/20:4										35.8	46.3	*							19.8	27.0	**	19.6	23.4		18.5	29.7	**	9.0	20.6	**				10.5			7.2	3.8	11.1	11.6
	PC 18:0/22:5 (b)																													11.0	2.9	**										-8.0
PI 38:6	PI 18:1/20:5																										62.3	54.2		58.4	41.0										-8.1	-17.4
	PI 16:0/22:6																										37.7	45.8		41.6	59.0										8.1	17.4
PC 40:4	PC 20:1/20:3 (a)								0.0	40.9	**										71.8	20.6	**				78.3	32.0	**	46.1	32.2				40.9				-51.2		-46.3	-14.0
	PC 20:1/20:3 (b)	PC 18:0/22:4 | 18:1/22:3	46.3	65.1	**																20.3	35.3	**	67.3	56.4	*							18.8						15.0	-10.9		
	PC 18:0/22:4																										15.5	34.2	**	40.9	35.2										18.7	-5.7
	PC 20:0/20:4		53.7	34.9	**				0.0	59.1	**										7.8	44.1	**	32.7	43.6	*	6.2	33.8	**	13.0	32.7	**	-18.8		59.1				36.3	10.9	27.6	19.7
PI 38:5	PI 18:1/20:4	PI 18:1/20:4																									45.5	66.6		52.5	73.6	**									21.1	21.2
	PI 18:0/20:5	PI 18:0/20:5																									54.5	33.4		47.5	26.4	**									-21.1	-21.2
PI 38:4	PI 18:0/20:4 (a)					66.7	55.8																											-11.0								
	PI 18:0/20:4 (b)					33.3	44.2																											11.0								
PC 40:2	PC 20:1/20:1																										32.6	0.0	**												-32.6	
	PC 18:1/22:1																										67.4	0.0	**												-67.4	
PC 42:10	PC 20:5/22:5		17.7	14.1					12.3	14.0		20.2	16.3								17.8	17.9		45.3	30.2		21.3	32.1		41.3	35.9		-3.5		1.7	-3.9			0.1	-15.1	10.9	-5.4
	PC 20:4/22:6 (a)		42.6	37.7					46.4	28.5	**	41.0	36.4								56.7	34.0	**	42.3	39.0		61.7	37.3	**	40.3	41.6		-4.8		-17.9	-4.6			-22.7	-3.3	-24.4	1.4
	PC 20:4/22:6 (b)		39.8	48.1					41.3	57.5	**	38.8	47.3	*							25.5	48.1	**	12.4	30.8	**	17.1	30.6	**	18.4	22.5	*	8.4		16.2	8.5			22.6	18.4	13.5	4.1
PC 42:9	PC 20:4/22:5								16.9	38.1	*										11.2	33.8	*	22.8	16.5					10.5	19.2	*			21.2				22.6	-6.3		8.7
	PC 20:5/22:4																							11.2	19.9		3.3	29.0	**											8.8	25.8	
	PC 20:3/22:6								83.1	61.9	*										88.8	66.2	*	66.1	63.6		96.7	71.0	**	89.5	80.8	*			-21.2				-22.6	-2.4	-25.8	-8.7
PC 42:7	PC 20:2/22:5																													12.0	12.6											0.6
	PC 20:1/22:6																													88.0	87.4											-0.6
PC 42:6	PC 20:0/22:6 (a)	PC 20:2/22:4																			38.8	26.8	*				19.0	17.0		21.0	12.8	*							-12.0		-2.0	-8.2
	PC 20:2/22:4																							20.7	11.3	*														-9.4		
	PC 20:1/22:5																				24.7	15.4	*	53.1	33.5	**	42.7	23.2	**	48.1	37.2								-9.3	-19.6	-19.6	-10.9
	PC 20:0/22:6 (b)																				36.5	57.8	**	26.3	55.2	**	38.3	59.8	**	30.9	50.0	**							21.3	28.9	21.5	19.1
PC 42:4	PC 20:1/22:3		41.7	39.6																													-2.2									
	PC 18:1/24:3	PC 22:3/20:1				26.8	32.4	**																										5.6								
	PC 18:3/24:1		58.3	60.4		73.2	67.6	**																									2.2	-5.6								
PC 42:3	PC 18:1/24:2					88.5	88.4																											-0.1								
	PC 18:2/24:1					11.5	11.6																											0.1								
PI 40:4	PI 18:0/22:4																										50.0	34.1													-15.9	
	PI 20:0/20:4																										50.0	65.9													15.9	
PC 44:10	PC 22:5/22:5																										67.8	47.8	*	51.5	44.6										-20.1	-6.9
	PC 20:5/24:5																													26.4	19.0											-7.4
	PC 22:4/22:6																										32.2	52.2	*	22.1	36.4	*									20.1	14.3
PC 44:7	PC 20:5/24:2					52.2	69.5																											17.3								
	PC 22:6/22:1					47.8	30.5																											-17.3								
PC 44:5	PC 20:4/24:1 (a)		40.4	33.9		25.8	27.4																										-6.5	1.6								
	PC 20:4/24:1 (b)		29.2	43.0		52.9	59.8	*																									13.8	7.0								
	PC 20:5/24:0		30.4	23.2		21.3	12.8	**																									-7.3	-8.5								
PC 44:4	PC 20:3/24:1					93.3	89.5	*																										-3.8								
	PC 20:4/24:0					6.7	10.5	*																										3.8								
PC 44:3	PC 20:2/24:1 (a)	PC 18:1/26:2				36.0	38.7																											2.6								
	PC 20:2/24:1 (b)	PC 18:2/26:1				64.0	61.3																											-2.6								
PC 46:6	PC 24:1/22:5					77.6	80.6																											3.0								
	PC 20:5/26:2					22.4	19.4																											-3.0								
PC 46:5	PC 20:4/26:1 (a)	PC 22:4/24:1	52.2	69.0	**	54.6	64.1	*																									16.7	9.5								
	PC 20:4/26:1 (b)	PC 22:5/24:0				45.4	35.9	*																										-9.5								
	PC 22:5/24:0		47.8	31.0	**																												-16.7									

Supplementary Figure S2. Individual lipid isomers detected using LC-MS/MS triple quad mass spectrometry, across 5 tissues and two timepoints. Data is given as percentage composition and percentage change within a lipid structure (i.e. PC 42:10), with the percentage point change relative to the control. The secondary ID indicates a co-eluting peak was detected. For percentage point changes, green represents GM diet up regulation, with red representing control diet up regulation. For t-test scores, ** < 0.01, * 0.01-0.05, with red values being those which fell below the Benjamini-Hochberg multiple-test critical value.
