# Supplementary material for: Profiling Phospholipids within Atlantic Salmon Salmo salar with Regards to a Novel Terrestrial Omega-3 Oil Source
Source: Metabolites. 2022 Sep 10;12(9):851. doi: 10.3390/metabo12090851 (PMC9503986; doi:10.3390/metabo12090851)
Supplement: Supplementary file 1 [file metabolites-12-00851-s001.zip › Supplementary Tables S1-S10 SFC-QTof.pdf]

Supplementary Table S1. SFC-QTOF analysis of brain tissue at the final time point. Simple ID was designated by SFC-QTOF, triple quad ID was determined by LC-MS/MS. Green and red represent high and low peak areas, respectively. Red triple quad IDs mean multiple acyl isomers were detected.

m/z	RT	Anova (p)	q Value	Fold	Highest	Co.	GM	CV%	Simple ID	Triple Quad ID	Adducts	Formula	M.E (ppm)
688.5886	5.51	5.646E-08	6.09E-06	2.7	GM			8.82	Cer 42:4;O2		M-H, M+FA-H		
690.6045	5.54	1.534E-05	0.000317	1.6	GM			7.58	Cer 42:3;O2		M-H, M+FA-H		
797.5416	6.49	1.926E-07	1.56E-05	2.0	GM			9.57	MDGD 34:3		M+FA-H	C44H79O10P	9.718
823.5565	6.52	6.394E-07	4.16E-05	1.5	GM			4.18	MDGD 36:4	MGDG 20:1/16:3	M+FA-H		
849.5079	7.13	0.0006657	0.005099	1.8	GM			12.72	PG 42:6	PG 20:5/22:1	M-H2O-H	C50H77O10P	0.361
777.5077	7.19	0.0001039	0.001188	1.8	GM			12.61	PG P-38:6		M-H	C44H75O9P	0.197
852.6571	7.67	4.854E-06	0.000144	2.0	GM			12.47	HexCer 42:3;O2		M-H, M+FA-H		
850.6419	7.89	6.283E-09	4.32E-06	2.4	GM			7.24	HexCer 42:4;O2		M-H, M+FA-H		
852.6570	7.94	3.978E-05	0.000572	1.5	GM			8.26	HexCer 42:3;O2		M-H, M+FA-H		
854.6787	8.01	0.0094403	0.036903	1.3	Control			11.63	HexCer 42:2;O2		M+FA-H	C48H91NO8	7.487
815.4872	8.07	1.782E-07	1.56E-05	2.8	GM			12.77	PG 40:9	PG 18:3/22:6	M-H	C46H73O10P	0.371
866.6380	8.15	2.13E-08	4.41E-06	2.3	GM			7.08	HexCer 42:4;O3		M-H, M+FA-H		
843.5185	8.24	0.0039727	0.019213	2.3	GM			21.28	PG 42:9		M-H	C48H77O10P	0.416
796.5191	8.31	0.0070602	0.029652	1.4	GM			5.26	HexCer 36:5;O4		M+FA-H		
960.7415	8.37	0.0036085	0.018272	1.7	GM			13.56	HexCer 46:1;O5		M+FA-H		
771.5184	8.41	3.637E-09	4.32E-06	3.6	GM			11.29	PG 36:3	PG 16:0/20:3	M-H	C42H77O10P	0.327
986.7598	8.53	0.0033187	0.017421	1.6	GM			11.14	HexCer 48:2;O5		M+FA-H		
988.7746	8.64	2.727E-05	0.000459	2.1	GM			13.02	HexCer 48:1;O5		M+FA-H		
886.6092	8.68	8.433E-06	0.0002	1.6	GM			8.14			Neg		
966.7911	9.02	0.0167758	0.056003	1.3	GM			13.51	HexCer 50:2;O2		M+FA-H		
762.5067	9.10	0.0266999	0.128697	1.2	GM			8.7	PE 38:7	PE 18:2/20:5	M+H, M+Na	C43H72NO8P	-0.218
758.4773	9.17	9.896E-07	4.52E-05	2.7	GM			13.35	PE 38:8	PE 20:5/18:3	M-H	C43H70NO8P	0.933
734.4773	9.17	2.77E-05	0.000459	1.5	Control			7.15	PE 36:6	PE 16:1/20:5	M-H	C41H70NO8P	0.875
838.5394	9.21	0.0540012	0.213886	1.2	GM			10.51	PE 44:11	PE 22:5/22:6	M+H, M+Na	C49H76NO8P	1.574
844.4555	9.25	0.0004184	0.003547	1.2	GM			4.31			Neg		
784.4932	9.26	7.759E-07	4.47E-05	2.5	GM			13.65	PE 40:9	PE 18:3/22:6	M-H		
840.5510	9.29	3.691E-06	0.000113	2.0	GM			7.65	PE 44:10	PE 22:5/22:5	M+H	C49H78NO8P	-3.315
810.5075	9.30	0.003596	0.01827	1.3	GM			9.96	PE 42:10	PE 20:4/22:6	M-H	C47H74NO8P	-0.468
786.5084	9.35	0.0005757	0.004596	1.5	GM			13.23	PE 40:8	PE 20:3/20:5	M-H	C45H74NO8P	0.567
748.5290	9.39	0.0065188	0.042294	1.2	GM			5.69	PE O-38:7	PE P-18:1/20:5	M+H, M+Na	C43H74NO7P	1.982
856.5134	9.46	0.0016945	0.010522	1.6	Control			15.86	PE 42:10		M+FA-H		
738.5076	9.48	2.97E-05	0.000472	1.5	GM			9.09	PE 36:4	PE 16:0/20:4	M-H	C41H74NO8P	-0.381
788.5249	9.48	0.0017282	0.010687	1.3	Control			8.75	PE 40:7	PE 20:2/20:5	M-H	C45H76NO8P	1.644
812.5233	9.48	2.49E-06	9.17E-05	2.3	GM			11.42	PE 42:9	PE 20:3/22:6	M-H	C47H76NO8P	-0.285
770.5135	9.49	5.291E-05	0.000732	2.0	GM			14.35	PE O-40:9		M-H		
714.5085	9.53	0.000243	0.002335	1.3	Control			6.74	PE 34:2	PE 16:1/18:1	M-H	C39H74NO8P	0.815
696.4980	9.55	5.848E-06	0.000158	1.4	GM			7.13	PE O-34:4	PE P-16:0/18:3	M-H	C39H72NO7P	0.876
720.4980	9.55	0.0002562	0.002401	1.3	GM			5.6	PE O-36:6	PE P-16:0/20:5	M-H	C41H72NO7P	0.865
814.5350	9.59	0.003938	0.019123	1.4	GM			12.74	PE 42:8	PE 20:2/22:6	M-H	C47H78NO8P	-5.195
726.5438	9.61	0.0005622	0.005911	1.3	GM			3.21	PE O-36:4	PE P-18:0/18:3	M+H	C41H76NO7P	0.862
750.5445	9.62	0.0016192	0.013752	1.3	GM			6.03	PE O-38:6	PE P-18:0/20:5	M+H, M+Na	C43H76NO7P	1.669
728.5243	9.63	3.097E-07	2.34E-05	1.9	Control			6.64	PE 35:2		M-H	C40H76NO8P	0.941
722.5137	9.68	0.0001146	0.001271	1.6	GM			10.83	PE O-36:5	PE P-16:0/20:4	M-H	C41H74NO7P	1.013
794.5687	9.69	0.0005923	0.006112	1.4	GM			7.56	PE 40:5	PE 18:0/22:5|20:1/20:4	M+H	C45H80NO8P	-0.882
766.5367	9.74	1.872E-05	0.000357	1.5	GM			3.94	PE 38:4	PE 18:1/20:3	M-H	C43H78NO8P	-3.355
776.5589	9.74	0.0169305	0.091752	1.2	GM			5.43	PE O-40:7	PE P-18:0/22:6	M+H	C45H78NO7P	0.101
742.5404	9.76	0.002232	0.013002	1.3	Control			10.29	PE 36:2	PE 18:1/18:1	M-H	C41H78NO8P	1.630
810.5323	9.78	0.000443	0.003693	1.7	Control			12.44	PE 42:10		M-H	C47H74NO8P	29.998
796.5803	9.82	6.72E-06	0.000171	1.5	GM			2.2	PE 40:4		M+H	C45H82NO8P	-6.027
712.5292	9.85	9.281E-07	4.52E-05	1.6	Control			6.32	PE O-35:3		M-H	C40H76NO7P	0.692
686.5134	9.86	1.679E-06	6.85E-05	1.4	Control			4.72	PE O-33:2		M-H	C38H74NO7P	0.525
756.5522	9.90	7.045E-08	7.09E-06	1.9	Control			5.78	PE 37:2		M-H	C42H80NO8P	-3.482
750.5431	9.92	9.779E-07	4.52E-05	1.7	GM			3.94	PE O-38:5	PE P-18:0/20:4	M-H	C43H78NO7P	-1.641
820.5841	9.95	1.217E-06	5.25E-05	2.2	GM			4.26	PE 42:5	PE 20:4/22:1	M-H	C47H84NO8P	-2.508
770.5713	10.04	0.0003719	0.003243	1.4	Control			9.4	PE 38:2	PE 18:1/20:1	M-H	C43H82NO8P	1.018
766.5399	10.05	6.618E-07	4.16E-05	2.1	Control			8.49	PE 38:4		M-H	C43H78NO8P	0.828
776.5608	10.07	1.893E-05	0.000357	1.3	GM			4.19	PE O-40:6	PE P-18:0/22:5	M-H	C45H80NO7P	1.129
740.5550	10.13	4.999E-06	0.000145	1.4	Control			5.05	PE O-37:3		M-H, M+Cl	C38H76NO8P	-6.752
752.5604	10.13	2.26E-06	8.74E-05	1.7	GM			3.66	PE O-38:4	PE P-18:0/20:3	M-H	C43H80NO7P	0.604
820.5839	10.15	2.128E-05	0.000387	1.4	GM			1.9	PE 42:5		M-H	C47H84NO8P	-2.727
714.5448	10.16	3.164E-06	0.000109	1.5	Control			4.97	PE O-35:2	PE P-18:0/17:1	M-H	C40H78NO7P	0.613

778.5762	10.19	9.762E-09	4.32E-06	2.6	GM			6.47	PE O-40:5	PE P-18:0/22:4	M-H	C45H82NO7P	0.696
794.5709	10.24	1.579E-08	4.32E-06	2.0	Control			3.84	PE 40:4	PE 18:0/22:4	M-H	C45H82NO8P	0.437
848.6162	10.33	6.574E-07	4.16E-05	1.8	GM			6.67	PE 44:5	PE 20:4/24:1	M-H	C49H88NO8P	-1.451
768.5559	10.37	6.627E-06	0.000174	1.7	Control			5.81	PE 38:3	PE 18:1/20:2	M-H	C43H80NO8P	1.276
798.6017	10.37	0.0010356	0.007477	1.3	Control			9.15	PE 40:2	PE 18:1/22:1|20:1/20:1	M-H	C45H86NO8P	-0.206
702.5451	10.38	5.867E-05	0.00077	1.3	GM			6.52	PE O-34:1		M-H	C39H78NO7P	1.059
804.5922	10.38	2.003E-05	0.000368	1.5	GM			5.78	PE O-42:6	PC 16:0/18:1	M-H	C47H84NO7P	1.177
780.5923	10.44	7.101E-06	0.000176	1.4	GM			4.83	PE O-40:4	PE P-18:1/22:2	M-H	C45H84NO7P	1.287
857.5195	10.49	0.0003437	0.003069	1.3	GM			7.85	PI 36:4		M-H	C45H79O13P	1.081
796.5871	10.57	2.678E-06	9.62E-05	1.6	Control			4.95	PE 40:3	PE 18:1/22:2|20:1/20:2	M-H	C45H84NO8P	1.112
867.5421	10.60	0.0231619	0.069044	1.3	Control			16.74	PI O-38:6		M-H	C47H81O12P	3.279
859.5348	10.62	0.0010071	0.007314	1.4	GM			13.33	PI 36:3		M-H	C45H81O13P	0.728
869.5560	10.68	0.0041561	0.019845	1.3	GM			12.26	PI O-38:5	PI O-18:0/20:5	M-H	C47H83O12P	1.194
843.5400	10.69	0.0004631	0.003839	1.5	GM			13.97	PI O-36:4		M-H	C45H81O12P	0.826
885.5507	10.70	1.461E-05	0.000306	1.4	GM			5.4	PI 38:4	PI 18:0/20:4	M-H	C47H83O13P	0.913
887.5580	10.70	3.284E-05	0.000501	1.3	GM			6.85	PI 38:3		M-H	C47H85O13P	-8.401
913.5763	10.79	2.399E-06	9.05E-05	1.6	GM			8.24	PI 40:4		M-H	C49H87O13P	-5.255
887.5655	10.84	0.0029875	0.0161	1.4	GM			11.73	PI 38:3		M-H	C47H85O13P	0.003
871.5711	10.91	0.0044344	0.02078	1.4	GM			14.71	PI O-38:4	PI O-18:0/20:4	M-H	C47H85O12P	0.607
854.5674	14.80	3.831E-05	0.000683	1.9	GM			10.46	PC 42:10	PC 20:4/22:6	M+H	C50H80NO8P	-2.399
828.5520	14.81	1.082E-05	0.00024	3.0	GM			13.15	PC 40:9	PC 18:3/22:6	M+H, M+Na	C48H78NO8P	-2.208
880.5833	14.93	0.001984	0.015901	1.3	GM			4.26	PC 44:11	PC 22:5/22:6	M+H		
778.5369	15.03	0.0017007	0.014302	1.5	Control			10.68	PC 36:6	PC 16:1/20:5	M+H, M+Na	C44H76NO8P	-1.597
830.5677	15.07	0.0009493	0.008793	1.8	GM			18.21	PC 40:8	PC 18:2/22:6	M+H	C48H80NO8P	-2.101
856.5811	15.29	9.63E-08	2.05E-05	2.3	GM			6.05	PC 42:9	PC 20:3/22:6	M+H	C50H82NO8P	-4.641
832.5869	15.36	0.0017663	0.01478	1.2	Control			7.74	PC 40:7	PC 18:1/22:6	M+H, M+Na	C48H82NO8P	2.179
892.5334	15.37	0.3691298	0.282018	1.1	Control			14.04			Neg		
752.4630	15.38	0.4815341	0.322787	1.1	Control			2.97		PE 17:2/20:2	Neg		
858.5958	15.55	0.000108	0.001511	1.8	GM			7.75	PC 42:8		M+H	C50H84NO8P	-5.711
782.5699	15.60	1.275E-07	2.18E-05	2.0	GM			7.33	PC 36:4	PC 18:3/18:1	M+H, M+Na	C44H80NO8P	0.565
756.5529	15.65	1.475E-09	1.26E-06	3.1	GM			2.81	PC 34:3	PC 16:0/18:3	M+H	C42H78NO8P	-1.231
820.5809	15.77	1.61E-06	8.09E-05	1.8	Control			9.07	PC 39:6		M+H	C47H82NO8P	-5.060
830.5642	15.83	0.0037086	0.026489	1.3	GM			6.91	PC 40:8		M+H	C48H80NO8P	-6.313
758.5702	15.94	0.0005644	0.005911	1.2	GM			5.05	PC 34:2	PC 16:1/18:1	M+H	C42H80NO8P	1.034
848.6087	15.95	9.816E-07	5.98E-05	2.7	Control			13.86	PC 41:6		M+H	C49H86NO8P	-9.075
810.6001	16.22	2.707E-08	9.24E-06	2.4	GM			3.93	PC 38:4		M+H	C46H84NO8P	-0.799
886.6270	16.24	8.181E-08	2.05E-05	2.4	GM			7.08	PC 44:8		M+H	C52H88NO8P	-5.690
836.6154	16.32	1.107E-05	0.000242	1.4	GM			3.83	PC 40:5	PC 18:0/22:5	M+H	C48H86NO8P	-1.213
898.5771	16.33	0.0042945	0.020285	1.4	Control			13.71	PC 42:10		M+FA-H		
760.9930	16.41	0.0948962	0.296982	1.2	GM			11.12			Pos		
812.6150	16.42	4.493E-05	0.000752	1.3	GM			2.96	PC 38:3		M+H, M+K	C46H86NO8P	-1.649
768.5867	16.52	9.731E-09	4.15E-06	2.1	GM			5.4	PC O-36:4	PC O-16:0/20:4	M+H	C44H82NO7P	-4.484
888.6458	16.63	3.071E-05	0.00057	1.5	GM			8.3	PC 44:7	PC 20:5/24:2	M+H, M+Na	C52H90NO8P	-2.118
838.6284	16.77	3.895E-09	2.22E-06	2.4	GM			4.57	PC 40:4	PC 18:1/22:3	M+H	C48H88NO8P	-4.335
914.6589	16.84	0.0004197	0.004564	1.5	GM			9.93	PC 46:8		M+H	C54H92NO8P	-4.865
890.6261	16.87	0.1762447	0.194396	1.2	Control			12.09	PC O-42:7		M+FA-H		
864.6449	17.04	6.617E-07	5.97E-05	1.7	GM			5.16	PC 42:5	PC 20:4/22:1	M+H	C50H90NO8P	-3.230
926.6107	17.15	6.45E-05	0.000818	1.4	Control			4.57	PC 44:10		M+FA-H		
840.6465	17.21	4.808E-05	0.000789	1.4	GM			2.85	PC 40:3	PC 18:2/22:1	M+H	C48H90NO8P	-1.463
796.6172	17.27	1.035E-06	6.1E-05	1.9	GM			2.49	PC O-38:4	PC O-18:0/20:4	M+H	C46H86NO7P	-5.417
822.6346	17.47	0.0008255	0.007961	1.3	GM			5.16	PC O-40:5	PC O-18:0/22:5	M+H	C48H88NO7P	-3.061
866.6615	17.58	1.875E-07	2.41E-05	2.3	GM			9.13	PC 42:4	PC 18:3/24:1	M+H	C50H92NO8P	-2.083
914.6594	17.61	8.867E-06	0.000205	2.1	GM			10.85	PC 46:8		M+H	C54H92NO8P	-4.295
892.6789	17.63	2.199E-06	8.73E-05	2.2	GM			9.92	PC 44:5	PC 20:4/24:1	M+H	C52H94NO8P	-0.111
918.6958	17.78	0.0041752	0.029211	1.3	GM			6.24	PC 46:6	PC 24:1/22:5	M+H, M+Na	C54H96NO8P	1.291
868.6798	18.15	8.884E-05	0.001296	1.5	GM			7.95	PC 42:3	PC 18:1/24:2	M+H	C50H94NO8P	0.939
894.6909	18.21	2.846E-06	9.38E-05	2.0	GM			11.32	PC 44:4	PC 20:3/24:1	M+H	C52H96NO8P	-4.145
920.7073	18.28	9.205E-07	5.98E-05	2.2	GM			10.23	PC 46:5	PC 22:4/24:1	M+H	C54H98NO8P	-3.270
922.7012	18.29	0.0001303	0.001724	1.4	GM			9.21	PC 46:4		M+H		
946.7233	18.68	0.0003365	0.00376	1.4	GM			9.7	PC 48:6		M+H, M+Na		
896.7077	19.00	0.0003044	0.003441	1.3	GM			6.9	PC 44:3	PC 18:1/26:2	M+H	C52H98NO8P	-2.876

Supplementary Table S2. SFC-QTOF analysis of brain tissue at the intermediate time point. Simple ID was designated by SFC-QTOF, triple quad ID was determined by LC-MS/MS. Green and red represent high and low peak areas, respectively. Red triple quad IDs mean multiple acyl isomers were detected.

m/z	RT	Anova (p)	q Value	Fold	Highest	Co.	GM	CV%	Simple ID	Triple Quad ID	Adducts	Formula	M.E (ppm)
688.5892	5.40	0.0006	0.0500	2.0	GM			21.96	Cer 42:4;O2		M-H, M+FA-H		
690.6049	5.45	0.0080	0.2648	1.5	GM			15.79	Cer 42:3;O2		M-H, M+Cl, M+FA-H		
797.5418	6.38	0.0220	0.4541	1.5	GM			15.35	MGDG 34:3		M+FA-H		
852.6579	7.55	0.0020	0.1077	1.7	GM			18.52	HexCer 42:3;O2		M-H, M+FA-H		
850.6421	7.76	0.0012	0.0850	1.8	GM			14.41	HexCer 42:4;O2		M-H, M+FA-H		
839.4884	7.86	0.0143	0.3658	2.1	GM			33.3	PG 42:11	PG 20:5/22:6	M-H	C48H73O10P	1.829
815.4877	7.93	0.0008	0.0608	2.6	GM			31.73	PG 40:9		M-H	C46H73O10P	1.086
767.4872	7.98	0.0194	0.4307	2.2	GM			31.55	PG 36:5		M-H	C42H73O10P	0.388
817.5034	7.98	0.0167	0.3990	1.9	GM			30.21	PG 40:8		M-H	C46H75O10P	1.084
866.6391	8.02	0.0005	0.0456	1.8	GM			15.51	HexCer 42:4;O3		M-H, M+FA-H		
868.6529	8.08	0.0119	0.3386	1.4	GM			16.83	HexCer 42:3:O3		M+FA-H		
843.5183	8.10	0.0026	0.1294	3.0	GM			38.52	PG 42:9		M-H	C48H77O10P	0.151
797.5315	8.24	0.0019	0.1062	2.7	GM			28.58	PG 38:4		M-H	C44H79O10P	-2.925
771.5185	8.25	0.0000	0.0127	2.4	GM			15.07	PG 36:3		M-H	C42H77O10P	0.452
758.4771	8.99	0.0008	0.0607	1.8	GM			16.84	PE 38:8	PE 18:3/20:5	M-H	C43H70NO8P	0.679
734.4771	9.01	0.0147	0.3744	1.4	Control			14.81	PE 36:6	PE 16:1/20:5	M-H	C41H70NO8P	0.681
784.4932	9.09	0.0001	0.0160	1.9	GM			13.97	PE 40:9	PE 20:4/20:5	M-H	C45H72NO8P	1.123
810.5084	9.12	0.0034	0.1510	1.5	GM			14.4	PE 42:10	PE 20:5/22:5	M-H	C47H74NO8P	0.616
786.5086	9.16	0.0164	0.3945	1.3	GM			13.93	PE 40:8	PE 18:2/22:6	M-H	C45H74NO8P	0.872
838.5391	9.25	0.0001	0.0170	2.8	GM			17.41	PE 44:10	PE 22:5/22:5	M-H	C49H78NO8P	-0.109
856.5132	9.26	0.0001	0.0154	1.4	Control			4.61	PE 42:10		M+FA-H		
788.5252	9.27	0.0062	0.2335	1.3	Control			4.35	PE 40:7	PE 20:2/20:5	M-H	C45H76NO8P	2.026
738.5084	9.28	0.0000	0.0129	1.9	GM			7.29	PE 36:4	PE 16:0/20:4	M-H	C41H74NO8P	0.692
770.5135	9.29	0.0022	0.1149	1.6	GM			10.95	PE O-40:9		M-H		
812.5233	9.30	0.0000	0.0031	2.2	GM			10.59	PE 42:9	PE 20:3/22:6	M-H	C47H76NO8P	-0.399
696.4979	9.36	0.0015	0.0921	1.5	GM			12.78	PE O-34:4	PE P-16:0/18:3	M-H	C39H72NO7P	0.803
814.5354	9.39	0.0019	0.1062	1.4	GM			10.96	PE 42:8	PE 20:2/22:6	M-H	C47H78NO8P	-4.663
728.5242	9.44	0.0010	0.0698	1.5	Control			9.92	PE 35:2		M-H	C40H76NO8P	0.873
772.5294	9.45	0.0109	0.3231	1.3	Control			10.86	PE O-40:8	PE P-18:1/22:6	M-H	C45H76NO7P	0.990
722.5136	9.47	0.0001	0.0191	1.6	GM			8.1	PE O-36:5	PE P-18:0/18:4	M-H	C41H74NO7P	0.804
766.5389	9.53	0.0000	0.0134	1.8	GM			7.5	PE 38:4	PE 18:0/20:4	M-H	C43H78NO8P	-0.483
810.5309	9.55	0.0062	0.2335	1.4	Control			12.62	PE O-40:7		M-H, M+Cl	C45H78NO7P	12.805
712.5291	9.64	0.0016	0.0936	1.4	Control			10.97	PE O-35:3	PE O-18:2/17:1	M-H	C40H76NO7P	0.656
686.5134	9.66	0.0023	0.1158	1.3	Control			9.11	PE O-33:2		M-H	C38H74NO7P	0.501
750.5442	9.72	0.0002	0.0195	1.8	GM			9.47	PE O-38:5	PE P-18:0/20:4	M-H	C43H78NO7P	-0.132
820.5806	9.75	0.0000	0.0027	1.8	GM			8.75	PE 42:5	PE 20:4/22:1	M-H	C47H84NO8P	-6.749
784.5419	9.84	0.0002	0.0272	1.6	Control			11.61	PE 38:3;O		M-H		
766.5398	9.85	0.0008	0.0608	1.8	Control			11.69	PE 38:4		M-H	C43H78NO8P	0.686
796.5782	9.90	0.0018	0.0553	1.6	GM			11	PE 40:4		M+H	C45H82NO8P	-8.619
752.5581	9.91	0.0008	0.0610	1.7	GM			14.64	PE O-38:4	PE P-18:0/20:3	M-H		
740.5551	9.93	0.0223	0.4562	1.3	Control			13.01	PE O-37:3		M-H		
778.5757	9.97	0.0001	0.0154	2.4	GM			17.73	PE O-40:5	PE O-20:0/20:5	M-H	C45H82NO7P	0.059
794.5706	10.02	0.0003	0.0321	2.0	Control			13.68	PE 40:4	PE 18:1/22:3	M-H	C45H82NO8P	0.030
848.6155	10.11	0.0015	0.0936	1.6	GM			14.41	PE 44:5	PE 20:4/24:1	M-H	C49H88NO8P	-2.308
768.5558	10.15	0.0028	0.1346	1.6	Control			14.05	PE 38:3	PE 18:1/20:2	M-H	C43H80NO8P	1.183
857.5195	10.30	0.0069	0.2411	1.4	GM			7.28	PI 36:4	PI 16:0/20:4	M-H	C45H79O13P	1.145
796.5866	10.35	0.0070	0.2411	1.6	Control			15.93	PE 40:3	PE 18:1/22:2	M-H	C45H84NO8P	0.573
859.5313	10.40	0.0045	0.1867	1.4	GM			12.23	PI 36:3		M-H, M+Cl	C45H81O13P	-3.367
843.5398	10.48	0.0065	0.2387	1.5	GM			15.61	PI O-36:4		M-H	C45H81O12P	0.553

887.5609	10.48	0.0040	0.1701	1.3	GM			10.92	PI 38:3		M-H	C47H85O13P	-5.179
913.5760	10.58	0.0002	0.0195	1.6	GM			11.06	PI 40:4		M-H	C49H87O13P	-5.683
871.5707	10.70	0.0337	0.5484	1.4	GM			17.87	PI O-38:4	PI O-18:0/20:4	M-H	C47H85O12P	0.119
1593.9771	11.74	0.0443	0.6073	1.4	Control			21.28	CL 84:19		M-H	C93H144O17P2	-2.201
786.5073	14.54	0.0000	0.0094	2.3	GM			14.35	PC 32:4;O		M+FA-H		
846.5289	14.57	0.0001	0.0163	2.0	GM			13.58	PC 38:8	PC 20:5/18:3	M+FA-H	C46H76NO8P	-0.258
878.5727	14.76	0.0219	0.3186	1.4	Control			12.73	PC 44:12	PC 22:6/22:6	M+H, M+Na	C52H80NO8P	3.746
854.5678	14.85	0.0000	0.0001	2.3	GM			9.23	PC 42:10	PC 20:5/22:5	M+H	C50H80NO8P	-1.902
828.5515	14.87	0.0000	0.0001	2.2	GM			7.04	PC 40:9	PC 18;3/22:6	M+H	C48H78NO8P	-2.719
944.5670	15.01	0.0001	0.0160	1.7	Control			12.08			Neg		
778.5378	15.04	0.0001	0.0077	1.5	Control			8.13	PC 36:6	PC 14:0/22:6	M+H, M+Na	C44H76NO8P	-0.454
830.5687	15.12	0.0000	0.0013	1.6	GM			8.94	PC 40:8	PC 18:2/22:6	M+H	C48H80NO8P	-0.854
882.5966	15.18	0.0000	0.0001	2.5	GM			9.9	PC 44:10	PC 22:5/22:5	M+H	C52H84NO8P	-4.731
856.5817	15.30	0.0000	0.0001	2.0	GM			6.66	PC 42:9	PC 20:3/22:6	M+H	C50H82NO8P	-3.907
832.5880	15.39	0.0002	0.0091	1.6	Control			4.47	PC 40:7	PC 18:1/22:6	M+H, M+Na	C48H82NO8P	3.463
792.5540	15.50	0.0062	0.2335	1.3	GM			6.95	PC 32:1;O		M+FA-H		
858.5968	15.51	0.0001	0.0049	1.5	GM			8.54	PC 42:8		M+H	C50H84NO8P	-4.574
830.5641	15.61	0.0293	0.3969	1.3	GM			12.12	PC 40:8		M+H	C48H80NO8P	-6.436
782.5708	15.62	0.0000	0.0002	2.1	GM			9.23	PC 36:4	PC 16:0/20:4	M+H	C44H80NO8P	1.707
756.5531	15.65	0.0001	0.0076	2.1	GM			9.99	PC 34:3	PC 16:0/18:3	M+H	C42H78NO8P	-0.876
900.6100	15.71	0.0000	0.0000	15.0	Control			14.9	PC 44:9;O		M+H		
820.5803	15.78	0.0001	0.0049	1.4	Control			7.89	PC 39:6		M+H	C47H82NO8P	-5.890
836.5814	15.87	0.0171	0.4028	1.2	Control			10.56	PC O-38:6		M+FA-H	C46H82NO7P	0.375
848.5989	15.95	0.0000	0.0028	2.0	Control			8.03			Pos		
860.6159	15.97	0.0066	0.1369	1.4	Control			7.52	PC 42:7	PC 20:1/22:6	M+H	C50H86NO8P	-0.604
746.5679	16.16	0.0005	0.0213	1.3	Control			6.7	PC 33:1		M+H	C41H80NO8P	-2.080
810.6012	16.19	0.0000	0.0020	2.3	GM			14.36	PC 38:4	PC 18:0/20:4	M+H	C46H84NO8P	0.577
818.6051	16.20	0.0096	0.1809	1.5	Control			11.21	PC O-40:7	PC P-18:0/22:6	M+H	C48H84NO7P	-0.881
836.6158	16.30	0.0048	0.1163	1.4	GM			11.27	PC 40:5	PC 18:0/22:5	M+H, M+K	C48H86NO8P	-0.739
768.5881	16.51	0.0000	0.0001	1.8	GM			7.45	PC O-36:4	PC O-16:0/20:4	M+H	C44H82NO7P	-2.731
838.6281	16.79	0.0000	0.0010	2.0	GM			11.26	PC 40:4	PC 18:1/22:3	M+H, M+Na	C48H88NO8P	-4.700
774.6005	16.85	0.0147	0.2414	1.4	Control			11.33	PC 35:1	PC 17:0/18:1	M+H	C43H84NO8P	-0.240
840.6113	16.96	0.0014	0.0907	1.7	GM			12.48	PC O-38:4		M+FA-H	C46H86NO7P	-1.383
864.6467	17.02	0.0049	0.1163	1.4	GM			11.83	PC 42:5	PC 20:4/22:1	M+H	C50H90NO8P	-1.084
675.5373	17.45	0.0074	0.1488	1.7	Control			16.89	SM 32:1;O2		M+H	C37H75N2O6P	-9.203
866.6609	17.56	0.0035	0.0929	1.7	GM			16.58	PC 42:4	PC 18:3/24:1	M+H	C50H92NO8P	-2.780
892.6785	17.57	0.0006	0.0225	1.7	GM			14.23	PC 44:5	PC 20:4/24:1	M+H	C52H94NO8P	-0.487
882.5234	18.01	0.0024	0.1232	1.5	GM			12.49	PS 44:10		M-H	C50H78NO10P	-6.443
894.6914	18.15	0.0076	0.1518	1.7	GM			13.87	PC 44:4	PC 20:3/24:1	M+H, M+K	C52H96NO8P	-3.618
920.7076	18.20	0.0001	0.0053	1.7	GM			11.47	PC 46:5	PC 22:5/24:0	M+H	C54H98NO8P	-2.865
832.5144	18.22	0.0121	0.3386	1.5	Control			13.98	PS 40:7	PS 18:1/22:6	M-H	C46H76NO10P	1.222

Supplementary Table S3. SFC-QTOF analysis of eye tissue at the final time point. Simple ID was designated by SFC-QTOF, triple quad ID was determined by LC-MS/MS. Green and red represent high and low peak areas, respectively. Red triple quad IDs mean multiple acyl isomers were detected.

m/z	RT	Anova (p)	q Value	Fold	Highest	Co.	GM	CV%	Simple ID	Triple quad ID	Adducts	Formula	M.E (ppm)
554.47965	4.93	0.0001	0.0008	1.5	Control			7.65	Cer 32:1		M-H, M+Cl, M+FA-H	C32H63NO3	1.289
801.57323	6.45	0.0238	0.0495	1.4	Control			19.35	MGDG 34:1		M+FA-H	C43H80O10	-0.155
687.49714	6.94	0.0197	0.0428	1.9	Control			29.25	MGDG 34:5		M+FA-H		
716.53199	7.17	0.0000	0.0000	1.9	Control			10.38	HexCer 32:1;O2		M+FA-H	C38H73NO8	0.248
716.53207	7.35	0.0000	0.0000	2.1	Control			12.5	HexCer 32:1;O2		M-H, M+Cl, M+FA-H		
852.65718	7.53	0.0007	0.0030	1.5	GM			15.06	HexCer 42:3;O2		M+FA-H		
882.70423	7.73	0.0150	0.0339	1.4	Control			14.68	HexCer 44:2;O2		M-H, M+FA-H	C50H95NO8	0.305
819.51882	8.02	0.0013	0.0050	1.5	Control			12.75	PG 40:7		M-H		
882.70286	8.03	0.0001	0.0008	1.6	Control			11	HexCer 44:2;O2		M-H, M+FA-H	C50H95NO8	-1.322
880.65308	8.13	0.0000	0.0001	1.8	Control			6.24	HexCer 44:3;O2		M-H, M+FA-H		
908.68583	8.29	0.0509	0.0918	1.4	Control			22.74	HexCer 45:4;O3		M+FA-H		
898.6992	8.30	0.0001	0.0008	1.6	Control			13.66	HexCer 44:2;O3		M+FA-H		
924.71507	8.37	0.0003	0.0017	1.5	GM			11.21	HexCer 46:3;O3		M+FA-H		
591.39044	8.88	0.0000	0.0001	2.6	GM			18.79			Neg		
760.49213	9.02	0.0063	0.0175	1.2	GM			6.14	PE 38:7	PE 18:3/20:4	M-H	C43H72NO8P	-0.195
784.49258	9.03	0.0000	0.0000	2.7	GM			8.82	PE 40:9	PE 18:3/22:6	M-H	C45H72NO8P	0.388
810.5081	9.07	0.0000	0.0002	1.6	GM			9.23	PE 42:10	PE 20:4/22:6	M-H	C47H74NO8P	0.216
786.50817	9.10	0.0000	0.0002	1.5	GM			7.62	PE 40:8	PE 18:2/22:6	M-H	C45H74NO8P	0.311
838.53231	9.12	0.0025	0.0085	1.5	GM			12.81	PE 44:10		M-H	C49H78NO8P	-8.242
774.50814	9.13	0.0000	0.0000	2.5	Control			5.19	PE 39:7		M-H		
744.49756	9.16	0.0000	0.0001	2.0	GM			11.5	PE O-38:8		M-H		
738.50793	9.23	0.0000	0.0000	2.3	GM			6.95	PE 36:4	PC 16:0/20:4	M-H	C41H74NO8P	0.008
788.52441	9.24	0.0003	0.0013	1.3	Control			7.07	PE 40:7	PE 18:1/22:6	M-H	C45H76NO8P	1.057
856.51401	9.24	0.0000	0.0000	1.5	Control			5.49			Neg		
770.51307	9.25	0.0000	0.0000	2.5	GM			6.83	PE O-40:9	PE P-18:2/22:6	M-H		
812.52294	9.25	0.0000	0.0000	2.5	GM			10.36	PE 42:9	PE 20:3/22:6	M-H	C47H76NO8P	-0.788
814.53397	9.34	0.0000	0.0000	1.5	GM			6.62	PE 42:8	PE 20:2/22:6	M-H	C47H78NO8P	-6.447
722.51013	9.34	0.0018	0.0106	1.5	GM			15.47	PE O-36:6	PE P-16:0/20:5	M+H	C41H72NO7P	-2.471
748.52794	9.34	0.0314	0.0911	1.3	Control			12.57	PE O-38:7	PE O-18:3/20:4	M+H, M+Na	C43H74NO7P	0.494
728.52398	9.38	0.0000	0.0000	3.8	Control			6.81	PE 35:2	PE 17:1/18:1	M-H		
772.52946	9.39	0.0000	0.0000	1.4	Control			3.64	PE O-40:8	PE P-18:1/22:6	M-H	C45H76NO7P	1.028
750.53547	9.39	0.0000	0.0001	1.6	GM			3.11	PE O-38:5	PE P-18:0/20:4	M-H	C43H78NO7P	-11.769
722.51321	9.40	0.0000	0.0000	2.7	GM			8.13	PE O-36:5	PE P-16:0/20:4	M-H	C41H74NO7P	0.272
766.53875	9.48	0.0000	0.0000	2.2	GM			5.85	PE 38:4	PE 18:0/20:4	M-H	C43H78NO8P	-0.623
816.553	9.48	0.0000	0.0002	1.4	GM			5.56	PE 42:7	PE 20:1/22:6	M-H	C47H80NO8P	-2.299
742.53954	9.50	0.0075	0.0197	1.3	Control			11.06	PE 36:2	PE 18:1/18:1	M-H	C41H78NO8P	0.415
716.52397	9.53	0.0000	0.0003	1.3	Control			4.75	PE 34:1	PE 16:0/18:1	M-H	C39H76NO8P	0.544
748.52899	9.56	0.0003	0.0015	1.5	GM			11.31	PE O-38:6	PE O-18:2/20:4	M-H	C43H76NO7P	0.440
712.52878	9.59	0.0000	0.0000	2.4	Control			7.26	PE O-35:3		M-H	C40H76NO7P	0.167
792.55416	9.61	0.0002	0.0013	1.4	GM			8.36	PE 40:5	PE 18:0/22:5	M-H	C45H80NO8P	-0.902
818.56867	9.61	0.0001	0.0005	1.5	GM			9.5	PE 42:6	PE 20:1/22:5	M-H	C47H82NO8P	-2.272
796.55116	9.64	0.0000	0.0001	2.1	Control			4.55			M-H		
774.54488	9.67	0.0050	0.0145	1.3	GM			10.48	PE O-40:7	PE P-18:0/22:6	M-H	C45H78NO7P	0.726
750.5444	9.68	0.0000	0.0000	2.9	GM			12.66	PE O-38:5	PE O-18:2/20:3	M-H	C43H78NO7P	0.119
794.5491	9.71	0.0001	0.0004	1.5	GM			9.49			Neg		
728.55909	9.74	0.0016	0.0094	1.6	Control			17.04	PE O-36:3	PE P-18:0/18:2	M+H	C41H78NO7P	0.301
810.5624	9.76	0.0000	0.0000	2.4	Control			4.07	PE 40:4;O		M-H		
818.57023	9.76	0.0000	0.0000	2.5	GM			11.16	PE 42:6		M-H	C47H82NO8P	-0.361
770.5708	9.77	0.0002	0.0011	1.5	Control			9.19	PE 38:2	PE 18:1/20:1	M-H	C43H82NO8P	0.349
776.56045	9.82	0.0021	0.0075	1.5	GM			14.98	PE O-40:6	PE P-18:0/22:5	M-H	C45H80NO7P	0.626
794.57091	9.97	0.0001	0.0005	2.6	Control			5.68	PE 40:4	PE 18:0/22:4	M-H	C45H82NO8P	0.483
754.57597	10.01	0.0011	0.0044	1.5	Control			10.88	PE O-38:3		M-H	C43H82NO7P	0.467
838.59582	10.04	0.0000	0.0002	2.9	Control			10.93	PE 42:4;O		M-H		
885.55078	10.42	0.0013	0.0050	1.3	GM			8.77	PI 38:4	PI 18:0/20:4	M-H	C47H83O13P	1.045
861.54923	10.44	0.0001	0.0006	2.1	Control			16.03	PI 36:2		M-H	C45H83O13P	-0.717
911.56279	10.49	0.0023	0.0079	1.5	GM			16.43	PI 40:5		M-H	C49H85O13P	-2.976
1473.9801	12.44	0.0001	0.0006	1.8	GM			15.33	CL 74:9		M-2H, M-H	C83H144O17P2	-0.342
1449.983	12.55	0.0072	0.0191	1.4	Control			13.74	CL 72:7		M-2H, M-H	C81H144O17P2	1.633
1403.9974	13.12	0.0000	0.0002	2.2	Control			17.21	CL 68:2		M-2H, M-H	C77H146O17P2	0.803
802.53871	14.52	0.0001	0.0012	2.5	GM			20.44	PC 38:8	PC 18:3/20:5	M+H		
828.55374	14.65	0.0000	0.0003	2.5	GM			17.41	PC 40:9	PC 18:3/22:6	M+H, M+Na	C48H78NO8P	-0.052

854.56948	14.67	0.0004	0.0034	2.1	GM			19.21	PC 42:10	PC 20:5/22:5	M+H	C50H80NO8P	0.054
752.52444	14.73	0.0000	0.0001	4.5	Control			23.24	PC 34:5	PC 14:0/20:5	M+H	C42H74NO8P	2.601
778.53833	14.81	0.0005	0.0037	2.1	Control			23.4	PC 36:6	PC 18:3/18:3	M+H, M+Na	C44H76NO8P	0.256
830.57019	14.92	0.0007	0.0050	1.9	GM			17.65	PC 40:8	PC 18:3/22:5	M+H	C48H80NO8P	0.910
856.58302	14.96	0.0000	0.0002	2.7	GM			17.35	PC 42:9	PC 20:3/22:6	M+H	C50H82NO8P	-2.413
882.59672	15.03	0.0000	0.0004	2.1	GM			16.26	PC 44:10	PC 22:5/22:5	M+H	C52H84NO8P	-4.549
894.59803	15.06	0.0000	0.0000	4.6	Control			19.51	PC 45:11		M+H		
754.53695	15.10	0.0097	0.0384	1.4	Control			14.88	PC 34:4	PC 16:1/18:3	M+H	C42H76NO8P	-1.568
792.55474	15.10	0.0000	0.0003	2.1	Control			14.61	PC 37:6		M+H	C45H78NO8P	1.211
704.52158	15.18	0.0000	0.0000	5.0	Control			13.32	PC 30:1	PC 14:0/16:1	M+H, M+Na	C38H74NO8P	-1.284
678.50523	15.25	0.0000	0.0000	11.4	Control			14.9	PC 28:0	PC 14:0/14:0	M+H, M+Na	C36H72NO8P	-2.361
730.539	15.25	0.0000	0.0001	2.2	Control			13.19	PC 32:2	PC 14:0/18:2	M+H	C40H76NO8P	1.191
832.587	15.25	0.0022	0.0124	1.6	Control			18.22	PC 40:7	PC 18:1/22:6	M+H, M+Na	C48H82NO8P	2.310
522.35456	15.26	0.0009	0.0063	1.5	Control			13.98	LPC 18:1		M+H		
858.59865	15.39	0.0030	0.0158	1.5	GM			15.83	PC 42:8		M+Na, M+K	C48H86NO8P	0.391
794.56319	15.42	0.0000	0.0001	2.3	Control			12.99	PC 37:5	PC 17:0/20:5	M+H-H2O, M+K	C43H82NO7P	22.687
782.57031	15.45	0.0000	0.0002	2.0	GM			11.81	PC 36:4	PC 18:1/18:3	M+H, M+Na	C44H80NO8P	1.126
756.5548	15.50	0.0000	0.0001	2.2	GM			10.81	PC 34:3	PC 16:0/18:3	M+H, M+Na	C42H78NO8P	1.342
820.58036	15.63	0.0001	0.0009	1.8	Control			15.18	PC 39:6		M+H	C47H82NO8P	-5.755
764.55649	15.66	0.0000	0.0006	1.8	Control			7.67	PC O-36:6		M+H		
732.55521	15.71	0.0000	0.0002	1.9	Control			11.73	PC 32:1	PC 16:0/16:1	M+H, M+Na	C40H78NO8P	1.951
784.58599	15.71	0.0169	0.0586	1.3	Control			14.41	PC 36:3	PC 18:1/18:2	M+H	C44H82NO8P	1.163
714.53952	15.73	0.0000	0.0001	2.9	Control			16.41	PC O-32:3		M+H		
848.612	15.77	0.0000	0.0000	4.4	Control			14.19	PC 41:6		M+H, M+Na	C49H86NO8P	-5.174
860.61548	15.80	0.0210	0.0680	1.3	GM			16.82	PC 42:7	PC 20:1/22:6	M+H, M+Na	C50H86NO8P	-1.053
706.53746	15.81	0.0000	0.0000	3.0	Control			11.82	PC 30:0	PC 14:0/16:0	M+H, M+Na	C38H76NO8P	-0.953
792.59124	15.87	0.0000	0.0002	2.4	Control			11.14	PC O-38:6	PC P-18:0/20:5	M+H	C46H82NO7P	1.356
784.58131	15.89	0.0001	0.0004	1.3	Control			4.13			Neg		
832.5955	15.89	0.0000	0.0002	1.5	Control			4.42	PC 36:1	PC 16:0/20:1	M+FA-H		
772.58248	15.89	0.0000	0.0001	2.1	Control			11.72	PC 35:2		M+H	C43H82NO8P	-3.376
746.56994	15.97	0.0000	0.0001	2.1	Control			13.28	PC 33:1		M+H	C41H80NO8P	0.680
766.57474	16.00	0.0023	0.0126	1.5	Control			11.28	PC O-36:5	PC O-16:0/20:5	M+H	C44H80NO7P	0.297
810.60166	16.02	0.0000	0.0002	2.1	GM			12.71	PC 38:4	PC 18:0/20:4	M+H	C46H84NO8P	1.151
818.60425	16.02	0.0000	0.0000	2.7	Control			11.04	PC O-40:7	PC P-18:0/22:6	M+H, M+Na	C48H84NO7P	-1.913
836.61463	16.04	0.0055	0.0249	1.4	GM			11.4	PC 40:5	PC 18:0/22:5	M+H	C48H86NO8P	-2.093
862.63031	16.08	0.0006	0.0047	1.5	GM			12.11	PC 42:6		M+H, M+Na	C50H88NO8P	-1.997
792.59115	16.12	0.0001	0.0012	1.8	Control			12.26	PC O-38:6		M+H	C46H82NO7P	1.245
786.60215	16.13	0.0001	0.0014	1.5	Control			10.58	PC 36:2	PC 18:1/18:1	M+H	C44H84NO8P	1.800
838.62583	16.15	0.0000	0.0000	2.1	GM			10.49	PC 40:4	PC 20:0/20:4	M+H	C48H88NO8P	-7.399
760.99388	16.24	0.0055	0.0249	1.8	Control			23.29		PC 16:0/18:1	Pos		
768.58809	16.34	0.0000	0.0003	1.7	GM			10.64	PC O-36:4	PC O-16:0/20:4	M+H	C44H82NO7P	-2.709
778.56158	16.45	0.0003	0.0015	1.3	GM			6.32	PC 32:0	PC 16:0/16:0	M+FA-H	C40H80NO8P	1.659
744.58711	16.51	0.0011	0.0070	1.4	Control			7.25	PC O-34:2	PC P-18:1/16:0	M+H	C42H82NO7P	-4.115
774.59788	16.68	0.0000	0.0001	2.1	Control			11.13	PC 35:1		M+H		
718.57258	16.71	0.0000	0.0005	2.0	Control			14.12	PC O-32:1	PC O-14:0/18:1	M+H, M+Na	C40H80NO7P	-2.697
814.6273	16.89	0.0013	0.0082	1.5	Control			11.93	PC 38:2	PC 18:1/20:1	M+H		
864.64334	16.92	0.0000	0.0000	2.1	GM			11.05	PC 42:5	PC 20:0/22:5	M+H	C50H90NO8P	-5.026
737.54726	17.19	0.0002	0.0012	1.3	Control			5.82	SM 32:0;O3		M+FA-H		
772.62188	17.20	0.0016	0.0096	1.5	Control			14.35	PC O-36:2	PC P-18:0/18:1	M+H	C44H86NO7P	0.541
675.54419	17.23	0.0001	0.0009	1.9	Control			16.63	SM 32:1;O2		M+H, M+Na	C37H75N2O6P	0.940
746.60558	17.42	0.0119	0.0449	1.4	Control			17.52	PC O-34:1	PC O-16:0/18:1	M+H, M+Na	C42H84NO7P	-0.318
1020.7382	18.00	0.0044	0.0211	1.7	GM			18.06	PC 54:11		M+H		
832.51384	18.15	0.0000	0.0001	1.7	Control			10.43	PS 40:7	PS 18:1/22:6	M-H	C46H76NO10P	0.519
920.70849	18.36	0.0000	0.0000	1.8	GM			7.68	PC 46:5	PC 20:5/26:1	M+H	C54H98NO8P	-1.951
810.52616	18.89	0.0000	0.0003	1.8	GM			14.24	PS 38:4		M-H	C44H78NO10P	-3.574
786.5297	19.23	0.0001	0.0004	1.5	Control			5.95	PS 36:2		M-H	C42H78NO10P	0.820
760.51365	19.49	0.0001	0.0005	1.5	Control			9.68	PS 34:1	PS 16:0/18:1	M-H	C40H76NO10P	0.323
855.66007	19.60	0.0002	0.0011	1.3	GM			6.83	SM 42:3;O2		M+FA-H	C47H91N2O6P	0.480
898.72515	19.91	0.0001	0.0010	1.6	Control			11.57	PC 44:2	PC 22:1/22:1	M+H, M+Na	C52H100NO8P	-0.869
883.69146	20.97	0.0000	0.0000	1.6	GM			7.35	SM 44:3;O2		M+FA-H		
926.7551	21.32	0.0000	0.0002	2.5	Control			17.89	PC 46:2		M+H	C54H104NO8P	-2.305

Supplementary Table S4. SFC-QTOF analysis of eye tissue at the intermediate time point. Simple ID was designated by SFC-QTOF, triple quad ID was determined by LC-MS/MS. Green and red represent high and low peak areas, respectively. Red triple quad IDs mean multiple acyl isomers were detected.

m/z	RT	Anova (p)	q Value	Fold	Highest	Co.	GM	CV%	Simple ID	Triple quad ID	Adducts	Formula	M.E (ppm)
531.3182	6.27	1.553E-05	0.0004023	3.5	Control			21.92	MGDG 18:1		M-H	C27H48O10	1.418
773.5426	6.28	0.0072616	0.0322138	1.6	Control			19.44	MGDG 32:1		M-H, M+FA-H	C41H76O10	0.709
801.5732	6.41	0.012584	0.0473477	1.6	Control			15.33	MGDG 34:1	MGDG 16:0/18:1	M+FA-H	C43H80O10	-0.260
913.6988	7.05	0.0003491	0.0033675	2.1	Control			19.58	MGDG 42:1		M+FA-H	C51H96O10	0.307
716.5325	7.13	0.0014743	0.0098823	1.6	Control			17.73	HexCer 32:1;O2		M+FA-H	C38H73NO8	0.973
716.5324	7.29	6.282E-05	0.001009	1.9	Control			13.59	HexCer 32:1;O2		M+FA-H	C38H73NO8	0.788
852.6574	7.47	0.0006441	0.0052346	1.7	GM			17.48	HexCer 42:3;O2		M+FA-H		
880.6878	7.60	0.0001356	0.0017359	1.7	GM			13.47	HexCer 44:3;O2		M-H, M+FA-H		
882.7045	7.95	0.0024715	0.0144601	1.5	Control			15.71	HexCer 44:2;O2		M+FA-H	C50H95NO8	0.602
908.7198	8.03	0.0004783	0.004286	1.7	GM			12.31	HexCer 46:3;O2		M+FA-H		
898.6994	8.21	0.00105	0.0076548	1.6	Control			15.19	HexCer 44:2;O3		M+FA-H		
924.7154	8.29	0.0057707	0.0272535	1.4	GM			15.94	HexCer 46:3;O3		M+FA-H		
784.4929	8.91	1.514E-05	0.0003996	2.0	GM			10.16	PE 40:9	PE 18:3/22:6	M-H	C45H72NO8P	0.741
810.5083	8.96	0.0004843	0.0043054	1.9	GM			9.83	PE 42:10	PE 20:4/22:6	M-H	C47H74NO8P	0.407
786.5085	8.99	0.0029869	0.0166804	1.4	GM			12.39	PE 40:8	PE 18:2/22:6	M-H	C45H74NO8P	0.706
774.5087	9.02	1.627E-07	2.408E-05	2.9	Control			11.53	PE 39:7	PE 17:1/22:6	M-H	C44H74NO8P	0.990
838.5324	9.02	0.0006351	0.0051822	2.2	GM			16.29	PE 44:10	PE 22:5/22:5	M-H		
744.4977	9.04	1.617E-06	9.176E-05	2.1	GM			10.7	PE O-38:8	PE P-18:2/20:5	M-H		
812.5226	9.04	1.349E-05	0.0003764	2.3	GM			7.8	PE 42:9	PE 20:3/22:6	M-H		
738.5083	9.11	2.001E-08	6.812E-06	2.7	GM			9.09	PE 36:4	PE 16:0/20:4	M-H		
765.5257	9.11	0.0090207	0.0379214	1.3	GM			7.4	EPC 40:6;O4		M-H		
788.5255	9.12	0.00054	0.0046907	1.4	Control			6.96	PE 40:7	PE 18:1/22:6	M-H	C45H76NO8P	2.411
856.5117	9.12	2.953E-06	0.0001468	1.6	Control			7.51			M-H		
770.5132	9.13	1.235E-06	7.785E-05	2.6	GM			14.41	PE O-40:9	PE P-18:2/22:6	M-H		
720.4979	9.19	0.0055843	0.0265571	1.4	GM			14.42	PE O-36:6	PE P-16:0/20:5	M-H	C41H72NO7P	0.740
814.5350	9.22	0.0016425	0.0107347	1.3	GM			7.82	PE 42:8	PE 20:2/22:6	M-H	C47H78NO8P	-5.194
728.5240	9.26	2.082E-09	5.818E-06	3.9	Control			8.24	PE 35:2		M-H	C40H76NO8P	0.529
764.5233	9.26	0.0002353	0.0025244	1.5	GM			8.23	PE 38:5	PE 18:1/20:4	M-H	C43H76NO8P	-0.326
772.5299	9.27	6.631E-05	0.0010551	1.5	Control			9.7	PE O-40:8	PE P-18:1/22:6	M-H	C45H76NO7P	1.625
722.5131	9.28	5.97E-09	5.818E-06	2.9	GM			8.23	PE O-36:5	PE P-16:0/20:4	M-H	C41H74NO7P	0.177
748.5283	9.28	2.927E-07	3.115E-05	1.9	GM			7.52	PE O-38:6	PE P-18:0/20:5	M-H	C43H76NO7P	-0.435
766.5392	9.36	1.158E-08	6.571E-06	2.5	GM			4.36	PE 38:4	PE 18:0/20:4	M-H	C43H78NO8P	-0.052
742.5385	9.38	0.0093257	0.038915	1.7	Control			11.84	PE 36:2	PE 18:1/18:1	M-H	C41H78NO8P	-0.980
716.5241	9.39	0.0012051	0.0086029	1.4	Control			10.05	PE 34:1	PE 16:0/18:1	M-H	C39H76NO8P	0.792
748.5291	9.44	0.0182029	0.0615515	1.4	GM			11.92	PE O-38:6	PE P-18:1/20:4	M-H	C43H76NO7P	0.580
824.5762	9.44	0.0269203	0.0811202	2.2	Control			46.64	PE O-44:10		M-H		
712.5289	9.46	1.494E-07	2.348E-05	3.0	Control			12.94	PE O-35:3		M-H	C39H72NO8P	
768.5544	9.48	0.0014362	0.0097158	1.3	Control			7.29	PE 38:3	PE 18:1/20:2 | 18:2/20:1	M-H	C43H80NO8P	-0.649
792.5539	9.48	0.000332	0.0032673	1.7	GM			10.54	PE 40:5	PE 20:1/20:4 | 18:0/22:5	M-H	C45H80NO8P	-1.218
856.6043	9.50	0.0037753	0.0199613	2.6	Control			39.67			Neg		
796.5451	9.51	0.0001306	0.0017112	1.6	Control			11.45	PE O-42:10		M-H		
756.5396	9.53	4.541E-06	0.0001979	2.0	Control			8.59	PE 37.2		M-H, M+FA-H		
842.5446	9.54	0.0065856	0.0298594	1.2	Control			5.34	PE 44:8		M-H		
750.5437	9.54	8.317E-07	5.664E-05	2.5	GM			2.74	PE O-38:5	PE P-18:0/20:4	M-H	C43H78NO7P	-0.815
726.5463	9.59	0.0275423	0.0825561	1.4	Control			7.36	PE O-36:3	PE P-18:1/18:1	M-H	C41H78NO7P	2.726
794.5474	9.59	0.000952	0.0070936	1.4	GM			8.87	PE 40:4	PE 18:0/22:4	M-H	C45H82NO8P	-29.088
810.5611	9.63	5.892E-05	0.0009599	2.1	Control			16.42	PE 40:4;O		M-H		
818.5700	9.63	5.58E-08	1.267E-05	2.2	GM			7.71	PE 42:6	PE 20:0/22:6	M-H	C47H82NO8P	-0.692
770.5708	9.64	0.0012436	0.0087669	2.0	Control			10.88	PE 38:2	PE 18:1/20:1	M-H	C43H82NO8P	0.404
478.2935	9.73	5.624E-05	0.0009301	2.3	Control			11.22	LPE 18:1		M-H	C23H46NO7P	-0.805
794.5709	9.85	0.0004862	0.0043057	2.3	Control			20.1	PE 40:4		M-H	C45H82NO8P	0.497
754.5755	9.88	0.0091002	0.0381613	1.6	Control			9.67	PE O-38:3	PE P-18:1/20:1	M-H	C43H82NO7P	-0.187
798.5988	9.93	0.0002346	0.0025244	3.0	Control			11.06	PE 40:2	PE 20:1/20:1	M-H	C45H86NO8P	-3.745
812.5790	9.93	0.4177675	0.3487455	1.2	GM			16.86	PE 40:3;O		M-H		
855.5049	10.02	0.0072254	0.0321609	1.8	Control			21.24	PI 36:5		M-H, M+FA-H		

907.5352	10.08	0.000598	0.004966	1.8	Control			15.44	PI 40:7	PI 16:0/24:6	M-H2O-H, M-H, M+FA-H		
883.5345	10.20	0.0102791	0.0412748	1.6	Control			16.31	PI 38:5	PI 18:0/20:5	M-H	C47H81O13P	0.355
861.5496	10.28	4.936E-05	0.0008532	2.8	Control			17.75	PI 36:2	PI 18:1/18:1	M-H	C45H83O13P	-0.311
1403.9980	12.88	0.002822	0.0161225	2.1	Control			22.63	CL 68:2		M-2H, M-H	C77H146O17P2	1.216
812.5207	14.37	0.0092211	0.0385257	1.6	GM			25.99			Neg		
796.5153	14.44	1.897E-05	0.0004582	2.2	Control			16.88	PC 34:5		M+FA-H	C42H74NO8P	2.556
822.5300	14.50	0.0002358	0.0025244	1.7	Control			15.85	PC 36:6	PC 16:1/20:5	M+FA-H	C44H76NO8P	1.189
802.5381	14.60	0.0003349	0.0012863	2.2	GM			20.13	PC 38:8	PC 18:3/20:5	M+H		-0.066
874.5599	14.62	0.0015493	0.0102445	1.6	GM			16.94	PC 40:8	PC 18:3/22:5	M+FA-H	C48H80NO8P	-0.541
828.5527	14.71	0.0053264	0.0078164	1.7	GM			19.26	PC 40:9	PC 18:3/22:6	M+H, M+Na		-1.311
938.5900	14.71	3.604E-08	1.023E-05	4.1	Control			14	PC 45:11		M+FA-H		
854.5683	14.72	0.0004743	0.0016628	2.6	GM			24.15	PC 42:10	PC 20:4/22:6	M+H, M+Na		-1.346
784.4686	14.79	0.6856003	0.4568537	1.1	Control			15.06	PC 32:5;O		M+FA-H		
748.5114	14.80	9.215E-06	0.0003046	1.8	Control			8.4	PC 30:1	PC 14:0/16:1	M+FA-H		
876.5759	14.85	5.063E-05	0.000862	1.8	Control			13.15	PC 40:7	PC 20:2/20:5	M+FA-H	C48H82NO8P	-0.149
856.5816	14.98	0.0001257	0.000656	2.5	GM			20.4	PC 42:9	PC 20:4/22:5	M+H		-4.107
852.5735	15.00	0.0005644	0.0048335	1.4	GM			10.53	PC 38:5	PC 16:0/22:5	M+FA-H	C46H82NO8P	-3.145
882.5928	15.05	0.0004631	0.0016431	2.3	GM			20.74	PC 44:10		M+H		-8.947
766.5378	15.10	6.783E-09	5.818E-06	2.2	GM			4.59		PS P-16:0/20:4	Neg		
894.5527	15.12	9.602E-07	6.411E-05	1.8	GM			5.59	PC O-43:12		M+FA-H		
800.5450	15.17	0.0001835	0.002154	1.5	GM			10.78	PC 34:3	PC 16:0/18:3	M-H, M+FA-H	C42H78NO8P	0.452
776.5459	15.34	3.528E-05	0.0006788	1.6	Control			10.84	PC 32:1	PC 16:0/16:1	M+FA-H	C40H78NO8P	1.610
938.5393	15.40	0.0391338	0.1050068	1.4	GM			20.34	PC 44:12;O		M+FA-H		
690.5079	15.44	4.538E-07	4.067E-05	2.3	Control			10.14			Neg		
750.5299	15.45	1.331E-05	0.0003745	1.7	Control			10.86	PC 30:0	PC 14:0/16:0	M+FA-H	C38H76NO8P	1.154
836.5812	15.48	6.209E-05	0.001002	1.4	Control			7.57	PC O-38:6	PC P-18:0/20:5	M+FA-H	C46H82NO7P	0.183
782.5694	15.52	8.931E-06	0.0001243	2.1	GM			10.49	PC 36:4	PC 18:2/18:2	M+H, M+Na		-0.076
790.5600	15.59	0.0013541	0.0092898	1.6	Control			18.14	PC 33:1		M+FA-H	C41H80NO8P	-0.502
862.5967	15.64	5.305E-08	1.267E-05	2.9	Control			8.71	PC O-40:7	PC P-18:0/22:6	M+FA-H	C48H84NO7P	-0.066
830.5916	15.71	0.0004345	0.0040093	1.6	Control			5.57	PC 36:2	PC 18:1/18:1	M+FA-H	C44H84NO8P	-0.085
744.5553	15.83	0.0064584	0.0294791	1.3	Control			9.71			Neg		
848.6128	15.85	2.055E-07	1.655E-05	6.4	Control			17.19	PC 41:6		M+H, M+Na		-4.249
758.5697	15.87	3.284E-05	0.000654	1.5	Control			9.42			Neg		
810.5993	16.09	0.0001851	0.0008491	1.9	GM			13.65	PC 38:4	PC 18:0/20:4	M+H		-1.794
818.5913	16.34	5.036E-05	0.000862	1.9	Control			13.17	PC 35:1	PC 17:0/18:1	M+FA-H	C44H86NO10P	-0.379
768.5871	16.38	6.692E-07	2.57E-05	1.8	GM			3.92	PC O-36:4	PC O-16:0/20:4	M+H		-3.982
762.5656	16.45	0.000373	0.0035182	1.9	Control			9.81	PC O-32:1		M+FA-H	C40H80NO7P	0.241
858.6231	16.61	0.0012693	0.0088916	1.8	Control			9.9	PC 38:2	PC 18:1/20:1	M+FA-H	C46H88NO8P	0.131
838.6283	16.69	6.021E-06	9.498E-05	2.4	GM			12.86	PC 40:4	PC 20:3/20:1	M+H		-4.411
900.5970	16.77	0.2525255	0.262615	1.1	Control			8.41			Neg		
846.5985	16.80	5.022E-06	0.000206	1.9	Control			6.68			Neg		
816.6122	16.91	0.0209228	0.0680495	1.5	Control			12.76	PC O-36:2	PC P-18:0/18:1	M+FA-H	C44H86NO7P	-0.283
659.5139	16.94	0.000828	0.0063358	1.7	Control			14.15			Neg		
719.5351	16.95	0.0015494	0.0102445	1.6	Control			15.74	SM 32:1		M+FA-H	C37H75N2O6P	0.902
790.5969	17.20	0.0155505	0.0551571	1.5	Control			11.39	PC O-34:1	PC O-16:0/18:1	M+FA-H	C42H84NO7P	0.252
892.6758	17.50	0.0602643	0.0361679	1.7	GM			20.91	PC 44:5	PC 20:4/24:1	M+H		-3.514
886.6541	17.54	0.0041562	0.0214104	2.3	Control			19.15	PC 40:2	PC 40:2	M+FA-H	C48H92NO8P	-0.240
832.5140	17.78	3.83E-06	0.0001787	2.3	Control			10.41	PS 40:7	PS 18:1/22:6	M-H	C46H76NO10P	0.727
806.4984	17.89	0.0067744	0.0305931	1.4	Control			12.76	PS 38:6	PS 16:0/22:6	M-H	C44H74NO10P	0.832
786.5298	18.96	4.527E-05	0.0008023	1.6	Control			10.19	PS 36:2	PS 18:1/18:1	M-H	C42H78NO10P	0.896
760.5138	19.07	0.0057215	0.0270586	1.6	Control			17.93	PS 34:1	PS 16:0/18:1	M-H	C40H76NO10P	0.515
855.6602	19.34	0.0021487	0.0129497	1.4	GM			11.93	SM 42:3;O2		M+FA-H	C47H91N2O6P	0.682
922.7110	19.37	8.205E-05	0.000545	1.3	GM			5.4	PC O-44:2		M+H, M+K		9.549
942.7144	19.84	0.0003218	0.0031947	1.8	Control			6.76	PC 44:2		M+FA-H	C52H100NO8P	-2.714
814.5592	20.53	7.798E-05	0.0012125	1.9	Control			15.98	PS 38:2	PS 18:1/20:1	M-H	C43H80NO8P	-1.558
883.6917	20.62	0.000104	0.0014788	1.7	GM			13.41	SM 44:3;O2		M+FA-H		
788.5447	20.86	0.0240876	0.0755251	1.3	Control			12.2	PS 36:1	PS 18:0/18:1	M-H	C42H80NO10P	-0.068
970.7462	21.31	5.118E-05	0.000867	2.5	Control			16.1	PC 46:2		M+FA-H		

Supplementary Table S5. SFC-QTOF analysis of gill tissue at the final time point. Simple ID was designated by SFC-QTOF, triple quad ID was determined by LC-MS/MS. Green and red represent high and low peak areas, respectively. Red triple quad IDs mean multiple acyl isomers were detected.

m/z	RT	Anova (p)	q Value	Fold	Highest	Co.	GM	CV%	Simple ID	Triple Quad ID	Adducts	Formula	M.E. (ppm)
554.4801	4.95	4.087E-05	0.000207	1.8	Control			6.52	Cer 32:1;O2		M-H, M+Cl, M+FA-H	C32H63NO3	2.085
556.4900	4.95	0.0006184	0.000958	1.6	Control			13.05	Cer 32:0;O2		M+FA-H	C32H65NO3	-8.990
572.4907	5.24	0.0420679	0.019971	1.3	Control			18.14			M-H, M+FA-H		
664.5894	5.35	0.0258846	0.013532	1.3	GM			16.76	Cer 40:2;O2		M-H, M+FA-H	C40H77NO3	1.348
702.6048	5.52	0.0117669	0.007743	1.6	Control			10.57	Cer 43:4;O2		M-H, M+FA-H		
708.6157	5.69	0.0001987	0.000478	2.0	GM			16.6	Cer 42:2;O3		M-H, M+FA-H	C42H81NO4	1.353
854.6725	7.86	0.0002019	0.000478	1.8	Control			11.6	HexCer 42:2;O2		M-H, M+FA-H	C48H91NO8	-0.237
767.4874	7.87	0.0435584	0.020522	2.1	Control			7.78	PG 36:5	PG 16:0/20:5	M-H	C42H73O10P	0.711
793.5035	7.96	0.0585066	0.0259	1.9	Control			14.25	PG 38:6	PG 16:0/22:6	M-H	C44H75O10P	1.274
819.5186	7.97	0.0003389	0.000664	4.2	Control			11.35	PG 40:7	PG 18:1/22:6	M-H	C46H77O10P	0.565
747.5192	8.14	0.000117	0.000399	2.3	Control			13.98	PG 34:1	PG 16:0/18:1	M-H	C40H77O10P	1.403
773.5323	8.14	0.0038271	0.003451	2.1	Control			10.3	PG 36:2	PG 18:1/18:1	M-H	C42H79O10P	-1.969
888.6250	8.56	0.00291	0.002902	1.5	Control			10.19	SHexCer 42:2;O2		M-H		
736.4921	9.10	0.003716	0.003405	4.1	Control			29.22	PE 36:5	PE 16:0/20:5	M-H	C41H72NO8P	-0.259
762.5088	9.19	0.005041	0.004196	2.6	Control			28.96	PE 38:6	PE 18:1/20:5	M-H	C43H74NO8P	1.169
788.5245	9.19	7.393E-05	0.0003	5.6	Control			24.08	PE 40:7	PE 18:1/22:6	M-H	C45H76NO8P	1.149
746.5134	9.25	0.0040638	0.003593	3.6	Control			26.06	PE O-38:7	PE P-18:1/20:5	M-H	C43H74NO7P	0.559
764.5241	9.33	0.0077844	0.005655	2.9	Control			28.75	PE 38:5	PE 16:0/22:5	M-H	C43H76NO8P	0.646
772.5296	9.34	0.0002049	0.000478	4.8	Control			25.69	PE O-40:8	PE P-18:1/22:6	M-H	C45H76NO7P	1.250
790.5402	9.44	0.0109013	0.007383	2.3	Control			28.35	PE 40:6	PE 18:0/22:6	M-H	C45H78NO8P	1.202
742.5381	9.45	4.991E-06	5.06E-05	8.8	Control			21.39	PE 36:2	PE 18:1/18:1	M-H	C41H78NO8P	-1.522
774.5426	9.46	0.0012604	0.001615	5.2	Control			24.29	PE O-40:7	PE P-18:1/22:5	M-H	C45H78NO7P	-2.171
716.5231	9.47	0.0002587	0.000528	4.5	Control			29.82	PE 34:1	PE 16:0/18:1	M-H	C39H76NO8P	-0.713
726.5444	9.65	1.212E-06	2.81E-05	13.7	Control			16.23	PE O-36:3	PE P-18:1/18:1	M-H	C41H78NO7P	0.150
802.5392	14.14	0.0079223	0.043711	1.8	GM			24.72	PC 38:8	PC 18:3/20:5	M+H	C46H76NO8P	1.291
828.5497	14.26	0.0016791	0.016747	1.8	GM			17.01	PC 40:9	PC 18:3/22:6	M+H, M+K	C48H78NO8P	-4.988
752.5211	14.29	0.0001563	0.003801	2.6	Control			18.65	PC 34:5	PC 14:0/20:5	M+H	C42H74NO8P	-1.902
778.5391	14.42	0.002813	0.022539	1.7	Control			16.21	PC 36:6	PC 16:1/20:5	M+H	C44H76NO8P	1.260
830.5691	14.47	0.009026	0.047242	1.6	GM			23.32	PC 40:8	PC 18:2/22:6	M+H	C48H80NO8P	-0.400
766.5346	14.49	0.0012253	0.013707	1.8	Control			13.07	PC 35:5		M+H	C43H76NO8P	-4.571
894.5972	14.62	0.0001681	0.003962	2.6	Control			16.97	PC 45:11		M+H, M+Na	C52H96NO8P	-3.912
730.5382	14.67	0.0111952	0.053006	1.4	Control			12.36	PC 32:2	PC 16:1/16:1	M+H-H2O, M+H, M+K	C40H76NO8P	0.160
704.5235	14.70	1.084E-06	0.000134	3.3	Control			13.45	PC 30:1	PC 14:0/16:1	M+H, M+Na	C38H74NO8P	1.413
678.5030	14.77	2.672E-08	9.53E-06	6.9	Control			15.64	PC 28:0	PC 14:0/14:0	M+H, M+Na	C36H72NO8P	-5.710
832.5860	14.78	0.010224	0.049938	1.5	Control			17.89	PC 40:7	PC 18:1/22:6	M+H, M+Na	C48H82NO8P	1.065
844.5523	14.85	0.0089849	0.047199	1.5	Control			13.15	PC 40:9;O		M+H		
738.5224	14.90	0.0012511	0.013924	1.6	Control			14.1			Pos		
858.5961	14.93	0.029971	0.091774	1.4	GM			16.36	PC 42:8		M+H	C48H86NO8P	-2.606
794.5547	14.93	0.000821	0.010742	1.8	Control			13.28	PC 37:5		M+H	C46H84NO7P	-18.500
718.5369	14.95	0.0005108	0.007914	1.5	Control			10.95	PC 31:1		M+H	C39H76NO8P	-1.726
782.5702	14.95	0.005048	0.033643	1.6	GM			12.41	PC 36:4	PC 18:1/18:3	M+H, M+Na	C44H80NO8P	0.972
756.5538	14.98	0.0001921	0.004182	2.0	GM			18.22	PC 34:3	PC 16:0/18:3	M+H	C42H78NO8P	-0.017
692.5214	15.02	5.461E-08	1.66E-05	4.4	Control			13.2	PC 29:0		M+H	C37H74NO8P	-1.614
764.5556	15.03	3.285E-05	0.001442	2.0	Control			11.82	PC O-36:6		M+H	C44H78NO7P	-4.269
820.5821	15.12	9.782E-05	0.002937	1.9	Control			15.4	PC 39:6		M+H	C47H82NO8P	-3.657
824.5454	15.14	0.0130583	0.008243	1.6	Control			7.66	PC 36:5	PC 16:0/20:5	M+FA-H	C44H78NO8P	0.871
732.5542	15.19	8.608E-05	0.002728	1.9	Control			11.93	PC 32:1	PC 16:0/16:1	M+H-H2O, M+H, M+Na	C40H78NO8P	0.548
496.3403	15.24	0.0035241	0.026315	1.4	GM			12.76	LPC 16:0		M+H-H2O, M+H, M+Na	C24H50NO7P	1.022
848.6092	15.26	3.79E-05	0.00159	2.4	Control			12.21	PC 41:6		M+H	C49H86NO8P	-8.447
850.5611	15.29	0.0061219	0.004743	1.5	Control			8.93	PC 38:6	PC 18:1/20:5	M+FA-H	C46H80NO8P	0.889
706.5383	15.29	4.011E-07	5.96E-05	3.2	Control			10.98	PC 30:0	PC 14:0/16:0	M+H, M+Na	C38H76NO8P	0.187
772.5813	15.36	6.067E-05	0.002166	1.9	Control			13.62	PC 35:2		M+H	C43H82NO8P	-4.957
746.5692	15.44	0.0001802	0.004078	1.7	Control			13.93	PC 33:1		M+H	C41H80NO8P	-0.295
766.5740	15.47	0.0244733	0.081381	1.4	Control			17.88	PC O-36:5		M+H	C44H80NO7P	-0.722
810.5996	15.49	0.0058199	0.036448	1.5	GM			13.9	PC 38:4	PC 18:1/20:3	M+H	C46H84NO8P	-1.437
818.6034	15.50	0.0001893	0.004168	2.3	Control			20.19	PC O-40:7		M+H, M+Na	C48H84NO7P	-2.992
716.5506	15.52	7.04E-06	0.000515	1.9	Control			9.38	PC O-32:2		M+H	C40H78NO7P	-11.566
720.5526	15.55	0.0003808	0.006487	1.5	Control			10.29	PC 31:0		M+H	C39H78NO8P	-1.625
690.5378	15.58	8.836E-09	6.07E-06	3.9	Control			4.18	PC O-30:1		M+H, M+Na	C38H76NO7P	-7.827
786.6015	15.59	0.0002515	0.004962	1.9	Control			14.74	PC 36:2	PC 18:1/18:1	M+H	C44H84NO8P	0.980
550.3855	15.59	0.000797	0.010574	1.6	GM			7.78	LPC 20:1		M+H, M+Na	C28H56NO7P	-2.219
792.5889	15.59	0.0026765	0.021876	1.8	Control			18.88	PC O-38:6	PC P-18:0/20:5	M+H	C46H82NO7P	-1.618
774.5986	16.05	0.0001899	0.004168	1.7	Control			13.22	PC 35:1	PC 18:0/17:1|18:1/17:0	M+H	C43H84NO8P	-2.716
718.5722	16.09	1.058E-05	0.00069	2.2	Control			14.19	PC O-32:1		M+H	C40H80NO7P	-3.271
804.5767	16.21	0.0002243	0.000484	1.6	Control			12.72	PC 34:1	PC 16:0/18:1	M+FA-H	C42H82NO8P	0.922
872.5626	16.22	0.0001328	0.000412	1.7	Control			14.23			Neg		
552.4004	16.25	7.099E-07	9.64E-05	2.7	GM			9.18	LPC 20:0		M+H	C28H58NO7P	-3.585
508.3750	16.31	0.0003485	0.006175	1.5	Control			10.04	LPC O-18:1		M+H, M+Na	C26H54NO6P	-2.220
772.6197	16.54	1.699E-06	0.000186	2.9	Control			14.68	PC O-36:2	PC P-18:0/18:1	M+H	C44H86NO7P	-2.311
675.5426	16.61	0.0001277	0.003437	1.7	Control			6.93	SM 32:1;O2		M+H, M+Na	C37H75N2O6P	-1.338
746.6042	16.75	9.95E-05	0.002942	1.8	Control			10.19	PC O-34:1	PC P-18:0/16:0	M+H	C42H84NO7P	-2.205
832.6078	17.13	0.0005312	0.000846	1.5	Control			10.2	PC 36:1	PC 18:0/18:1	M+FA-H	C44H86NO8P	0.643
870.6938	17.88	0.0074619	0.041948	1.7	Control			20.57	PC 42:2		M+H, M+Na	C50H96NO8P	-0.945
811.6676	18.65	0.0042618	0.029658	1.3	GM			8.93	SM 42:3;O2		M+H-H2O, M+H	C47H91N2O6P	-1.477
806.4938	18.81	0.0125603	0.008048	2.1	Control			28.68	PS 38:6		M-H	C44H74NO10P	-4.933
834.5268	19.55	0.0134566	0.008429	2.0	Control			27.14	PS 40:6	PS 18:0/22:6	M-H	C46H78NO10P	-2.743

Supplementary Table S6. SFC-QTOF analysis of gill tissue at the intermediate time point. Simple ID was designated by SFC-QTOF, triple quad ID was determined by LC-MS/MS. Green and red represent high and low peak areas, respectively. Red triple quad IDs mean multiple acyl isomers were detected.

m/z	RT	Anova (p)	q Value	Fold	Highest	Co.	GM	CV%	Simple ID	Triple Quad ID	Adducts	Formula	M.E. (ppm)
644.5982	5.70	6.39E-05	0.000121	1.7	GM			9.05	Cer 42:3;O2		M-H, M+Cl		
690.6029	5.70	7.28E-06	2.27E-05	1.8	GM			11.67	Cer 44:4;O		M-H, M+Cl, M+FA-H		
708.6142	5.98	1.69E-08	2.43E-07	2.7	GM			6.41	Cer 42:2;O3		M-H, M+FA-H		
829.6971	6.54	6.99E-08	4.79E-07	2.8	GM			11.33			Pos		
888.6236	8.73	0.002376	0.002417	1.4	Control			12.71			Neg		
591.3906	9.09	3.46E-07	2.42E-06	4.4	GM			17.09			Neg		
774.5071	9.51	5.68E-07	3.61E-06	7.1	Control			18.6	PE 39:7		M-H		
788.5234	9.63	8.32E-07	4.35E-06	4.9	Control			16.27	PE 40:7	PE 18:1/22:6	M-H	C45H76NO8P	-0.179
762.5080	9.65	0.00014	0.000225	2.4	Control			16.15	PE 38:6	PE 16:0/22:6	M-H	C43H74NO8P	0.083
746.5129	9.70	0.00096	0.001105	2.1	Control			10.47	PE O-38:7	PE P-18:1/20:5	M-H	C43H74NO7P	-0.155
740.5223	9.77	5.99E-06	2.05E-05	3.5	Control			10.06	PE 36:3		M-H	C41H76NO8P	-1.778
772.5288	9.80	3.25E-06	1.21E-05	4.5	Control			13.56	PE O-40:8	PE P-18:1/22:6	M-H	C45H76NO7P	0.147
816.5520	9.89	0.000255	0.000371	2.4	Control			14.71	PE 42:7	PE 20:1/22:6	M-H	C47H80NO8P	-3.526
790.5395	9.91	0.004639	0.004387	1.7	Control			15.83	PE 40:6	PE 18:0/22:6	M-H	C45H78NO8P	0.348
742.5388	9.94	9.74E-07	4.59E-06	5.9	Control			11.33	PE 36:2	PE 18:1/18:1	M-H	C41H78NO8P	-0.603
712.5280	10.02	6.96E-07	4.06E-06	15.2	Control			17.28	PE O-35:3	PE P-18:1/17:1	M-H		
774.5447	10.08	0.010838	0.009312	1.6	Control			17.02	PE O-40:7	PE P-18:0/22:6	M-H	C45H78NO7P	0.544
726.5442	10.16	8.75E-07	4.39E-06	7.3	Control			12.07	PE O-36:3	PE P-18:0/18:2|18:1/18:1	M-H	C41H78NO7P	-0.123
881.5168	10.53	0.014036	0.011597	1.7	Control			22.85	PI 38:6		M-H	C47H79O13P	-1.975
883.5322	10.65	0.000332	0.000447	2.3	Control			17.94	PI 38:5		M-H	C47H81O13P	-2.277
745.4791	12.09	0.00014	0.000225	2.2	Control			18.06			Neg		
828.5494	14.37	7.84E-06	1.38E-05	1.7	GM			7.64	PC 40:9		M+H	C48H78NO8P	-5.243
854.5622	14.38	1.36E-05	2.17E-05	1.4	GM			7.28	PC 42:10		M+H, M+Na	C50H80NO8P	-8.452
778.5391	14.60	2.13E-05	3.18E-05	1.6	Control			10.2	PC 36:6	PC 16:1/20:5	M+H	C44H76NO8P	1.229
830.5662	14.65	0.00145	0.001105	1.4	GM			11.5	PC 40:8	PC 18:2/22:6	M+H	C48H80NO8P	-3.939
832.5174	14.76	9.55E-09	1.66E-07	2.3	Control			5.81	PC 37:8		M+FA-H		
806.4982	14.79	8.58E-06	2.61E-05	2.0	Control			13.21	PC 35:7		M+FA-H		
522.3557	14.90	2.46E-06	5.79E-06	1.8	Control			8.14	PC 18:1		M+H, M+Na	C26H52NO7P	0.493
704.5207	14.93	3.03E-08	2.82E-07	2.4	Control			9.74	PC 30:1	PC 14:0/16:1	M+H, M+Na, M+K	C38H74NO8P	-2.532
730.5392	14.98	2.76E-05	3.92E-05	1.4	Control			4.99	PC 32:2	PC 14:0/18:2	M+H, M+K	C40H76NO8P	1.409
832.5847	14.98	3.99E-06	8.24E-06	2.0	Control			11.36	PC 40:7	PC 18:1/22:6	M+H, M+Na	C48H82NO8P	-0.502
496.3400	14.99	0.005373	0.003538	1.4	GM			13.32	PC 16:0		M+H-H2O, M+H, M+Na	C24H50NO7P	0.402
782.5688	15.22	5.45E-07	2.04E-06	1.8	GM			7.38	PC 36:4	PC 18:2/18:2	M+H, M+Na	C44H80NO8P	-0.823
834.5311	15.34	0.000218	0.000335	1.5	Control			10.59	PC 37:7		M+FA-H	C45H76NO8P	2.564
848.6145	15.45	2.13E-08	2.16E-07	3.8	Control			6.59	PC 41:6		M+H	C49H86NO8P	-2.233
784.5838	15.46	0.00094	0.000774	1.3	Control			4.19	PC 36:3	PC 18:1/18:2	M+H	C44H82NO8P	-1.688
732.5545	15.47	2.81E-06	6.29E-06	1.8	Control			9.63	PC 32:1	PC 16:0/16:1	M+H, M+Na	C40H78NO8P	0.940
758.5700	15.58	0.000977	0.000799	1.3	Control			5.8	PC 34:2	PC 16:1/18:1	M+H	C42H80NO8P	0.716
706.5381	15.61	1.56E-08	1.74E-07	2.7	Control			10.18	PC 30:0	PC 14:0/16:0	M+H, M+Na	C38H76NO8P	-0.100
792.5898	15.61	5.85E-06	1.1E-05	2.7	Control			9.95	PC O-38:6	PC P-18:0/20:5	M+H	C46H82NO7P	-0.438
786.5258	15.64	9.8E-07	4.59E-06	2.2	Control			5.61	PC 33:3		M+FA-H	C41H76NO8P	-4.456
772.5843	15.64	5.33E-10	3.11E-08	3.1	Control			4.85	PC 35:2		M+H	C43H82NO8P	-1.035
818.6055	15.76	3.57E-08	2.87E-07	3.8	Control			12.16	PC O-40:7	PC P-18:0/22:6	M+H, M+Na	C48H84NO7P	-0.379
746.5676	15.77	2.14E-06	5.26E-06	1.9	Control			11.23	PC 33:1	PC 16:0/17:1	M+H	C41H80NO8P	-2.418
810.5999	15.83	4.07E-07	1.64E-06	2.0	GM			7.91	PC 38:4	PC 16:0/22:4	M+H	C46H84NO8P	-1.037
850.5577	15.87	0.00069	0.000819	1.6	Control			10.91	PC 38:6	PC 18:1/20:5	M+FA-H	C46H80NO8P	-3.250
792.5899	15.87	0.001527	0.001156	1.5	Control			12.16	PC O-38:6		M+H	C46H82NO7P	-0.292
786.6008	15.93	5.31E-08	3.87E-07	2.3	Control			6.1	PC 36:2	PC 18:1/18:1	M+H	C44H84NO8P	0.106
782.5670	16.05	0.00103	0.000834	1.4	Control			7.98	PC 36:4		M+H	C44H80NO8P	-3.069
850.5603	16.12	8.8E-05	0.000154	1.4	Control			8.18	PC 38:6		M+FA-H	C46H80NO8P	-0.017
788.5489	16.40	2.15E-05	5.32E-05	1.6	Control			3.76	PC 33:2		M+FA-H	C41H78NO8P	5.594
718.5735	16.43	1.57E-06	4.29E-06	2.4	Control			14.54	PC O-32:1		M+H		
774.5999	16.47	1.37E-07	7.99E-07	2.4	Control			8.3	PC 35:1		M+H		
804.5644	16.70	2.13E-05	5.32E-05	1.6	Control			8.25	PC 34:1	PC 16:0/18:1	M+FA-H		
814.6316	16.71	1.81E-08	1.92E-07	2.5	Control			6.32	PC 38:2	PC 18:1/20:1	M+H	C46H88NO8P	-0.511
864.5826	16.86	6.92E-05	0.00013	1.4	Control			9.01	PC 39:6		M+FA-H	C47H82NO8P	8.015
788.6165	16.87	0.006446	0.004151	1.3	Control			9.31	PC 36:1	PC 16:0/20:1|18:0/18:1	M+H	C44H86NO8P	0.128
772.6211	16.99	3.93E-10	3.11E-08	5.9	Control			8.69	PC O-36:2	PC P-18:0/18:1	M+H	C44H86NO7P	-0.450
856.6055	17.22	8.39E-07	4.35E-06	1.5	Control			5.04	PC 38:3	PC 18:1/20:2	M+FA-H	C46H86NO8P	-2.194
746.6058	17.23	0.0001	0.000114	2.0	Control			17.26	PC O-34:1	PC O-16:0/18:1	M+H	C42H84NO7P	0.009
872.5632	17.24	7.57E-07	4.29E-06	1.5	Control			4.85			Neg		
804.5763	17.25	1.06E-05	3.01E-05	1.5	Control			7.49	PC 34:1		M+FA-H	C42H82NO8P	0.367
762.5401	17.73	3.42E-08	4.34E-07	2.4	Control			7.39	PS 34:0		M-H		

Supplementary Table S7. SFC-QTOF analysis of intestine tissue at the final time point. Simple ID was designated by SFC-QTOF, triple quad ID was determined by LC-MS/MS. Green and red represent high and low peak areas, respectively. Red triple quad IDs mean multiple acyl isomers were detected.

m/z	RT	Anova (p)	q Value	Fold	Highest	Co.	GM	CV%	Simple ID	Triple Quad ID	Adducts	Formula	M.E. (ppm)
554.4796	4.94	0.0048909	0.003356	1.8	Control			15.26	Cer 32:1;O2		M-H, M+Cl, M+FA-H	C32H63NO3	1.082
765.4713	7.73	7.354E-06	4.2E-05	6.7	Control			21.53	PG 36:6	PG 14:0/22:6	M-H	C42H71O10P	0.116
791.4879	7.76	0.0154217	0.007217	1.7	Control			19.16	PG 38:7	PG 18:2/20:5	M-H	C44H73O10P	1.266
767.4879	7.84	0.0085835	0.004674	1.9	Control			21.84	PG 36:5	PG 16:0/20:5	M-H	C42H73O10P	1.402
793.5035	7.93	0.0047672	0.00334	2.0	Control			25.46	PG 38:6	PG 18:1/20:5	M-H	C44H75O10P	1.229
819.5189	7.93	5.35E-05	0.000178	3.4	Control			27.15	PG 40:7	PG 18:1/22:6	M-H	C46H77O10P	0.945
771.5183	7.98	0.0020642	0.002	2.7	Control			34.1	PG 36:3		M-H	C42H77O10P	0.192
795.5168	8.01	0.0345489	0.014002	1.6	Control			26.54	PG 38:5		M-H	C44H77O10P	-1.749
821.5340	8.01	0.0009197	0.001262	2.5	Control			22.91	PG 40:6	PG 18:0/22:6	M-H	C46H79O10P	0.226
773.5328	8.08	1.98E-07	4.39E-06	16.3	Control			37.54	PG 36:2	PG 16:0/20:2	M-H	C42H79O10P	-1.286
747.5191	8.08	0.000516	0.000881	3.1	Control			33.06	PG 34:1	PG 16:0/18:1	M-H	C40H77O10P	1.238
734.4759	8.90	4.093E-07	6.41E-06	13.5	Control			18.79	PE 36:6	PE 16:1/20:5	M-H	C41H70NO8P	-0.986
760.4929	8.91	0.0170735	0.007784	1.7	Control			16.17	PE 38:7	PE 18:2/20:5	M-H	C43H72NO8P	0.772
784.4932	8.92	0.0068106	0.004146	2.3	GM			12.86	PE 40:9	PE 18:3/22:6	M-H	C45H72NO8P	1.140
792.5531	9.02	0.0001567	0.000805	2.2	Control			21.56	PE 40:6	PE 18:1/22:5	M+H, M+K	C45H78NO8P	-0.837
736.4928	9.03	0.0013333	0.001524	2.2	Control			11.65	PE 36:5	PE 16:0/20:5	M-H	C41H72NO8P	0.657
738.5082	9.11	0.006645	0.004096	2.1	GM			11.6	PE 36:4	PE 16:0/20:4	M-H	C41H74NO8P	0.334
788.5240	9.12	7.176E-05	0.000217	2.8	Control			12.95	PE 40:7	PE 20:2/20:5	M-H	C45H76NO8P	0.514
768.5529	9.12	0.0001488	0.000784	2.2	GM			8.6	PE 38:4	PE 16:0/22:4	M+H	C43H78NO8P	-1.109
762.5086	9.13	0.0020924	0.002	1.9	Control			10.13	PE 38:6	PE 18:1/20:5	M-H	C43H74NO8P	0.852
830.4959	9.13	0.0056096	0.00367	1.8	Control			10.83			Neg		
856.5112	9.13	1.274E-05	6.17E-05	3.8	Control			13.94			Neg		
812.5240	9.14	0.0002195	0.000483	3.4	GM			8.76	PE 42:9	PE 20:3/22:6	M-H	C47H76NO8P	0.526
746.5133	9.18	0.00069	0.00105	3.0	Control			16.3	PE O-38:7	PE P-18:1/20:5	M-H	C43H74NO7P	0.446
740.5233	9.23	0.0001406	0.000367	3.2	Control			20.71	PE 36:3	PE 18:1/18:2	M-H	C41H76NO8P	-0.416
776.5255	9.25	0.0001609	0.000393	2.6	Control			11.06	PE 39:6	PE 17:0/22:6	M-H	C44H76NO8P	2.501
764.5228	9.26	0.0062449	0.00395	1.7	Control			10.96	PE 38:5	PE 16:0/22:5	M-H	C43H76NO8P	-0.957
772.5293	9.28	0.000685	0.00105	2.6	Control			14.55	PE O-40:8	PE P-18:1/22:6	M-H	C45H76NO7P	0.862
742.5385	9.38	4.37E-06	3.32E-05	6.9	Control			21.64	PE 36:2	PE 18:1/18:1	M-H	C41H78NO8P	-0.968
794.5684	9.61	0.0011838	0.001458	2.6	GM			14.24	PE 40:4	PE 18:0/22:4	M-H	C45H82NO8P	-2.712
818.5711	9.63	0.0001114	0.000306	3.5	GM			16.6	PE 42:6	PE 20:1/22:5	M-H	C47H82NO8P	0.744
820.5857	9.75	6.716E-05	0.000208	4.0	GM			19.25	PE 42:5	PE 20:1/22:4	M-H	C47H84NO8P	-0.637
802.5381	13.65	0.008147	0.013628	2.5	GM			35.81	PC 38:8	PC 18:3/20:5	M+H, M+Na	C46H76NO8P	-0.031
878.5686	13.70	0.0193181	0.025286	1.8	GM			25.24	PC 44:12	PC 22:6/22:6	M+H, M+Na	C52H80NO8P	-0.901
828.5536	13.76	0.0002499	0.001118	2.9	GM			29.12	PC 40:9	PC 20:4/20:5	M+H, M+Na	C48H78NO8P	-0.175
752.5220	13.77	4.205E-06	6.92E-05	6.4	Control			28.05	PC 34:5	PC 14:0/20:5	M+H, M+Na	C42H74NO8P	-0.671
854.5678	13.77	0.0019201	0.005018	1.9	GM			23.32	PC 42:10	PC 20:5/22:5	M+H, M+Na	C50H80NO8P	-1.941
778.5380	13.81	0.0056277	0.010647	2.0	Control			25.84	PC 36:6	PC 18:3/18:3	M+H, M+Na	C44H76NO8P	-0.107
880.5838	13.88	0.0157712	0.021566	1.5	GM			20.1	PC 44:11	PC 22:5/22:6	M+H, M+Na	C52H82NO8P	-1.490
830.5690	13.95	0.0023	0.005688	2.0	GM			25.57	PC 40:8	PC 18:3/22:5	M+H, M+Na	C48H80NO8P	-0.504
766.5371	13.96	7.228E-05	0.000468	2.5	Control			18.35	PC 35:5		M+H	C43H76NO8P	-1.386
728.5213	13.97	9.071E-05	0.000551	4.5	Control			24.67	PC 32:3	PC 14:0/18:3	M+H	C40H74NO8P	-1.672
856.5833	14.00	1.704E-05	0.000167	2.4	GM			12.67	PC 42:9	PC 20:4/22:5	M+H	C50H82NO8P	-2.044
754.5373	14.05	0.0006889	0.002438	2.3	Control			16.23	PC 34:4	PC 16:1/18:3	M+H	C42H76NO8P	-1.108
792.5530	14.05	0.0001801	0.000887	1.9	Control			14.73	PC 37:6	PC 15:0/22:6	M+H	C45H78NO8P	-1.003
704.5209	14.07	3.367E-09	6E-07	12.6	Control			13.24	PC 30:1	PC 14:0/16:1	M+H	C38H74NO8P	-2.180
730.5368	14.16	3.395E-07	1.3E-05	7.5	Control			14.97	PC 32:2	PC 14:0/18:2	M+H	C40H76NO8P	-1.807
832.5847	14.21	0.0055385	0.010534	1.6	Control			21.92	PC 40:7	PC 18:2/22:5	M+H, M+K	C48H82NO8P	-0.511
854.5657	14.21	0.0062976	0.011508	1.5	Control			18.62	PC 42:10		M+H	C50H80NO8P	-4.345
806.5701	14.27	0.0285983	0.033704	1.3	Control			11.65	PC 38:6	PC 18:2/20:4	M+H, M+Na	C46H80NO8P	0.821
794.5679	14.35	3.64E-06	6.34E-05	2.6	Control			11.25	PC 37:5	PC 17:0/20:5	M+H	C45H80NO8P	-1.923
782.5690	14.36	0.0061964	0.0114	1.5	GM			11.42	PC 36:4	PC 18:2/18:2	M+H	C44H80NO8P	-0.539
858.5983	14.36	0.0001073	0.000623	1.8	GM			16.36	PC 42:8	PC 20:2/22:6	M+H	C50H84NO8P	-2.811
874.5914	14.37	7.976E-07	2.21E-05	5.5	Control			22.45	PC 42:8;O		M+H, M+Na		
900.6089	14.51	1.285E-06	2.99E-05	9.9	Control			23.03	PC 44:9;O		M+H, M+Na		
522.3552	14.52	0.0002864	0.00125	1.9	Control			17.24	LPC 18:1		M+H, M+Na	C26H52NO7P	-0.402
820.5784	14.52	0.0001525	0.000795	1.7	Control			12.75	PC 39:6	PC 17:0/22:6	M+H	C47H82NO8P	-8.202
732.5533	14.53	1.85E-08	1.87E-06	6.8	Control			10.9	PC 32:1	PC 14:0/18:1 | 16:0/16:1	M+H, M+Na	C40H78NO8P	-0.678
784.5839	14.56	0.0025862	0.006226	1.6	Control			16.26	PC 36:3	PC 18:1/18:2	M+H	C44H82NO8P	-1.490
706.5372	14.64	1.366E-07	6.63E-06	6.0	Control			8.82	PC 30:0	PC 14:0/16:0	M+H, M+Na	C38H76NO8P	-1.355
758.5698	14.64	0.006837	0.012221	1.5	Control			9.81	PC 34:2	PC 16:1/18:1	M+H	C42H80NO8P	0.501
790.5396	14.68	0.0033403	0.002686	3.2	Control			23.73			Neg		
860.6147	14.71	0.0006978	0.002454	1.7	GM			17.9	PC 42:7	PC 20:1/22:6	M+H, M+Na	C50H86NO8P	-2.001
824.5454	14.74	0.0175286	0.007965	1.5	Control			10.11	PC 36:5	PC 18:2/18:3	M+FA-H	C44H78NO8P	0.891
746.5679	14.76	4.296E-08	2.87E-06	3.4	Control			6.42	PC 33:1		M+H	C41H80NO8P	-2.106
792.5890	14.77	1.755E-05	0.000167	2.5	Control			8.08	PC O-38:6	PC P-18:0/20:5	M+H	C46H82NO7P	-1.463
822.5993	14.77	1.313E-05	0.000142	2.1	Control			16.22	PC 39:5		M+H	C47H84NO8P	-1.778
766.5723	14.85	0.0001198	0.000668	2.1	Control			10.91	PC O-36:5		M+H	C44H80NO7P	-2.938
810.5972	14.85	0.0245286	0.030172	1.3	GM			14.06	PC 38:4	PC 18:1/20:3	M+H	C46H84NO8P	-4.339
818.6047	14.90	3.77E-06	6.4E-05	3.1	Control			4.51	PC O-40:7	PC P-18:0/22:6	M+H, M+Na	C48H84NO7P	-1.308
786.6003	14.93	1.49E-07	6.63E-06	3.6	Control			15.57	PC 36:2	PC 18:1/18:1	M+H	C44H84NO8P	-0.545
862.6301	14.95	0.0001186	0.000668	1.9	GM			19.17	PC 42:6	PC 20:2/22:4	M+H	C50H88NO8P	-2.201
836.6134	15.01	0.0011413	0.003558	1.6	GM			16.26	PC 40:5	PC 18:0/22:5	M+H	C48H86NO8P	-3.527
760.5850	15.03	6.189E-06	8.7E-05	2.0	Control			7.87	PC 34:1	PC 16:0/18:1	M+H, M+K	C42H82NO8P	-0.076
838.6184	15.10	0.0012465	0.003775	1.6	GM			14.07	PC 40:4	PC 18:0/22:4	M+H	C48H88NO8P	-16.270
878.5921	15.19	0.0042171	0.003136	1.9	Control			22.39	PC 40:6	PC 18:1/22:5	M+FA-H	C48H84NO8P	0.553
774.5988	15.31	3.745E-08	2.67E-06	3.3	Control			8.16	PC 35:1	PC 17:0/18:1	M+H	C43H84NO8P	-2.448
718.5727	15.37	6.476E-08	3.85E-06	11.4	Control			9.68	PC O-32:1		M+H	C40H80NO7P	-2.534
788.6162	15.63	0.0031249	0.00723	1.3	Control			9.27	PC 36:1	PC 18:0/18:1	M+H	C44H86NO8P	-0.193
675.5431	15.87	5.163E-05	0.000366	1.9	Control			12.59	SM 32:1;O2		M+H, M+Na	C37H75N2O6P	-0.610
806.4964	17.95	0.0011894	0.001458	2.2	Control			6.54	PS 38:6	PS 16:0/22:6	M-H		

Supplementary Table S8. SFC-QTOF analysis of intestine tissue at the intermediate time point. Simple ID was designated by SFC-QTOF, triple quad ID was determined by LC-MS/MS. Green and red represent high and low peak areas, respectively. Red triple quad IDs mean multiple acyl isomers were detected.

m/z	RT	Anova (p)	q Value	Fold	Highest	Co.	GM	CV%	Simple ID	Triple Quad ID	Adducts	Formula	M.E. (ppm)
554.4788	5.21	0.006341	0.006756	1.5	Control			9.63	Cer 32:1;O2		M-H, M+Cl, M+FA-H	C32H63NO3	-0.388
690.6034	5.67	0.000358	0.000583	1.9	GM			7	Cer 42:3;O2		M-H, M+Cl, M+FA-H		
784.4920	9.18	8.98E-05	0.000192	1.8	GM			12.31	PE 40:9	PE 18:3/22:6	M-H	C45H72NO8P	-0.300
810.5063	9.22	1.36E-07	9.49E-07	2.4	GM			8.68	PE 42:10	PE 20:4/22:6	M-H	C47H74NO8P	-2.022
786.5075	9.28	0.000808	0.001158	1.3	GM			2.5	PE 40:8	PE 18:2/22:6	M-H	C45H74NO8P	-0.515
836.5224	9.28	9.14E-08	6.72E-07	1.9	GM			6.86	PE 44:11	PE 22:5/22:6	M-H	C49H76NO8P	-1.412
774.5070	9.32	2.83E-09	4.51E-08	3.4	Control			8.76	PE 39:7	PE 17:1/22:6	M-H	C44H74NO8P	-1.186
736.4921	9.34	0.000127	0.00025	1.5	Control			11.2	PE 36:5	PE 16:0/20:5	M-H	C41H72NO8P	-0.282
764.5219	9.35	0.00063	0.000609	1.3	Control			5.58	PE 38:6	PE 18:2/20:4	M+H, M+Na, M+K	C43H74NO8P	-0.814
812.5225	9.42	5.33E-06	1.7E-05	2.5	GM			13.64	PE 42:9	PE 20:3/22:6	M-H	C47H76NO8P	-1.328
856.5108	9.42	2.16E-06	8.25E-06	2.0	Control			10.74			Neg		
788.5232	9.42	3.64E-07	1.84E-06	1.9	Control			8.78	PE 40:7	PE 18:1/22:6	M-H2O-H, M-H	C45H76NO8P	-0.524
738.5071	9.43	1.36E-09	2.78E-08	3.0	GM			5.98	PE 36:4	PE 16:0/20:4	M-H	C41H74NO8P	-1.095
740.5225	9.55	7.25E-07	3.25E-06	1.9	Control			7.16	PE 36:3	PE 18:1/18:2	M-H	C41H76NO8P	-1.488
772.5284	9.57	0.000214	0.000401	1.7	Control			13.27	PE O-40:8	PE P-18:1/22:6	M-H	C45H76NO7P	-0.405
748.5261	9.59	0.000258	0.000466	2.0	GM			12.91	PE O-38:6	PE P-16:0/22:5|18:1/20:4	M-H	C43H76NO7P	-3.470
792.5529	9.61	7.18E-05	9.06E-05	1.5	GM			8.06	PE 40:6	PE 18:0/22:6	M+H, M+Na	C45H78NO8P	-1.098
816.5543	9.67	0.002688	0.003131	1.4	Control			8.77	PE 42:7	PE 42:7	M-H	C47H80NO8P	-0.701
766.5386	9.70	4.43E-11	6.34E-09	3.3	GM			6.48	PE 38:4	PE 18:1/20:3	M-H	C43H78NO8P	-0.766
768.5446	9.70	7.78E-10	2.16E-08	2.8	GM			7.35	PE 38:3	PE 18:2/20:1	M-H	C43H80NO8P	-13.369
742.5372	9.72	4.45E-08	3.99E-07	2.8	Control			12.15	PE 36:2	PE 18:1/18:1	M-H	C41H78NO8P	-2.708
792.5539	9.82	2.39E-08	2.63E-07	2.1	GM			3.4	PE 40:5	PE 18:0/22:5	M-H	C45H80NO8P	-1.243
774.5433	9.87	7.37E-05	0.00017	3.0	GM			16.77	PE O-40:7		M-H	C45H78NO7P	-1.304
794.5690	9.94	1.77E-14	5.06E-12	6.4	GM			3.4	PE 40:4	PE 18:0/22:4	M-H	C45H82NO8P	-1.943
810.5619	9.95	8.3E-10	2.16E-08	6.6	Control			13.25	PE 40:4;O		M-H		
818.5700	9.95	2.27E-08	2.6E-07	3.3	GM			7.89	PE 42:6	PE 20:0/22:6	M-H	C47H82NO8P	-0.614
784.5492	9.99	9.21E-06	2.64E-05	3.4	Control			24.22	PE 38:3;O		M-H		
770.5674	10.00	5.04E-06	1.62E-05	3.7	Control			20.35	PE 38:2	PE 18:1/20:1	M-H	C43H82NO8P	-4.036
820.5844	10.08	3.63E-08	3.72E-07	3.7	GM			13.28	PE 42:5	PE 20:1/22:4	M-H	C47H84NO8P	-2.111
798.5972	10.27	3.68E-06	1.24E-05	8.2	Control			43.24	PE 40:2		M-H	C45H86NO8P	-5.820
812.5803	10.28	0.000393	0.000633	2.5	Control			26.46	PE 40:3;O		M-H		
881.5178	10.33	0.001781	0.002279	2.4	Control			35.4	PI 38:6		M-H	C47H79O13P	-0.868
883.5322	10.44	1.51E-05	4.2E-05	3.2	Control			22.71	PI 38:5		M-H	C47H81O13P	-2.232
887.5619	10.66	0.000324	0.00055	2.6	Control			16.97	PI 38:3		M-H	C47H85O13P	-4.097
1543.9591	11.92	0.002422	0.002928	1.5	Control			9.28	CL 80:16		M-2H, M-H	C89H142O17P2	-3.775
898.5913	14.13	0.000316	0.000332	3.9	Control			22.53	PC 44:10;O		M-H		
826.5365	14.52	0.001156	0.001009	1.8	GM			16.97	PC 40:10	PC 20:5/20:5|18:4/22:6	M+H	C48H76NO8P	-1.979
832.5121	14.56	3.79E-09	5.43E-08	3.1	Control			7.6	PC 37:8		M+FA-H		
806.4986	14.59	4.99E-07	2.34E-06	1.7	Control			7.74	PC 35:7		M+FA-H		
542.3241	14.62	5.52E-05	7.23E-05	3.2	GM			11.43	LPC 20:5		M+H	C28H48NO7P	-0.115
852.5530	14.63	8.94E-05	0.000109	1.7	GM			12.85	PC 42:11	PC 20:5/22:6	M+H, M+Na	C50H78NO8P	-0.875
802.5372	14.75	0.000487	0.000478	2.8	GM			22.57	PC 38:8	PC 18:3/20:5	M+H	C46H76NO8P	-1.162
828.5526	14.88	0.000362	0.000365	2.3	GM			15.38	PC 40:9	PC 20:4/20:5	M+H, M+Na	C48H78NO8P	-1.472
854.5680	14.89	1.56E-07	4.95E-07	3.2	GM			10.2	PC 42:10	PC 20:5/22:5	M+H	C50H80NO8P	-1.681
752.5231	14.99	5.73E-06	1.07E-05	2.3	Control			8.41	PC 34:5	PC 14:0/20:5	M+H	C42H74NO8P	0.788
880.5832	15.00	0.000294	0.000315	1.8	GM			11.58	PC 44:11	PC 22:5/22:6	M+H, M+Na		
860.5456	15.04	0.000249	0.000451	1.4	Control			9.72	PC 39:8		M+FA-H		
778.5376	15.11	0.000985	0.000889	1.6	Control			9.4	PC 36:6	PC 16:1/20:5	M+H, M+Na	C44H76NO8P	-0.686

<table>
<tr><td>830.5677</td><td>15.16</td><td>0.007428</td><td>0.00496</td><td>1.5</td><td>GM</td><td></td><td></td><td>6.99</td><td>PC 40:8</td><td>PC 18:3/22:5</td><td>M+H</td><td>C48H80NO8P</td><td>-2.115</td></tr>
<tr><td>856.5830</td><td>15.18</td><td>6.73E-06</td><td>1.24E-05</td><td>2.7</td><td>GM</td><td></td><td></td><td>8.46</td><td>PC 42:9</td><td>PC 20:4/22:5</td><td>M+H</td><td>C50H82NO8P</td><td>-2.442</td></tr>
<tr><td>894.5965</td><td>15.23</td><td>4.86E-07</td><td>1.28E-06</td><td>2.6</td><td>Control</td><td></td><td></td><td>7.45</td><td>PC 45:11</td><td></td><td>M+H</td><td></td><td></td></tr>
<tr><td>882.5962</td><td>15.25</td><td>1.55E-07</td><td>4.95E-07</td><td>3.8</td><td>GM</td><td></td><td></td><td>9.82</td><td>PC 44:10</td><td></td><td>M+H</td><td>C52H84NO8P</td><td>-5.139</td></tr>
<tr><td>818.5649</td><td>15.30</td><td>6.7E-07</td><td>1.55E-06</td><td>2.0</td><td>Control</td><td></td><td></td><td>6.23</td><td>PC 39:7</td><td></td><td>M+H</td><td>C47H80NO8P</td><td>-5.557</td></tr>
<tr><td>792.5527</td><td>15.37</td><td>3.03E-05</td><td>4.45E-05</td><td>1.7</td><td>Control</td><td></td><td></td><td>6.65</td><td>PC 37:6</td><td>PC 15:0/22:6</td><td>M+H</td><td>C45H78NO8P</td><td>-1.408</td></tr>
<tr><td>780.5545</td><td>15.48</td><td>0.017328</td><td>0.010398</td><td>1.2</td><td>GM</td><td></td><td></td><td>8.1</td><td>PC 36:5</td><td>PC 18:2/18:3</td><td>M+H</td><td>C44H78NO8P</td><td>0.956</td></tr>
<tr><td>832.5846</td><td>15.52</td><td>0.000335</td><td>0.000343</td><td>1.8</td><td>Control</td><td></td><td></td><td>12.67</td><td>PC 40:7</td><td>PC 18:2/22:5</td><td>M+H, M+Na</td><td>C48H82NO8P</td><td>-1.909</td></tr>
<tr><td>730.5381</td><td>15.56</td><td>7.38E-09</td><td>5.88E-08</td><td>2.9</td><td>Control</td><td></td><td></td><td>8.82</td><td>PC 32:2</td><td>PC 14:0/18:2</td><td>M+H</td><td>C40H76NO8P</td><td>-0.055</td></tr>
<tr><td>874.5922</td><td>15.62</td><td>9.52E-08</td><td>3.32E-07</td><td>5.3</td><td>Control</td><td></td><td></td><td>9.65</td><td>PC 42:8;O</td><td></td><td>M+H</td><td></td><td></td></tr>
<tr><td>850.5596</td><td>15.64</td><td>0.000481</td><td>0.000742</td><td>1.3</td><td>Control</td><td></td><td></td><td>7.53</td><td>PC 38:6</td><td>PC 18:2/20:4</td><td>M+FA-H</td><td>C46H80NO8P</td><td>-0.960</td></tr>
<tr><td>918.5468</td><td>15.64</td><td>8.75E-05</td><td>0.000188</td><td>1.4</td><td>Control</td><td></td><td></td><td>8.28</td><td></td><td></td><td>Neg</td><td></td><td></td></tr>
<tr><td>766.5374</td><td>15.80</td><td>2.03E-09</td><td>3.64E-08</td><td>2.8</td><td>GM</td><td></td><td></td><td>5.68</td><td></td><td></td><td>Neg</td><td></td><td></td></tr>
<tr><td>782.5684</td><td>15.80</td><td>4.74E-09</td><td>5.31E-08</td><td>2.7</td><td>GM</td><td></td><td></td><td>5.38</td><td>PC 36:4</td><td>PC 18:2/18:2</td><td>M+H</td><td>C44H80NO8P</td><td>-1.305</td></tr>
<tr><td>900.6089</td><td>15.81</td><td>7.82E-08</td><td>2.88E-07</td><td>9.9</td><td>Control</td><td></td><td></td><td>11.82</td><td>PC 44:9;O</td><td></td><td>M+H</td><td></td><td></td></tr>
<tr><td>820.5826</td><td>15.99</td><td>3.09E-05</td><td>4.47E-05</td><td>1.6</td><td>Control</td><td></td><td></td><td>7.86</td><td>PC 39:6</td><td></td><td>M+H</td><td>C47H82NO8P</td><td>-3.005</td></tr>
<tr><td>896.5704</td><td>16.05</td><td>2.33E-10</td><td>9.54E-09</td><td>2.3</td><td>Control</td><td></td><td></td><td>4.29</td><td></td><td></td><td>Neg</td><td></td><td></td></tr>
<tr><td>848.6074</td><td>16.06</td><td>5.54E-07</td><td>1.35E-06</td><td>2.1</td><td>Control</td><td></td><td></td><td>7.38</td><td>PC 41:6</td><td></td><td>M+H</td><td>C49H86NO8P</td><td>-10.627</td></tr>
<tr><td>784.5830</td><td>16.10</td><td>1.85E-05</td><td>2.94E-05</td><td>1.6</td><td>Control</td><td></td><td></td><td>7.44</td><td>PC 36:3</td><td>PC 16:0/20:3</td><td>M+H</td><td>C44H82NO8P</td><td>-2.695</td></tr>
<tr><td>808.5834</td><td>16.10</td><td>0.000331</td><td>0.000342</td><td>1.4</td><td>GM</td><td></td><td></td><td>6.51</td><td>PC 38:5</td><td>PC 16:0/22:5</td><td>M+H</td><td>C46H82NO8P</td><td>-2.034</td></tr>
<tr><td>732.5527</td><td>16.14</td><td>7E-08</td><td>2.67E-07</td><td>2.2</td><td>Control</td><td></td><td></td><td>9.57</td><td>PC 32:1</td><td>PC 16:0/16:1|14:0/18:1</td><td>M+H, M+Na</td><td>C40H78NO8P</td><td>-1.487</td></tr>
<tr><td>758.5686</td><td>16.22</td><td>1.59E-05</td><td>2.61E-05</td><td>1.5</td><td>Control</td><td></td><td></td><td>8.38</td><td>PC 34:2</td><td>PC 16:0/18:2</td><td>M+H, M+Na</td><td>C42H80NO8P</td><td>-0.380</td></tr>
<tr><td>524.3711</td><td>16.27</td><td>0.000135</td><td>0.000153</td><td>2.0</td><td>GM</td><td></td><td></td><td>14.32</td><td>LPC 18:0</td><td></td><td>M+H, M+Na</td><td>C26H54NO7P</td><td>-0.018</td></tr>
<tr><td>706.5365</td><td>16.30</td><td>9.37E-09</td><td>6.71E-08</td><td>2.9</td><td>Control</td><td></td><td></td><td>7.91</td><td>PC 30:0</td><td></td><td>M+H</td><td>C38H76NO8P</td><td>-2.248</td></tr>
<tr><td>772.5826</td><td>16.30</td><td>8.92E-10</td><td>1.53E-08</td><td>2.9</td><td>Control</td><td></td><td></td><td>1.57</td><td>PC 35:2</td><td></td><td>M+H</td><td>C43H82NO8P</td><td>-3.183</td></tr>
<tr><td>836.6093</td><td>16.32</td><td>9.68E-06</td><td>1.69E-05</td><td>1.9</td><td>GM</td><td></td><td></td><td>10.35</td><td>PC 40:5</td><td>PC 18:0/22:5</td><td>M+H</td><td>C48H86NO8P</td><td>-8.425</td></tr>
<tr><td>852.6084</td><td>16.34</td><td>1.67E-08</td><td>1.03E-07</td><td>9.2</td><td>Control</td><td></td><td></td><td>21.97</td><td>PC 40:5;O</td><td></td><td>M+H</td><td></td><td></td></tr>
<tr><td>862.5807</td><td>16.39</td><td>0.006446</td><td>0.006817</td><td>1.3</td><td>Control</td><td></td><td></td><td>5.12</td><td></td><td>PS 20:0/22:6</td><td>Neg</td><td></td><td></td></tr>
<tr><td>818.6043</td><td>16.40</td><td>4.73E-06</td><td>9.2E-06</td><td>2.4</td><td>Control</td><td></td><td></td><td>8.84</td><td>PC O-40:7</td><td>PC P-18:0/22:6</td><td>M+H, M+Na</td><td>C48H84NO7P</td><td>-1.907</td></tr>
<tr><td>746.5673</td><td>16.46</td><td>3.35E-07</td><td>9.92E-07</td><td>1.9</td><td>Control</td><td></td><td></td><td>6.85</td><td>PC 33:1</td><td></td><td>M+H</td><td>C41H80NO8P</td><td>-2.877</td></tr>
<tr><td>810.5995</td><td>16.50</td><td>1.32E-10</td><td>4.5E-09</td><td>3.3</td><td>GM</td><td></td><td></td><td>6.28</td><td>PC 38:4</td><td>PC 18:2/20:2</td><td>M+H</td><td>C46H84NO8P</td><td>-1.549</td></tr>
<tr><td>898.5853</td><td>16.58</td><td>1.19E-10</td><td>6.95E-09</td><td>3.2</td><td>Control</td><td></td><td></td><td>5.98</td><td></td><td></td><td>Neg</td><td></td><td></td></tr>
<tr><td>786.5995</td><td>16.62</td><td>2.66E-09</td><td>3.29E-08</td><td>2.5</td><td>Control</td><td></td><td></td><td>7.08</td><td>PC 36:2</td><td>PC 18:1/18:1</td><td>M+H</td><td>C44H84NO8P</td><td>-1.532</td></tr>
<tr><td>788.6046</td><td>16.62</td><td>5.06E-09</td><td>5.31E-08</td><td>2.6</td><td>Control</td><td></td><td></td><td>7.27</td><td>PC 36:1</td><td>PC 18:0/18:1</td><td>M+H</td><td>C44H86NO8P</td><td>-14.943</td></tr>
<tr><td>872.5624</td><td>16.76</td><td>0.000694</td><td>0.001015</td><td>1.3</td><td>Control</td><td></td><td></td><td>8.19</td><td></td><td></td><td>Neg</td><td></td><td></td></tr>
<tr><td>854.6236</td><td>16.90</td><td>1.01E-09</td><td>1.53E-08</td><td>10.3</td><td>Control</td><td></td><td></td><td>14.2</td><td>PC 40:4;O</td><td></td><td>M+H</td><td></td><td></td></tr>
<tr><td>862.6289</td><td>17.07</td><td>4.9E-06</td><td>9.4E-06</td><td>2.6</td><td>GM</td><td></td><td></td><td>13.2</td><td>PC 42:6</td><td>PC 20:0/22:6</td><td>M+H</td><td>C50H88NO8P</td><td>-3.606</td></tr>
<tr><td>838.6243</td><td>17.09</td><td>1.39E-07</td><td>4.74E-07</td><td>2.4</td><td>GM</td><td></td><td></td><td>8.55</td><td>PC 40:4</td><td>PC 20:0/20:4</td><td>M+H</td><td>C48H88NO8P</td><td>-9.256</td></tr>
<tr><td>734.5683</td><td>17.11</td><td>0.001749</td><td>0.001428</td><td>1.3</td><td>GM</td><td></td><td></td><td>8.87</td><td>PC 32:0</td><td>PC 16:0/16:0</td><td>M+H</td><td>C40H80NO8P</td><td>-1.577</td></tr>
<tr><td>675.5429</td><td>17.45</td><td>0.00028</td><td>0.000305</td><td>1.6</td><td>Control</td><td></td><td></td><td>12.14</td><td>SM 32:1;O2</td><td></td><td>M+H</td><td>C37H75N2O6P</td><td>-0.997</td></tr>
<tr><td>814.6302</td><td>17.48</td><td>1.43E-05</td><td>2.4E-05</td><td>2.0</td><td>Control</td><td></td><td></td><td>14.53</td><td>PC 38:2</td><td>PC 18:1/20:1</td><td>M+H</td><td>C46H88NO8P</td><td>-2.248</td></tr>
<tr><td>842.6616</td><td>18.43</td><td>4.27E-07</td><td>1.21E-06</td><td>3.2</td><td>Control</td><td></td><td></td><td>14.51</td><td>PC 40:2</td><td>PC 18:1/22:1</td><td>M+H</td><td>C48H92NO8P</td><td>-2.045</td></tr>
<tr><td>811.6665</td><td>20.22</td><td>8.76E-05</td><td>0.000108</td><td>1.9</td><td>GM</td><td></td><td></td><td>13.98</td><td>SM 42:3;O2</td><td></td><td>M+H</td><td>C47H91N2O6P</td><td>-2.752</td></tr>
</table>

Supplementary Table S9. SFC-QTOF analysis of liver tissue at the final time point. Simple ID was designated by SFC-QTOF, triple quad ID was determined by LC-MS/MS. Green and red represent high and low peak areas, respectively. Red triple quad IDs mean multiple acyl isomers were detected.

m/z	RT	Anova (p)	q Value	Fold	Highest	Co.	GM	CV%	Simple ID	Triple Quad ID	Adducts	Formula	M.E. (ppm)
554.4792	4.97	0.002949	0.002091	1.9	Control			14.63	Cer 32:1;O2		M+Cl, M+FA-H	C32H63NO3	0.326
604.4947	5.02	0.003721	0.002526	3.3	GM			46.46	Cer 36:4;O2		M-H, M+Cl, M+FA-H		
608.5254	5.10	0.007658	0.004575	3.0	Control			34.27	Cer 36:2:O2		M-H, M+FA-H	C36H69NO3	-0.953
688.5889	5.48	0.000359	0.000412	1.5	GM			10.85	Cer 42:4;O2		M-H, M+FA-H		
694.6351	5.66	0.000217	0.000291	2.1	Control			19.93	Cer 42:1;O2		M+FA-H	C42H83NO3	-0.639
708.6157	5.73	0.002651	0.00192	2.2	GM			27.59	Cer 42:2;O3		M+FA-H	C42H81NO4	1.451
869.5302	7.36	0.000134	0.000226	2.3	GM			21.77	PG 44:10	PG 22:5/22:5	M-H	C50H79O10P	-4.195
854.6731	7.64	0.001298	0.001077	1.4	Control			8.57	HexCer 42:2;O2		M-H, M+Cl, M+FA-H	C48H91NO8	0.534
765.4718	7.87	1.24E-06	8.04E-06	14.0	Control			22.77	PG 36:6	PG 14:0/22:6	M-H	C42H71O10P	0.790
791.4876	7.90	9.32E-05	0.000175	3.2	Control			22.69	PG 38:7	PG 18:2/20:5	M-H	C44H73O10P	0.884
767.4875	7.98	0.000125	0.000221	3.8	Control			32.6	PG 36:5	PG 16:0/20:5	M-H	C42H73O10P	0.887
817.5029	7.98	0.011522	0.006293	1.8	Control			24.61	PG 40:8	PG 18:2/22:6	M-H	C46H75O10P	0.522
793.5032	8.06	0.00041	0.000452	3.2	Control			32.45	PG 38:6	PG 18:1/20:5	M-H	C44H75O10P	0.885
819.5185	8.08	3.96E-05	8.89E-05	5.2	Control			33.5	PG 40:7	PG 18:1/22:6	M-H	C46H77O10P	0.457
745.5028	8.10	0.000158	0.000243	5.2	Control			36.42	PG 34:2		M-H	C40H75O10P	0.413
771.5181	8.11	0.000112	0.000199	5.3	Control			41.83	PG 36:3	PG 16:0/20:3	M-H	C42H77O10P	-0.064
795.5186	8.15	0.002111	0.00161	2.5	Control			32.71	PG 38:5	PG 16:0/22:5	M-H	C44H77O10P	0.523
821.5338	8.16	0.000418	0.000458	4.0	Control			27.52	PG 40:6	PG 18:0/22:6	M-H	C46H79O10P	-0.053
870.6682	8.17	0.000962	0.000854	1.9	GM			11.83	HexCer 42:2;O3		M-H, M+FA-H	C48H91NO9	0.697
747.5188	8.23	6.6E-06	2.57E-05	3.8	Control			19.19	PG 34:1	PG 16:0/18:1	M-H	C40H77O10P	0.915
771.5188	8.23	3.99E-07	3.56E-06	4.3	GM			20.85	PG 36:3		M-H	C42H77O10P	0.841
821.5344	8.25	0.01234	0.00657	2.6	Control			43.86	PG 40:6		M-H	C46H79O10P	0.666
773.5337	8.31	0.031962	0.014652	1.4	Control			20.9	PG 36:2	PG 16:0/20:2	M-H	C42H79O10P	-0.159
555.2733	8.41	0.011571	0.006304	2.9	Control			41.32	LPG 22:6		M-H	C28H45O9P	0.799
509.2881	8.54	0.001329	0.001099	9.7	Control			58.87	LPG 18:1		M-H	C24H47O9P	-0.783
591.3907	8.93	0.000267	0.000333	3.4	GM			18.35			Neg		
758.4770	8.99	0.000334	0.000394	3.6	GM			39.41	PE 38:8	PE 18:3/20:5	M-H	C43H70NO8P	0.485
784.4926	9.07	2.63E-05	6.7E-05	4.1	GM			23.06	PE 40:9	PE 18:3/22:6	M-H	C45H72NO8P	0.469
810.5080	9.11	0.000236	0.000308	3.1	GM			25.36	PE 42:10	PE 20:4/22:6	M-H	C47H74NO8P	0.032
786.5083	9.16	0.002166	0.001627	1.7	GM			11.67	PE 40:8	PE 18:3/22:5	M-H	C45H74NO8P	0.531
774.5081	9.18	1.31E-07	1.73E-06	5.6	Control			18.37	PE 39:7		M-H	C44H74NO8P	0.250
736.4930	9.19	0.002466	0.001817	1.4	Control			14.02	PE 36:5	PE 16:0/20:5	M-H	C41H72NO8P	1.003
762.5083	9.19	0.000947	0.000848	1.6	Control			14.4	PE 38:6	PE 18:2/20:4	M-H	C43H74NO8P	0.501
738.5082	9.27	2.4E-07	2.52E-06	3.9	GM			17.03	PE 36:4	PE 16:0/20:4	M-H	C41H74NO8P	0.379
764.5209	9.27	0.010237	0.005711	1.4	GM			11.97	PE 38:5	PE 18:1/20:4	M-H	C43H76NO8P	-3.561
788.5238	9.28	0.001059	0.000924	1.6	Control			12.09	PE 40:7	PE 18:2/22:5	M-H	C45H76NO8P	0.333
856.5110	9.28	0.000266	0.000333	1.9	Control			12.9			Neg		
812.5238	9.29	5.78E-07	4.61E-06	6.2	GM			15.22	PE 42:9	PE 20:3/22:6	M-H	C47H76NO8P	0.237
740.5232	9.38	5.46E-06	2.24E-05	3.2	Control			15.44	PE 36:3	PE 18:1/18:2	M-H	C41H76NO8P	-0.535
814.5394	9.39	0.000594	0.000588	1.9	GM			10.64	PE 42:8	PE 20:2/22:6	M-H	C47H78NO8P	0.185
764.5240	9.42	0.058626	0.024405	1.2	Control			11.92	PE 38:5		M-H	C43H76NO8P	0.601
772.5294	9.42	0.004236	0.002832	1.6	Control			15.08	PE O-40:8		M-H	C45H76NO7P	0.966
498.2627	9.49	0.008599	0.004989	4.0	Control			40.6	LPE 20:5		M-H	C25H42NO7P	0.199
792.5552	9.51	0.028064	0.085195	1.3	GM			14.34	PE 40:6	PE 18:1/22:5	M+H, M+Na	C45H78NO8P	1.845
766.5390	9.52	3.08E-06	1.46E-05	2.5	GM			15.79	PE 38:4	PE 18:1/20:3	M-H	C43H78NO8P	-0.350
792.5509	9.52	1.81E-05	5.21E-05	2.1	GM			7.34	PE 40:5	PE 18:0/22:5	M-H	C45H80NO8P	-5.035
816.5553	9.52	0.010966	0.00605	1.4	GM			12.36	PE 42:7	PE 20:1/22:6	M-H	C47H80NO8P	0.494
742.5386	9.53	7.99E-08	1.21E-06	5.3	Control			20.83	PE 36:2	PE 18:1/18:1	M-H	C41H78NO8P	-0.909
818.5704	9.63	0.008096	0.004785	1.5	GM			14.85	PE 42:6	PE 20:0/22:6	M-H	C47H82NO8P	-0.185
792.5551	9.64	0.0005	0.000525	1.4	GM			11.68	PE 40:5		M-H	C45H80NO8P	0.317
524.2788	9.75	0.025177	0.011827	1.8	Control			26.93	LPE 22:6		M-H	C27H44NO7P	1.040
794.5702	9.76	7.29E-09	2.76E-07	7.4	GM			17.62	PE 40:4	PE 20:1/20:3	M-H	C45H82NO8P	-0.410
818.5711	9.78	4.53E-08	8.59E-07	5.6	GM			14.07	PE 42:6		M-H	C47H82NO8P	0.739
820.5857	9.90	4.82E-08	8.6E-07	8.4	GM			20.11	PE 42:5	PE 20:0/20:5	M-H	C47H84NO8P	-0.559
478.2942	9.91	0.000184	0.000263	4.0	Control			22.36	LPE 18:1		M-H	C23H46NO7P	0.564
881.5192	10.27	0.000637	0.00062	2.6	Control			31.27	PI 38:6	PI 18:1/20:5	M-H	C47H79O13P	0.766
883.5344	10.38	8.07E-05	0.000157	2.8	Control			21.9	PI 38:5	PI 18:1/20:4	M-H	C47H81O13P	0.257
909.5473	10.38	0.000456	0.004501	1.6	GM			11.39	PI 40:7		M+H, M+Na	C49H81O13P	-1.558

949.5287	10.39	0.000177	0.002271	1.8	GM			15.93	PI O-43:12		M+NH4, M+Na		
885.5505	10.47	0.012991	0.00678	1.3	GM			9.63	PI 38:4	PI 18:0/20:4	M-H	C47H83O13P	0.695
911.5644	10.55	0.003002	0.002118	1.6	GM			22.16	PI 40:5	PI 18:0/22:5	M-H	C49H85O13P	-1.209
913.5772	10.65	5.21E-05	0.000113	2.8	GM			20.91	PI 40:4	PI 20:0/20:4|18:0/22:4	M-H	C49H87O13P	-4.311
852.5549	14.36	0.00075	0.006436	1.9	GM			19.95	PC 42:11	PC 20:5/22:6	M+H	C50H78NO8P	1.327
802.5392	14.45	5.8E-05	0.001071	5.0	GM			35.17	PC 38:8	PC 18:3/20:5	M+H-H2O, M+H, M+Na	C46H76NO8P	1.314
878.5706	14.49	0.000741	0.00639	1.8	GM			21.44	PC 44:12	PC 22:6/22:6	M+H, M+Na	C52H80NO8P	1.319
788.5231	14.56	0.006133	0.003837	2.3	GM			32.39	PC 37:7		M-H	C45H76NO8P	-0.607
828.5550	14.58	6E-06	0.000219	5.2	GM			27.77	PC 40:9	PC 20:4/20:5	M+H, M+Na	C48H78NO8P	1.418
854.5696	14.61	1.71E-06	9.76E-05	4.4	GM			23.91	PC 42:10	PC 20:5/22:5	M+H, M+Na	C50H80NO8P	0.212
752.5224	14.62	0.00011	0.001598	3.0	Control			19.11	PC 34:5	PC 14:0/20:5	M+H, M+Na	C42H74NO8P	-0.075
804.5556	14.67	0.001057	0.007988	2.1	GM			23.84	PC 38:7	PC 18:3/20:4	M+H, M+Na	C46H78NO8P	2.296
880.5853	14.68	4.17E-06	0.000175	3.2	GM			17.27	PC 44:11	PC 22:5/22:6	M+H, M+Na	C52H82NO8P	0.253
778.5395	14.71	0.015843	0.05645	1.5	Control			16.36	PC 36:6	PC 18:3/18:3	M+H, M+Na	C44H76NO8P	1.740
830.5714	14.79	1.97E-05	0.000497	2.9	GM			21.38	PC 40:8	PC 18:3/22:5	M+H	C48H80NO8P	2.399
852.5535	14.79	2.31E-05	0.00055	2.8	GM			19.61	PC 42:11		M+H		
856.5858	14.81	3.26E-07	3.64E-05	5.4	GM			22.97	PC 42:9	PC 20:4/22:5	M+H, M+Na	C50H82NO8P	0.889
882.6001	14.92	3.93E-06	0.000175	4.5	GM			19.68	PC 44:10	PC 22:5/22:5	M+H, M+Na	C52H84NO8P	-0.669
894.5997	14.93	1.81E-05	0.000485	3.8	Control			29.65	PC 45:11		M+H, M+Na		
792.5548	14.94	0.000409	0.004114	1.6	Control			14.45	PC 37:6	PC 15:0/22:6	M+H	C45H78NO8P	1.311
730.5385	15.09	7.76E-08	2.47E-05	4.4	Control			18.43	PC 32:2	PC 14:0/18:2	M+H	C40H76NO8P	0.479
884.6126	15.15	8.15E-07	6.29E-05	6.1	GM			20.98	PC 44:9		M+H	C52H86NO8P	-4.251
740.5208	15.20	5.97E-05	0.000127	2.4	GM			22.03			Neg		
858.6009	15.23	1.66E-05	0.000459	2.8	GM			22.23	PC 42:8	PC 20:2/22:6	M+H, M+Na	C50H84NO8P	0.195
874.5942	15.24	1.9E-05	0.000497	4.0	Control			32.71	PC 42:8;O		M+H		
794.5690	15.25	2.2E-07	3.21E-05	2.7	Control			12.17	PC 37:5	PC 17:0/20:5	M+H	C45H80NO8P	-0.520
846.6020	15.29	9.53E-05	0.001522	2.2	Control			19.91	PC 41:7		M+H	C49H84NO8P	1.478
782.5708	15.30	2.02E-06	0.000108	2.4	GM			13.94	PC 36:4	PC 18:2/18:2	M+H, M+Na	C44H80NO8P	1.703
718.5357	15.31	1.04E-07	2.47E-05	1.9	Control			6.13	PC 31:1		M+H	C39H76NO8P	-3.386
756.5548	15.33	0.000138	0.001892	1.9	GM			18.96	PC 34:3	PC 16:0/18:3	M+H	C42H78NO8P	1.348
900.6108	15.41	7.63E-05	0.001284	4.2	Control			34.97	PC 44:9;O		M+H, M+Na		
828.5761	15.47	0.038269	0.017129	1.3	Control			14.73	PC 36:3	PC 18:1/18:2	M+FA-H	C44H82NO8P	0.145
820.5810	15.47	0.000211	0.002542	1.7	Control			10.35	PC 39:6		M+H	C47H82NO8P	-4.974
522.3555	15.51	0.003098	0.017989	2.8	Control			39.8	LPC 18:1		M+H	C26H52NO7P	0.160
732.5534	15.53	9.91E-08	2.47E-05	4.0	Control			13.96	PC 32:1	PC 14:0/18:1|16:0/16:1	M+H, M+Na	C40H78NO8P	-0.496
848.6166	15.62	2.11E-05	0.000518	4.5	Control			32.37	PC 41:6		M+H, M+Na	C49H86NO8P	0.219
758.5709	15.63	0.020124	0.067166	1.3	Control			15.76	PC 34:2	PC 16:0/18:2	M+H	C42H80NO8P	1.929
860.6180	15.66	0.000888	0.007258	1.9	GM			20.74	PC 42:7	PC 20:2/22:5	M+H, M+Na	C50H86NO8P	1.849
792.5897	15.72	0.000915	0.007341	1.9	Control			23.51	PC O-38:6	PC O-16:0/22:6	M+H	C46H82NO7P	-0.537
834.6028	15.75	0.014491	0.053084	1.4	GM			16.99	PC 40:6	PC 20:1/20:5	M+H, M+Na	C48H84NO8P	2.483
822.6052	15.76	0.000949	0.007443	1.8	Control			18.63	PC 39:5		M+H	C47H84NO8P	5.394
746.5668	15.79	1.45E-06	8.84E-05	2.2	Control			11.05	PC 33:1		M+H	C41H80NO8P	-3.581
810.5996	15.80	2.39E-05	0.00056	1.9	GM			13.57	PC 38:4	PC 18:2/20:2	M+H	C46H84NO8P	-1.340
716.5550	15.81	2.99E-09	6.31E-06	4.1	Control			12.5	PC O-32:2		M+H	C40H78NO7P	-5.405
818.6054	15.87	0.000241	0.002768	2.1	Control			13.52	PC O-40:7	PC P-18:0/22:6	M+H	C48H84NO7P	-0.570
862.6317	15.93	0.001911	0.012439	1.9	GM			22.9	PC 42:6	PC 20:0/22:6	M+H	C50H88NO8P	-0.353
772.5832	15.95	8.57E-06	0.000292	2.1	Control			12.44	PC 35:2		M+H	C43H82NO8P	-2.433
786.6013	15.98	2.07E-05	0.000518	2.4	Control			15.98	PC 36:2	PC 18:1/18:1	M+H	C44H84NO8P	0.721
836.6172	16.05	0.004491	0.023415	1.7	GM			23.8	PC 40:5	PC 18:0/22:5	M+H	C48H86NO8P	0.934
760.5859	16.10	0.00514	0.025927	1.5	Control			19.4	PC 34:1	PC 16:0/18:1	M+H, M+Na	C42H82NO8P	1.140
800.6189	16.31	6.84E-05	0.001204	2.9	Control			25.77	PC 37:2		M+H	C45H86NO8P	3.196
774.5995	16.37	7.24E-07	6.13E-05	3.0	Control			12.61	PC 35:1		M+H	C43H84NO8P	-1.530
862.6317	16.40	1.34E-05	0.0004	3.4	GM			23.46	PC 42:6		M+H, M+Na	C50H88NO8P	-0.427
882.6177	16.45	1.27E-06	8.04E-06	4.4	GM			18.23	PC 40:4	PC 20:1/20:3	M+FA-H	C48H88NO8P	-6.229
788.6170	16.83	0.024442	0.077332	1.4	Control			19.02	PC 36:1	PC 18:0/18:1	M+H	C44H86NO8P	0.726
802.6315	16.85	8.21E-05	0.001371	4.7	Control			41.07	PC 37:1		M+H	C45H88NO8P	-0.684
772.6194	16.96	1.28E-07	2.55E-05	4.5	Control			10.39	PC O-36:2		M+H	C44H86NO7P	-2.715

Supplementary Table S10. SFC-QTOF analysis of liver tissue at the intermediate time point. Simple ID was designated by SFC-QTOF, triple quad ID was determined by LC-MS/MS. Green and red represent high and low peak areas, respectively. Red triple quad IDs mean multiple acyl isomers were detected.

m/z	RT	Anova (p)	q Value	Fold	Highest	Co.	GM	CV%	Simple ID	Triple Quad ID	Adducts	Formula	M.E. (ppm)
779.6540	5.09	0.000271	0.00019	2.5	Control			23.13					
664.5887	5.61	0.00013	0.000113	2.0	GM			16.61	Cer 40:2;O2		M-H, M+Cl, M+FA-H		
690.6037	5.68	0.006553	0.003449	1.3	GM			10.75	Cer 42:3;O2		M-H, M+Cl, M+FA-H		
867.5160	7.35	3.57E-05	3.42E-05	2.0	GM			16.29	PG 44:11	PG 22:5/22:6	M-H	C50H77O10P	-2.526
869.5303	7.37	3.13E-08	1.02E-07	5.6	GM			18.03	PG 44:10	PG 22:4/22:6	M-H	C50H79O10P	-4.042
791.4849	8.03	0.001329	0.000833	3.9	GM			40.96	PG 38:7	PG 18:2/20:5	M-H	C44H73O10P	-2.424
767.4856	8.06	0.002643	0.001542	4.1	GM			50.73	PG 36:5		M-H	C42H73O10P	-1.583
817.5006	8.06	0.002064	0.001239	3.0	GM			39.34	PG 40:8		M-H	C46H75O10P	-2.296
795.5163	8.27	0.002988	0.001705	4.2	GM			54.08	PG 38:5		M-H	C44H77O10P	-2.379
591.3902	8.97	1.42E-08	6.06E-08	5.5	GM			7.83			Neg		
784.4920	9.21	1.72E-05	1.79E-05	1.9	GM			7.45	PE 40:9	PE 18:3/22:6	M-H	C45H72NO8P	-0.407
810.5063	9.26	1.23E-06	1.87E-06	3.1	GM			7.62	PE 42:10	PE 20:4/22:6	M-H	C47H74NO8P	-2.011
786.5073	9.30	0.000135	0.000115	1.5	GM			4.01	PE 40:8	PE 18:2/22:6	M-H	C45H74NO8P	-0.768
836.5216	9.30	2.99E-08	1.01E-07	3.0	GM			4.99	PE 44:11	PE 22:5/22:6	M-H	C49H76NO8P	-2.312
774.5071	9.34	2.14E-08	7.67E-08	6.0	Control			15.79	PE 39:7	PE 17:1/22:6	M-H	C44H74NO8P	-1.092
788.5228	9.44	3.08E-07	6.09E-07	2.0	Control			5.95	PE 40:7	PE 18:2/22:5	M-H	C45H76NO8P	-0.933
812.5226	9.44	7.31E-08	2.04E-07	3.4	GM			5.08	PE 42:9	PE 20:3/22:6	M-H	C47H76NO8P	-1.233
856.5103	9.44	1.62E-07	3.53E-07	2.0	Control			6.92	PE 42:10		M+FA-H	C47H74NO8P	-3.867
764.5193	9.45	1.46E-06	2.19E-06	1.8	GM			4.06	PE 38:5	PE 16:0/22:5	M-H	C43H76NO8P	-5.564
738.5064	9.46	1.2E-09	8.55E-09	4.3	GM			5.02	PE 36:4	PE 16:0/20:4	M-H	C41H74NO8P	-2.017
764.5221	9.50	0.002151	0.001155	1.5	Control			15.93	PE 38:6	PE 18:2/20:4	M+H	C43H74NO8P	-0.563
814.5382	9.55	6.87E-05	6.26E-05	1.6	GM			5.65	PE 42:8	PE 20:2/22:6	M-H	C47H78NO8P	-1.243
740.5219	9.57	1.92E-07	3.91E-07	2.9	Control			7.72	PE 36:3	PE 18:1/18:2	M-H	C41H76NO8P	-2.246
772.5277	9.60	2.84E-06	3.88E-06	2.3	Control			10.06	PE O-40:8	PE P-18:1/22:6	M-H	C45H76NO7P	-1.255
764.5229	9.61	0.00236	0.001397	1.3	GM			2.75	PE 38:5		M-H	C43H76NO8P	-0.828
766.5385	9.72	9.79E-12	3.14E-10	6.4	GM			5.19	PE 38:4	PE 18:0/20:4	M-H	C43H78NO8P	-1.003
790.5386	9.72	0.000258	0.000195	1.5	GM			3.71	PE 40:6	PE 20:1/20:5	M-H	C45H78NO8P	-0.838
742.5370	9.75	3.22E-08	1.02E-07	3.6	Control			8.89	PE 36:2	PE 18:0/18:2	M-H	C41H78NO8P	-3.025
792.5539	9.85	3.79E-08	1.13E-07	3.1	GM			5.68	PE 40:5	PE 18:0/22:5	M-H	C45H80NO8P	-1.262
768.5539	9.86	2.12E-07	4.26E-07	2.1	Control			6.15	PE 38:3	PE 18:2/20:1	M-H	C43H80NO8P	-1.326
794.5691	9.99	1.39E-12	1.36E-10	7.2	GM			6.06	PE 40:4	PE 20:1/20:3	M-H	C45H82NO8P	-1.827
818.5696	9.99	1.83E-09	1.24E-08	5.3	GM			9.05	PE 42:6	PE 20:1/22:5	M-H	C47H82NO8P	-1.096
770.5692	10.03	3.24E-12	1.39E-10	6.3	Control			6.16	PE 38:2	PE 18:1/20:1	M-H	C43H82NO8P	-1.658
820.5840	10.12	2.13E-12	1.36E-10	7.0	GM			8.02	PE 42:5	PE 20:0/22:5|22:1/20:4	M-H	C47H84NO8P	-2.616
881.5178	10.27	7.53E-09	3.58E-08	3.5	Control			2.52	PI 38:6	PI 18:1/20:5	M-H	C47H79O13P	-0.798
855.5011	10.29	1.15E-08	5.08E-08	15.0	Control			24.68	PI 36:5		M-H	C45H77O13P	-2.088
798.6001	10.32	2.03E-11	5.21E-10	9.8	Control			12.04	PE 40:2	PE 18:1/22:1	M-H	C45H86NO8P	-2.118
883.5332	10.35	3.88E-09	2.13E-08	2.9	Control			8.51	PI 38:5	PI 18:1/20:4	M-H	C47H81O13P	-1.106
907.5329	10.35	1.83E-08	7.36E-08	2.6	Control			5.35	PI 40:7	PI 18:1/22:6	M-H	C49H81O13P	-1.403
857.5177	10.37	7.34E-05	6.64E-05	1.9	Control			16.31	PI 36:4	PI 16:0/20:4	M-H	C45H79O13P	-1.046
859.5324	10.48	3.98E-10	5.06E-09	13.0	Control			14.78	PI 36:3		M-H	C45H81O13P	-2.069
883.5338	10.48	1.4E-07	3.34E-07	6.8	Control			18.26	PI 38:5		M-H	C47H81O13P	-0.410
951.5196	10.48	2.15E-08	7.67E-08	3.7	Control			14.01	PI 40:8		M+FA-H	C49H79O13P	-4.849
885.5495	10.57	1.04E-06	1.63E-06	1.6	GM			5.58	PI 38:4	PI 18:0/20:4	M-H	C47H83O13P	-0.363
953.5369	10.57	1.01E-05	1.14E-05	1.5	GM			7.75	PI 40:7		M+FA-H		
861.5464	10.58	9.49E-10	7.89E-09	10.4	Control			13.33	PI 36:2		M-H	C45H83O13P	-3.986
911.5626	10.64	0.000134	0.000115	1.3	GM			6.66	PI 40:5	PI 18:0/22:5	M-H	C49H85O13P	-3.200
888.5664	10.70	1.12E-09	8.48E-09	4.0	Control			6.05	Hex2Cer 34:3		M-H		
913.5764	10.77	7.06E-06	8.24E-06	1.8	GM			11.74	PI 40:4	PI 18:0/22:4	M-H	C49H87O13P	-5.175
1543.9601	11.99	0.000918	0.000611	1.5	Control			12.66	CL 80:16		M-H	C89H142O17P2	-3.126
852.5539	15.16	0.001845	0.001012	1.7	GM			19.52	PC 42:11	PC 20:5/22:6	M+H	C50H78NO8P	0.132
568.3407	15.21	6.32E-05	5E-05	2.0	Control			13.54	LPC 22:6		M+H	C30H50NO7P	1.569
802.5379	15.30	2.98E-06	3.38E-06	3.7	GM			19.74	PC 38:8	PC 18:3/20:5	M+H	C46H76NO8P	-0.240
900.5750	15.30	3.97E-08	1.16E-07	5.0	GM			12.28	PC 42:9	PC 20:5/22:4	M+FA-H	C50H82NO8P	-1.217
806.4980	15.32	1.87E-05	1.92E-05	1.7	Control			6.52	PC 35:7	PC 15:2/20:5	M+FA-H		
828.5534	15.43	4.14E-05	3.52E-05	2.5	GM			17.52	PC 40:9	PC 20:4/20:5	M+H, M+Na	C48H78NO8P	-0.435
854.5682	15.45	1.11E-07	2.08E-07	5.6	GM			20.83	PC 42:10	PC 20:5/22:5	M+H, M+Na	C50H80NO8P	-1.447
752.5219	15.53	2.74E-05	2.56E-05	2.4	Control			13.1	PC 34:5	PC 14:0/20:5	M+H	C42H74NO8P	-0.786
544.3394	15.55	7.85E-06	7.82E-06	3.4	GM			12.38	LPC 20:4		M+H	C28H50NO7P	-0.674

824.5442	15.56	0.001714	0.001053	1.3	GM			5.12	PC 36:5	PC 16:0/20:5	M+FA-H	C44H78NO8P	-0.596
880.5833	15.56	1.23E-05	1.19E-05	2.6	GM			18.47	PC 44:11	PC 22:5/22:6	M+H, M+Na	C52H82NO8P	-2.065
804.5537	15.58	0.001555	0.000867	1.8	GM			17.15	PC 38:7	PC 18:2/20:5	M+H, M+Na	C46H78NO8P	-0.089
778.5386	15.66	5.77E-05	4.67E-05	2.0	Control			11.98	PC 36:6	PC 18:3/18:3	M+H, M+Na	C44H76NO8P	0.572
830.5688	15.71	0.005734	0.00274	1.6	GM			17.49	PC 40:8	PC 18:3/22:5	M+H, M+Na	C48H80NO8P	-0.803
918.5517	15.73	0.000656	0.000448	1.3	Control			6.61			Neg		
902.5897	15.77	0.001153	0.000745	1.6	GM			10.26	PC 42:8	PC 20:2/22:6	M+FA-H	C50H84NO8P	-2.315
894.5993	15.80	2.1E-09	1.14E-08	4.2	Control			11.56	PC 45:11		M+H		
882.5985	15.82	1.75E-08	5.2E-08	6.1	GM			19.7	PC 44:10	PC 22:5/22:5	M+H	C52H84NO8P	-2.555
818.5673	15.87	9.44E-06	9.33E-06	2.2	Control			12.99	PC 39:7	PC 17:1/22:6	M+H	C47H80NO8P	-2.650
792.5549	15.94	1.3E-06	1.69E-06	2.1	Control			9.93	PC 37:6		M+H	C45H78NO8P	1.408
776.5552	16.01	1.03E-10	1.5E-09	5.0	Control			10.22	PC O-37:7	PC P-15:0/22:6	M+H	C45H80NO8P	-4.656
832.5818	16.11	0.001192	0.000685	1.6	Control			10.95	PC 40:7	PC 18:2/22:5	M+H, M+Na	C48H82NO8P	-3.950
884.6119	16.12	5.04E-10	4.69E-09	6.1	GM			12.19	PC 44:9	PC 22:4/22:5	M+H	C52H86NO8P	-5.031
730.5371	16.13	2.5E-09	1.18E-08	4.2	Control			11.67	PC 32:2	PC 14:0/18:2	M+H	C40H76NO8P	-1.464
522.3556	16.15	7.31E-07	1.06E-06	3.7	Control			16.28	LPC 18:1		M+H, M+Na	C26H52NO7P	0.431
896.5740	16.16	3.09E-09	1.89E-08	2.0	Control			3.37	PC O-43:11		M+FA-H		
874.5950	16.19	1.99E-08	5.71E-08	4.6	Control			12.05	PC 42:8;O		M+H		
846.6018	16.20	3.68E-10	4.06E-09	2.4	Control			2.7	PC 41:7		M+H, M+Na	C49H84NO8P	1.267
806.5725	16.21	0.044216	0.015981	1.1	Control			7.67	PC 38:6	PC 18:2/20:4	M+H, M+Na, M+K	C46H80NO8P	-2.615
496.3402	16.27	0.000663	0.000406	1.7	Control			11.59	LPC 16:0		M+H, M+Na	C24H50NO7P	0.865
794.5681	16.29	1.98E-06	2.35E-06	1.9	Control			8.36	PC 37:5		M+H	C45H80NO8P	-1.724
900.6105	16.38	3.53E-09	1.4E-08	7.9	Control			12.58	PC 44:9;O		M+H, M+Na		
818.5718	16.39	1.11E-05	1.24E-05	1.4	Control			4.34			Neg	C47H82NO8P	1.544
782.5689	16.40	4.65E-06	4.89E-06	2.3	GM			14.13	PC 36:4	PC 18:2/18:2	M+H	C44H80NO8P	-0.738
790.5729	16.50	3.14E-07	4.87E-07	1.9	Control			9.2	PC O-38:7	PC P-16:0/22:6	M+H	C46H80NO7P	-2.051
820.5832	16.55	5.21E-08	1.15E-07	2.2	Control			6.52	PC 39:6	PC 17:0/22:6	M+H		
808.5841	16.63	0.006508	0.003077	1.4	GM			14.44	PC 38:5	PC 18:1/20:4	M+H	C46H82NO8P	-1.276
784.5835	16.65	7.18E-05	5.65E-05	1.8	Control			14.81	PC 36:3	PC 18:1/18:2	M+H	C44H82NO8P	-2.039
898.5875	16.66	1.75E-10	2.8E-09	3.9	Control			6.6	PC O-43:10		M+FA-H		
848.6152	16.66	2.27E-09	1.18E-08	5.1	Control			12.42	PC 41:6		M+H, M+Na	C49H86NO8P	-1.390
732.5520	16.68	3.81E-09	1.46E-08	3.7	Control			10.49	PC 32:1	PC 14:0/18:1	M+H	C40H78NO8P	-2.424
860.6147	16.76	0.002925	0.001491	1.6	Control			20.08	PC 42:7	PC 20:1/22:6	M+H, M+Na	C50H86NO8P	-1.900
810.5980	16.78	0.000321	0.000214	1.6	GM			13.73	PC 38:4	PC 18:2/20:2	M+H	C46H84NO8P	-3.342
758.5688	16.81	3.51E-06	3.81E-06	2.1	Control			12.92	PC 34:2	PC 16:0/18:2	M+H	C42H80NO8P	-0.836
760.5749	16.81	7.73E-07	1.1E-06	2.2	Control			11.34	PC 34:1	PC 16:0/18:1	M+H	C42H82NO8P	-13.454
792.5881	16.81	0.001096	0.000636	1.6	Control			12.76	PC O-38:6	PC P-18:0/20:5	M+H	C46H82NO7P	-2.635
772.5833	16.88	1.59E-09	9.57E-09	3.1	Control			8.81	PC 35:2		M+H	C43H82NO8P	-2.261
852.6124	16.93	3.87E-12	1.68E-10	7.3	Control			7.52	PC 40:5;O		M+H		
818.6043	17.00	2.6E-06	2.97E-06	2.1	Control			9.3	PC O-40:7	PC P-18:0/22:6	M+H	C48H84NO7P	-1.815
766.5718	17.04	0.0003	0.000206	1.8	Control			11.95	PC O-36:5	PC O-16:0/20:5	M+H, M+Na	C41H80NO8P	-1.534
798.5962	17.10	1.23E-07	2.25E-07	2.1	Control			6.96	PC 37:3		M+H	C45H84NO8P	-5.682
786.5996	17.22	2.59E-08	6.85E-08	6.0	Control			18.98	PC 36:2	PC 18:1/18:1	M+H	C44H84NO8P	-1.459
836.6150	17.27	3.77E-05	3.3E-05	2.0	GM			15.81	PC 40:5	PC 18:0/22:5	M+H	C48H86NO8P	-1.647
760.5852	17.39	3.21E-05	2.87E-05	1.7	Control			12.86	PC 34:1		M+H, M+Na	C42H82NO8P	-1.032
812.6157	17.39	8.87E-07	1.21E-06	2.3	Control			13.05	PC 38:3	PC 18:2/20:1	M+H	C46H86NO8P	-0.877
888.6401	17.39	1.3E-07	2.33E-07	2.5	Control			6	PC 44:7	PC 22:1/22:6	M+H	C52H90NO8P	-8.540
774.5991	17.45	1.04E-07	1.98E-07	4.9	Control			11.34	PC 35:1		M+H	C43H84NO8P	-2.075
854.6264	17.48	3.05E-11	5.69E-10	7.6	Control			8.43	PC 40:4;O		M+H		
800.6162	17.54	2.91E-08	7.44E-08	3.1	Control			8.75	PC 37:2		M+H	C45H86NO8P	-0.231
786.6002	17.59	1.47E-06	1.81E-06	1.9	Control			9.2	PC 36:2		M+H	C44H84NO8P	-0.622
838.6291	17.63	1.46E-06	1.81E-06	2.2	GM			13.24	PC 40:4	PC 20:1/20:3	M+H	C48H88NO8P	-3.531
862.6309	17.65	3.42E-06	3.75E-06	2.3	GM			13.84	PC 42:6	PC 20:1/22:5	M+H	C50H88NO8P	-1.344
814.6309	18.04	1.03E-09	7.93E-09	2.6	Control			6.93	PC 38:2	PC 18:1/20:1	M+H	C46H88NO8P	-1.356
864.6453	18.09	4.46E-09	1.62E-08	3.0	GM			8.58	PC 42:5	PC 20:0/22:5	M+H	C50H90NO8P	-2.798
802.6308	18.17	3.51E-08	8.49E-08	6.3	Control			14.99	PC 37:1		M+H	C45H88NO8P	-1.542
776.6138	18.21	6.95E-10	6.05E-09	6.8	Control			3.4	PC 35:0		M+H	C43H86NO8P	-3.389
840.6455	18.21	1.27E-07	2.3E-07	2.0	Control			5.93	PC 40:3	PC 20:1/20:2	M+H	C48H90NO8P	-2.641
788.6157	18.25	4.89E-07	7.34E-07	1.7	Control			6.29	PC 36:1	PC 18:0/18:1	M+H	C44H86NO8P	-0.896
842.6630	18.96	1.03E-09	7.93E-09	3.8	Control			7.97	PC 40:2	PC 18:1/22:1	M+H	C48H92NO8P	-0.454
