# Supplementary material for: Profiling Phospholipids within Atlantic Salmon Salmo salar with Regards to a Novel Terrestrial Omega-3 Oil Source
Source: Metabolites. 2022 Sep 10;12(9):851. doi: 10.3390/metabo12090851 (PMC9503986; doi:10.3390/metabo12090851)
Supplement: Supplementary file 1 [file metabolites-12-00851-s001.zip › Supplementary Tables S11-S20 LC-MS.pdf]

Supplementary Table S11. LC-MS/MS data for brain tissue at the final timepoint. Green and red represent high and low absolute peak areas, respectively. $p < 0.01$ = **, $p = 0.01$-0.05 = *. Stars with a red background denote falling below the Benjamini-Hochberg critical value.

MW	Simple ID	ID 1	ID2		Cont	GM	Fold	Highest	RSD%	RSD%	p		Cont	GM	RSD%	RSD%	p
714.5	PE 34:2	PE 16:1/18:1		Area			1.3	Control	32.8	14.7		%	67.1	61.3	13.1	5.4	
		PE 16:0/18:2		Area			1.1	GM	8.6	6.0		%	32.9	38.7	26.8	8.5	
				total			1.1	Control	20.7	10.3							
738.5	PE 36:4	PE 18:1/18:3		Area			2.3	GM	6.6	6.6	**	%	24.9	27.6	17.4	7.1	
		PE 16:0/20:4		Area			1.9	GM	18.1	11.6	**	%	75.1	72.4	5.8	2.7	
				total			2.0	GM	12.1	9.6	**						
742.5	PE 36:2	PE 18:1/18:1		Area			1.1	Control	20.3	7.1		%	93.5	92.4	0.5	1.5	
		PE 18:2/18:0		Area			1.0	GM	19.4	13.6		%	6.5	7.6	7.8	18.9	
				total			1.1	Control	20.1	5.8							
746.5	PE O-38:7	PE P-18:1/20:5		Area			1.1	GM	17.7	7.8		%	85.0	75.7	1.5	3.4	**
		PE P-16:0/22:6		Area			2.0	GM	16.4	13.4	**	%	15.0	24.3	8.3	10.6	**
				total			1.3	GM	17.2	7.3							
760.5	PE 38:7	PE 18:2/20:5		Area			1.9	GM	11.6	8.0	**	%	42.4	59.9	3.8	5.1	**
		PE 16:1/22:6		Area			1.1	Control	6.7	15.7		%	57.6	40.1	2.8	7.6	**
				total			1.3	GM	8.5	9.9	*						
766.5	PE 38:4	PE 18:1/20:3		Area			2.1	GM	14.7	8.3	**	%	42.0	37.3	12.5	12.6	
		PE 18:0/20:4		Area			2.5	GM	28.4	21.6	*	%	58.0	62.7	9.1	7.5	
				total			2.3	GM	21.2	15.6	**						
768.6	PE 38:3	PE 18:1/20:2		Area			1.4	GM	18.6	9.0	*	%	36.3	41.3	10.2	2.5	
		PE 16:1/22:2		Area			1.1	GM	7.3	12.9		%	63.7	58.7	5.8	1.8	
				total			1.2	GM	10.3	11.3							
774.5	PE O-40:7	PE P-18:1/22:5		Area			1.1	GM	8.1	9.7		%	47.3	41.8	19.7	16.1	
		PE P-18:0/22:6		Area			1.3	GM	43.3	19.3		%	52.7	58.2	17.7	11.6	
				total			1.2	GM	27.3	9.6							
784.5	PE 40:9	PE 20:4/20:5		Area			1.5	GM	44.4	56.1		%	35.5	18.4	33.1	48.2	
		PE 18:3/22:6		Area			3.6	GM	10.0	5.0	**	%	64.5	81.6	18.2	10.9	
				total			2.8	GM	14.7	7.9	**						
786.5	PE 40:8	PE 20:3/20:5		Area			3.1	GM	13.0	15.0	**	%	25.7	42.8	13.6	8.7	**
		PE 18:2/22:6		Area			1.4	GM	5.6	1.8	**	%	74.3	57.2	4.7	6.5	**
				total			1.8	GM	1.4	6.8	**						
788.5	PE 40:7	PE 20:2/20:5		Area			1.7	GM	10.2	5.4	**	%	3.4	6.0	23.4	14.1	*
		PE 18:1/22:6		Area			1.1	Control	20.8	12.5		%	96.6	94.0	0.8	0.9	*
				total			1.1	Control	20.0	11.7							
802.6	PC 34:2	PC 16:1/18:1		Area			1.4	Control	9.8	11.0	*	%	30.7	18.2	4.1	4.7	**
		PC 16:0/18:2 (a)		Area			1.4	GM	6.9	4.0	**	%	43.4	53.0	4.9	8.4	*
		PC 16:0/18:2 (b)		Area			1.3	GM	9.0	20.5		%	26.0	28.8	8.8	14.2	
				Total			1.2	GM	5.9	6.8	*	Ratio a/b	1.7	1.9	13.8	22.1	
810.5	PE 42:10	PE 20:5/22:5		Area			1.1	Control	30.5	27.3		%	30.8	14.9	28.6	32.6	
		PE 20:4/22:6 (a)		Area			2.7	GM	26.2	18.4	**	%	37.0	51.4	25.8	8.0	
		PE 20:4/22:6 (b)		Area			2.0	GM	17.6	11.6	**	%	32.2	33.8	7.9	4.4	
				Total			1.9	GM	10.2	10.4	**	Ratio a/b	1.2	1.5	30.1	7.5	
822.5	PC 36:6	PC 16:1/20:5		Area			2.1	Control	32.8	33.9		%	30.7	28.9	19.6	21.1	
		PC 14:0/22:6		Area			2.0	Control	27.0	12.1	*	%	69.3	71.1	8.7	8.6	
				total			2.1	Control	25.6	15.1	*						
826.6	PC 36:4	PC 18:3/18:1		Area			1.6	GM	31.5	9.2	*	%	14.3	11.9	22.0	21.1	
		PC 16:0/20:4		Area			2.1	GM	12.5	16.9	**	%	85.7	88.1	3.7	2.9	
				total			2.0	GM	13.6	14.5	**						
885.6	PI 38:4	PI 18:0/20:4 (a)		Area			1.2	GM	70.1	82.4		%	66.7	55.8	30.6	34.5	
		PI 18:0/20:4 (b)		Area			2.2	GM	18.9	20.6	*	%	33.3	44.2	61.5	43.4	
				total			1.5	GM	56.2	50.7		Ratio a/b	2.7	1.7	66.7	89.7	
910.6	PC 42:4	PC 18:1/24:3	PC 22:3/20:1	Area			2.4	GM	3.0	8.8	**	%	26.8	32.4	1.5	0.1	**
		PC 18:3/24:1		Area			1.8	GM	3.0	8.7	**	%	73.2	67.6	0.6	0.1	**
				total			2.0	GM	2.8	8.7	**						
912.7	PC 42:3	PC 18:1/24:2		Area			1.3	GM	8.6	9.5	*	%	88.5	88.4	2.8	1.6	
		PC 18:2/24:1		Area			1.3	GM	30.2	12.1		%	11.5	11.6	21.2	12.4	
				total			1.3	GM	10.9	8.7	*						
932.6	PC 44:7	PC 20:5/24:2		Area			1.6	GM	29.0	17.2		%	52.2	69.5	20.9	6.8	
		PC 22:6/22:1		Area			1.3	Control	13.2	5.4	*	%	47.8	30.5	22.9	15.4	
				total			1.2	GM	10.4	11.2							
936.7	PC 44:5	PC 20:4/24:1 (a)		Area			2.0	GM	10.1	20.3	*	%	25.8	27.4	12.1	10.0	
		PC 20:4/24:1 (b)		Area			2.0	GM	24.7	13.8	**	%	52.9	59.8	7.0	2.4	*
		PC 20:5/24:0		Area			1.1	GM	19.8	5.3		%	21.3	12.8	11.2	14.3	**
				Total			1.8	GM	18.7	13.4	**	Ratio a/b	0.5	0.5	18.7	12.0	
938.7	PC 44:4	PC 20:3/24:1		Area			1.8	GM	21.7	16.2	*	%	93.3	89.5	0.9	2.1	*
		PC 20:4/24:0		Area			2.9	GM	30.6	36.5	*	%	6.7	10.5	12.6	17.7	*
				total			1.9	GM	22.1	18.4	*						
940.7	PC 44:3	PC 18:1/26:2	PC 20:2/C24:1 (a)	Area			1.4	GM	7.2	27.7		%	36.0	38.7	14.0	16.2	
		PC 20:2/24:1 (b)	PC 18:2/26:1	Area			1.2	GM	19.1	11.1		%	64.0	61.3	7.9	10.2	
				total			1.3	GM	12.5	15.5		Ratio b/a	1.8	1.6	21.2	28.2	
962.7	PC 46:6	PC 24:1/22:5		Area			1.3	GM	16.9	8.3		%	77.6	80.6	3.5	1.6	
		PC 20:5/26:2		Area			1.0	GM	31.3	8.2		%	22.4	19.4	12.2	6.8	
				total			1.2	GM	20.2	7.6							
964.7	PC 46:5	PC 22:4/24:1	PC 20:4/26:1 (a)	Area			2.7	GM	29.7	12.5	**	%	54.6	64.1	8.2	1.1	*
		PC 22:5/24:0	PC 20:4/26:1 (b)	Area			1.9	GM	17.1	11.4	**	%	45.4	35.9	9.9	1.9	*
				total			2.3	GM	23.7	12.1	**	Ratio a/b	1.2	1.8	17.4	2.9	*

Supplementary Table S12. LC-MS/MS data for brain tissue at the intermediate timepoint. Green and red represent high and low absolute peak areas, respectively. p < 0.01 = **, p= 0.01-0.05 = *. Stars with a red background denote falling below the Benjamini-Hochberg critical value.

MW	Simple ID	ID 1	ID 2	ID 3		Control	GM	Fold	Highest	RSD%	RSD%	p		Control	GM	RSD%	RSD%	p
750.5	PE O-38:5	PE P-18:0/20:4 (a)			Area			1.6	GM	33.0	33.2		%	**32.7**	**34.4**	6.0	12.8	
		PE P-18:0/20:4 (b)			Area			1.4	GM	37.2	21.7		%	**67.3**	**65.6**	2.9	6.7	
					Total			1.5	GM	35.6	25.1		Ratio b/a	**2.1**	**1.9**	9.2	20.0	
766.5	PE 38:4	PE 18:1/20:3			Area			1.7	GM	23.3	15.7	*	%	**36.9**	**37.3**	15.7	8.3	
		PE 18:0/20:4			Area			1.7	GM	20.3	26.8		%	**63.1**	**62.7**	9.2	4.9	
					Total			1.7	GM	16.9	22.7	*						
784.5	PE 40:9	PE 20:4/20:5			Area			2.1	GM	48.8	29.1		%	**12.5**	**16.4**	43.6	13.1	
		PE 18:3/22:6			Area			1.5	GM	32.4	29.2		%	**87.5**	**83.6**	6.2	2.6	
					Total			1.5	GM	30.2	28.4							
786.5	PE 40:8	PE 20:5/20:3			Area			1.9	GM	31.2	18.7	*	%	**20.2**	**31.5**	5.6	4.2	**
		PE 18:2/22:6			Area			1.1	GM	26.5	20.0		%	**79.8**	**68.5**	1.4	2.0	**
					Total			1.2	GM	27.4	19.3							
788.5	PE 40:7	PE 20:2/20:5			Area			1.6	GM	19.3	14.7	*	%	**1.7**	**4.3**	25.6	28.8	*
		PE 18:1/22:6 (a)			Area			1.7	Control	37.0	13.4		%	**94.3**	**91.6**	1.7	0.2	*
		PE 18:1/22:6 (b)			Area			1.7	Control	74.4	37.0		%	**4.0**	**4.1**	51.3	26.7	
					Total			1.6	Control	38.3	13.3		Ratio a/b	**30.5**	**23.8**	66.4	31.3	
794.6	PE 40:4	PE 18:1/22:3	PE 20:3/20:1		Area			1.5	GM	20.4	54.7		%	**40.9**	**39.6**	7.1	22.2	
		PE 18:0/22:4			Area			1.4	GM	32.2	27.5		%	**59.1**	**60.4**	4.9	14.6	
					Total			1.5	GM	27.3	37.6							
810.5	PE 42:10	PE 20:5/22:5			Area			1.1	Control	20.7	31.5		%	**22.6**	**14.5**	12.4	9.4	*
		PE 20:4/22:6 (a)			Area			1.4	GM	37.7	31.3		%	**47.8**	**49.4**	9.1	8.2	
		PE 20:4/22:6 (b)			Area			1.7	GM	27.7	14.1	*	%	**29.6**	**36.1**	6.8	14.8	
					Total			1.4	GM	30.8	23.7		Ratio a/b	**1.6**	**1.4**	15.0	24.7	
814.5	PE 42:8	PE 20:2/22:6 (a)			Area			1.1	Control	34.0	20.1		%	**64.6**	**65.2**	15.7	6.4	
		PE 20:2/22:6 (b)			Area			1.3	Control	64.4	6.7		%	**35.4**	**34.8**	28.7	11.9	
					Total			1.1	Control	45.6	14.3		Ratio a/b	**2.0**	**1.9**	49.8	17.7	
822.5	PC 36:6	PC 16:1/20:5			Area			2.1	Control	22.5	3.0	*	%	**22.1**	**24.0**	15.4	33.5	
		PC 14:0/22:6			Area			2.1	Control	24.7	46.9		%	**77.9**	**76.0**	4.4	10.6	
					Total			2.1	Control	22.5	37.1	*						
874.6	PC 40:8	PC 20:3/20:5	PC 20:4/20:4		Area			2.1	GM	36.6	18.4	*	%	**14.6**	**28.7**	32.3	13.6	*
		PC 18:2/22:6			Area			1.1	Control	2.2	9.4		%	**85.4**	**71.3**	5.5	5.5	*
					Total			1.1	GM	6.2	9.6							
876.6	PC 40:7	PC 18:1/22:6 (a)			Area			1.4	Control	19.8	19.6		%	**63.0**	**73.9**	2.0	1.2	**
		PC 18:1/22:6 (b)			Area			2.3	Control	18.8	16.4	**	%	**37.0**	**26.1**	3.4	3.3	**
					Total			1.6	Control	19.2	18.7	*	Ratio b/a	**0.6**	**0.4**	5.4	4.5	**
882.6	PC 40:4	PC 20:0/20:4			Area			1.1	Control	23.4	20.7		%	**53.7**	**34.9**	10.1	1.3	**
		PC 18:1/22:3	PC 20:3/20:1	PC 20:2/20:2	Area			2.0	GM	35.8	19.7	*	%	**46.3**	**65.1**	11.7	0.7	**
					Total			1.4	GM	27.3	20.1							
898.6	PC 42:10	PC 20:5/22:5			Area			1.4	GM	10.9	25.9		%	**17.7**	**14.1**	23.6	20.7	
		PC 20:4/22:6 (a)			Area			1.5	GM	15.8	24.3		%	**42.6**	**37.7**	6.8	5.6	
		PC 20:4/22:6 (b)			Area			2.0	GM	26.0	21.4	*	%	**39.8**	**48.1**	12.7	8.4	
					Total			1.7	GM	14.2	20.3	*	Ratio a/b	**1.1**	**0.8**	17.4	13.6	
910.6	PC 42:4	PC 20:1/22:3			Area			1.5	GM	31.5	23.8		%	**41.7**	**39.6**	12.0	18.9	
		PC 18:3/24:1			Area			1.5	GM	53.8	7.3		%	**58.3**	**60.4**	8.6	12.4	
					Total			1.5	GM	44.8	5.4							
936.7	PC 44:5	PC 20:4/24:1 (a)			Area			1.1	GM	36.7	14.4		%	**40.4**	**33.9**	13.5	16.2	
		PC 20:4/24:1 (b)			Area			2.0	GM	32.2	25.8	*	%	**29.2**	**43.0**	10.3	24.6	
		PC 20:5/24:0			Area			1.1	GM	33.3	23.5		%	**30.4**	**23.2**	27.2	23.3	
					Total			1.4	GM	28.3	3.7		Ratio a/b	**1.4**	**0.8**	6.2	36.7	*
964.7	PC 46:5	PC 22:4/24:1	PC 20:4/26:1		Area			1.6	GM	53.9	12.1		%	**52.2**	**69.0**	8.2	4.2	**
		PC 22:5/24:0			Area			1.2	Control	40.9	1.9		%	**47.8**	**31.0**	8.9	9.3	**
					Total			1.3	GM	47.5	8.0							

Supplementary Table S13. LC-MS/MS data for eye tissue at the final timepoint. Green and red represent high and low absolute peak areas, respectively. p < 0.01 = **, p= 0.01-0.05 = *. Stars with a red background denote falling below the Benjamini-Hochberg critical value.

MW	Simple ID	ID1	ID2		Control	GM	Fold	Highest	RSD%	RSD%	p		Control	GM	RSD%	RSD%	p
722.5	PE O-36:5	PE O-18:3/18:2		Area			3.6	GM	11.6	28.8	*	%	44.8	49.7	19.8	9.5	
		PE P-16:0/20:4		Area			2.9	GM	25.3	13.4	**	%	55.2	50.3	16.1	9.4	
				Total			3.2	GM	11.5	20.7	**						
726.6	PE O-36:3	PE O-18:2/18:1		Area			1.3	Control	15.8	25.6		%	94.7	92.0	1.3	1.3	
		PE P-18:0/18:2		Area			1.2	GM	10.6	13.9		%	5.3	8.0	23.8	15.1	
				Total			1.3	Control	14.6	24.4							
742.5	PE 36:2	PE 18:1/18:1		Area			1.3	Control	18.7	18.0		%	92.7	86.5	1.0	3.0	*
		PE 18:0/18:2	PE 16:0/20:2	Area			1.5	GM	24.2	24.6		%	7.3	13.5	13.4	19.1	*
				Total			1.2	Control	18.8	17.2							
746.5	PE O-38:7	PE O-18:3/20:4		Area			7.0	GM	27.9	7.2	**	%	2.5	17.2	17.0	6.8	**
		PE O-18:2/20:5		Area			1.3	Control	15.2	10.1		%	76.5	59.6	1.9	3.8	**
		PE P-16:0/22:6		Area			1.1	GM	17.2	26.6		%	21.0	23.2	5.1	14.8	
				Total			1.0	GM	15.5	13.4							
750.5	PE O-38:5	PE O-18:2/20:3		Area			1.5	GM	22.9	22.4		%	48.8	31.0	6.3	15.2	**
		PE P-18:0/20:4		Area			3.1	GM	24.4	8.4	**	%	51.2	69.0	6.0	6.8	**
				Total			2.3	GM	22.7	10.0	**						
760.5	PE 38:7	PE 18:3/20:4	PE 18:2/20:5	Area			1.6	GM	19.9	17.0	*	%	78.0	86.1	4.5	3.5	*
		PE 16:1/22:6		Area			1.1	Control	34.7	26.6		%	22.0	13.9	15.8	21.5	*
				Total			1.4	GM	22.4	16.6							
762.6	PE 38:6	PE 18:2/20:4		Area			3.2	GM	10.2	4.7	**	%	5.6	15.5	25.0	8.5	**
		PE 18:1/20:5		Area			1.5	Control	20.3	25.4		%	34.0	19.2	10.4	13.8	**
		PE 16:0/22:6		Area			1.2	GM	15.6	12.8		%	60.4	65.3	6.0	3.0	
				Total			1.1	GM	14.1	13.3							
774.5	PE O-40:7	PE P-18:1/22:5		Area			1.2	Control	18.9	23.0		%	35.4	28.0	4.8	23.5	
		PE P-18:0/22:6		Area			1.2	GM	18.8	10.8		%	64.6	72.0	2.6	9.1	
				Total			1.1	GM	18.4	5.0							
776.5	PC 32:1	PC 16:0/16:1 (a)	PC 14:0/18:1	Area			1.8	Control	3.2	17.2	**	%	74.5	58.1	1.9	6.1	**
		PC 16:0/16:1 (b)		Area			1.0	GM	12.8	12.5		%	21.1	30.0	9.4	9.9	*
		PC 16:0/16:1 (c)		Area			2.0	GM	11.2	16.4	**	%	4.3	11.9	15.2	6.0	**
				Total			1.4	Control	4.0	14.0	*						
784.5	PE 40:9	PE 20:4/20:5		Area			1.1	GM	6.0	13.3		%	14.9	6.5	19.5	20.4	*
		PE 18:3/22:6		Area			2.8	GM	22.4	14.2	**	%	85.1	93.5	3.4	1.4	*
				Total			2.5	GM	19.6	12.9	**						
786.5	PE 40:8	PE 20:4/20:4	PE 20:3/20:5	Area			3.1	GM	18.4	25.6	*	%	6.7	11.4	26.0	15.1	*
		PE 18:2/22:6		Area			1.7	GM	12.3	10.7	**	%	93.3	88.6	1.9	2.0	*
				Total			1.8	GM	10.5	12.2	**						
788.5	PE 40:7	PE 20:2/20:5		Area			1.4	GM	3.4	21.8		%	4.5	7.2	16.6	8.4	**
		PE 18:1/22:6		Area			1.2	Control	18.7	13.8		%	95.5	92.8	0.8	0.7	**
				Total			1.2	Control	18.0	14.3							
792.6	PE 40:5	PE 20:4/20:1	PE 18:1/22:4	Area			1.0	GM	31.5	25.5		%	37.6	26.4	8.3	11.6	*
		PE 18:0/22:5		Area			1.7	GM	23.3	20.0	*	%	62.4	73.6	5.0	4.2	*
				Total			1.4	GM	25.9	20.7							
810.5	PE 42:10	PE 20:4/22:6 (a)		Area			2.7	GM	22.6	11.5	**	%	62.6	63.3	7.1	2.7	
		PE 20:4/22:6 (b)		Area			2.6	GM	13.0	16.5	**	%	37.4	36.7	11.9	4.7	
				Total			2.7	GM	17.6	13.0	**	Ratio a/b	1.7	1.7	19.4	7.3	
814.5	PE 42:8	PE 20:2/22:6 (a)		Area			1.5	GM	29.7	19.4		%	86.9	86.1	2.2	1.3	
		PE 20:2/22:6 (b)		Area			1.5	GM	45.2	22.7		%	13.1	13.9	14.3	8.2	
				Total			1.5	GM	31.8	19.6		Ratio a/b	6.7	6.2	16.5	9.1	
818.6	PE 42:6	PE 20:1/22:5		Area			1.4	GM	20.4	37.7		%	19.5	23.0	16.4	19.7	
		PE 20:0/22:6		Area			1.1	GM	29.3	14.0		%	80.5	77.0	4.0	5.9	
				Total			1.1	GM	26.5	18.7							
822.5	PC 36:6	PC 18:3/18:3		Area			4.8	GM	15.6	19.0	**	%	4.5	44.5	16.1	30.0	**
		PC 16:1/20:5		Area			4.4	Control	36.5	34.5	*	%	42.2	20.1	10.3	20.0	**
		PC 14:0/22:6		Area			3.0	Control	26.2	41.8	*	%	53.3	35.4	7.0	26.8	*
				Total			2.1	Control	29.8	13.9	*						
826.6	PC 36:4	PC 18:2/18:2	PC 16:1/20:3	Area			1.3	GM	4.2	9.6	*	%	12.4	7.9	5.0	7.2	**
		PC 18:1/18:3		Area			1.3	GM	6.1	16.2		%	26.6	16.3	3.2	2.7	**
		PC 16:0/20:4 (a)		Area			1.4	GM	8.2	9.7	*	%	9.4	6.2	3.4	4.1	**
		PC 16:0/20:4 (b)		Area			2.9	GM	4.7	14.1	**	%	51.6	69.6	0.7	0.9	**
				total			2.1	GM	4.7	13.7	**	Ratio b/a	5.5	11.2	3.5	4.5	**
828.6	PC 36:3	PC 18:1/18:2		Area			1.8	Control	3.9	14.6	**	%	62.8	31.4	2.1	12.5	**
		PC 16:0/20:3 (a)		Area			3.7	GM	20.9	46.8		%	5.6	17.6	14.8	37.1	*
		PC 16:0/20:3 (c)		Area			1.5	GM	14.1	9.4	*	%	25.2	34.0	8.2	13.5	*
		PC 18:0/18:3		Area			3.1	GM	27.8	23.4	**	%	6.4	17.0	30.9	10.8	**
				total			1.2	GM	5.9	14.3		Ratio c/a	4.6	2.2	6.4	41.9	*
830.6	PC 36:2	PC 18:1/18:1		Area			1.6	Control	10.5	12.4	**	%	68.5	44.8	3.1	10.3	**
		PC 16:0/20:2 (a)		Area			1.4	GM	13.4	12.9	*	%	11.0	15.2	13.8	9.2	*
		PC 16:0/20:2 (b)	PC 18:0/18:2	Area			1.9	GM	16.9	15.9	*	%	20.6	40.0	6.2	8.5	**
				Total			1.0	Control	11.0	9.8		Ratio b/a	1.9	2.6	13.4	5.0	*
836.6	PC O-38:6	PC P-18:0/20:5		Area			2.2	Control	11.6	14.5	**	%	68.0	52.5	9.8	2.4	*
		PC O-16:0/22:6		Area			1.2	Control	31.3	18.4		%	32.0	47.5	20.7	2.7	*
				Total			1.7	Control	14.1	16.3	*						
854.6	PC 38:4	PC 16:0/22:4		Area			2.4	GM	20.5	3.8	**	%	14.4	16.4	11.6	14.4	
		PC 18:0/20:4		Area			2.1	GM	14.6	22.3	*	%	85.6	83.6	2.0	2.8	
				Total			2.2	GM	15.0	19.3	*						
872.5	PC 40:9	PC 20:4/20:5		Area			1.5	GM	33.6	8.1		%	31.8	17.9	6.6	12.2	**
		PC 18:3/22:6		Area			3.3	GM	28.4	7.1	**	%	68.2	82.1	3.0	2.6	**
				Total			2.7	GM	29.8	4.4	**						
874.6	PC 40:8	PC 18:3/22:5		Area			3.4	GM	38.3	9.6	**	%	9.7	16.3	44.0	15.6	
		PC 20:4/20:4	PC 20:5/20:3	Area			2.9	GM	15.1	21.0	**	%	17.8	25.3	17.3	8.8	*
		PC 18:2/22:6		Area			1.6	GM	34.5	26.2		%	72.5	58.4	8.5	4.7	*
				Total			2.0	GM	28.5	21.4	*						
876.6	PC 40:7	PC 18:2/22:5		Area			2.1	GM	34.3	21.6	*	%	6.7	15.7	16.3	0.8	**
		PC 20:5/20:2		Area			1.7	GM	28.0	20.6		%	6.8	13.1	12.0	8.3	**
		PC 18:1/22:6 (a)		Area			1.1	GM	34.9	15.8		%	27.4	33.3	15.8	10.3	
		PC 18:1/22:6 (b)		Area			1.7	Control	19.2	32.0		%	59.1	37.9	8.3	11.7	**
				Total			1.1	Control	23.0	22.2		Ratio b/a	2.2	1.2	23.5	22.1	*
880.6	PC 40:5	PC 18:0/22:5	C20:1/20:4	Area			1.1	GM	23.0	23.0		%	64.2	53.7	3.4	6.9	*
		PC 20:0/20:5		Area			1.7	GM	32.7	12.5	*	%	35.8	46.3	6.2	8.0	*
				Total			1.3	GM	26.53	17.71							
898.6	PC 42:10	PC 20:5/22:5		Area			1.9	GM	55.3	21.6		%	20.2	16.3	31.2	21.4	
		PC 20:4/22:6 (a)		Area			2.3	GM	30.6	26.7	*	%	41.0	36.4	6.6	7.5	
		PC 20:4/22:6 (b)		Area			3.2	GM	33.1	26.1	*	%	38.8	47.3	10.5	3.0	*
				Total			2.5	GM	35.2	23.2	*	Ratio a/b	1.1	0.8	7.65	7.15	**

Supplementary Table S14. LC-MS/MS data for eye tissue at the intermediate timepoint. Green and red represent high and low absolute peak areas, respectively. $p < 0.01$ = **, p= 0.01-0.05 = *. Stars with a red background denote falling below the Benjamini-Hochberg critical value.

MW	Simple ID	ID1	ID2		Control	GM	Fold	Highest	RSD%	RSD%	P		Control	GM	RSD%	RSD%	P
742.5	PE 36:2	PE 18:1/18:1		Area			3.5	Control	17.3	26.3	**	%	91.4	78.6	1.5	8.8	*
		PE 18:0/18:2		Area			1.2	Control	4.6	11.7	*	%	8.6	21.4	15.8	32.4	*
				Total			3.0	Control	15.8	18.7	**						
748.5	PE O-38:6	PE P-18:1/20:4		Area			1.3	GM	10.6	6.0	*	%	61.8	72.7	12.7	1.3	
		PE P-18:0/20:5		Area			1.3	Control	25.1	5.9		%	38.2	27.3	20.6	3.3	
				Total			1.1	GM	7.4	5.7							
762.6	PE 38:6	PE 18:2/20:4		Area			3.5	GM	9.8	23.7	**	%	3.7	20.6	23.6	13.9	**
		PE 18:1/20:5		Area			4.4	Control	9.0	14.4	**	%	31.0	11.7	7.9	15.3	**
		PE 16:0/22:6		Area			1.6	Control	16.8	9.5	*	%	65.4	67.7	4.7	5.4	
				Total			1.6	Control	12.7	10.5	**						
764.5	PE 38:5	PE 18:1/20:4	PE 16:0/22:5	Area			1.0	GM	15.4	9.9		%	42.1	54.3	13.9	6.9	*
		PE 18:0/20:5		Area			1.6	Control	21.1	11.3	*	%	57.9	45.7	10.1	8.2	*
				Total			1.3	Control	14.0	7.5							
776.6	PC 32:1	PC 16:0/16:1 (a)	PC 14:0/18:1	Area			2.2	Control	16.7	19.6	**	%	66.0	57.5	4.0	2.4	**
		PC 16:0/16:1 (b)		Area			1.8	Control	19.3	15.0	*	%	27.2	28.3	4.0	4.5	
		PC 16:1/16:0 (c)		Area			1.1	GM	29.4	22.1		%	6.8	14.2	40.1	4.5	**
				Total			1.9	Control	15.4	18.6	*						
794.6	PE 40:4	PE 18:0/22:4		Area			1.6	GM	6.8	10.9	**	%	78.2	72.7	14.4	10.1	
		PE 20:0/20:4		Area			2.1	GM	55.4	26.0		%	21.8	27.3	51.7	26.9	
				Total			1.7	GM	7.3	0.9	**						
810.5	PE 42:10	PE 20:4/22:6 (a)		Area			1.2	GM	23.7	20.3		%	59.4	57.2	7.7	3.8	
		PE 20:4/22:6 (b)		Area			1.4	GM	17.6	18.6		%	40.6	42.8	11.3	5.1	
				Total			1.3	GM	19.7	19.2		Ratio a/b	1.5	1.3	19.2	8.9	
818.6	PE 42:6	PE 20:1/22:5		Area			1.6	Control	14.2	29.4	*	%	14.1	12.2	6.2	16.3	
		PE 20:0/22:6		Area			1.4	Control	9.2	19.2	*	%	85.9	87.8	1.0	2.3	
				Total			1.4	Control	9.8	20.0	*						
822.5	PC 36:6	PC 16:1/20:5 (a)		Area			3.2	Control	17.8	19.0	**	%	26.9	24.4	12.4	14.2	
		PC 16:1/20:5 (b)		Area			2.2	Control	36.5	33.8		%	13.8	19.9	25.0	45.4	
		PC 14:0/22:6		Area			3.1	Control	22.5	28.1	**	%	59.3	55.6	10.1	18.0	
				Total			3.0	Control	20.2	12.1	**	Ratio a/b	2.0	1.4	24.5	53.0	
826.6	PC 36:4	PC 18:2/18:2		Area			1.4	Control	4.8	20.5	*	%	16.1	6.5	12.3	12.6	**
		PC 18:1/18:3		Area			1.8	Control	21.5	23.0	*	%	31.7	10.3	10.4	15.4	**
		PC 16:0/20:4 (a)		Area			1.4	GM	20.4	13.3		%	7.8	6.3	29.4	3.3	
		PC 16:0/20:4 (b)		Area			2.9	GM	22.4	8.0	**	%	44.5	76.9	5.5	2.4	**
				Total			1.7	GM	16.8	10.1	**	Ratio b/a	6.0	12.2	27.0	5.6	**
830.6	PC 36:2	PC 18:1/18:1		Area			3.7	Control	1.3	16.7	**	%	70.5	34.5	5.3	12.2	**
		PC 16:0/20:2 (a)		Area			1.4	Control	20.8	19.7		%	12.5	15.6	16.7	6.7	
		PC 18:0/18:2	PC 16:0/20:2 (b)	Area			1.6	GM	15.2	13.2	*	%	17.0	49.9	10.9	8.4	**
				Total			1.8	Control	4.2	12.7	**	Ratio b/a	1.4	3.2	10.9	11.1	**
852.6	PC 38:5	PC 16:0/22:5	PC 18:1/20:4	Area			1.1	GM	15.2	11.6		%	58.5	58.7	4.8	3.4	
		PC 18:0/20:5		Area			1.1	GM	20.8	8.6		%	41.5	41.3	6.8	4.8	
				Total			1.1	GM	16.9	9.6							
854.6	PC 38:4	PC 18:0/20:4 (a)	PC 16:0/22:4	Area			3.6	GM	25.2	25.5	**	%	11.6	17.4	12.3	10.2	*
		PC 18:0/20:4 (b)		Area			2.2	GM	14.8	14.0	**	%	88.4	82.6	1.6	2.1	*
				Total			2.4	GM	15.8	16.0	**	Ratio b/a	7.7	4.8	14.9	12.2	*
872.5	PC 40:9	PC 20:4/20:5		Area			1.7	GM	34.1	27.4		%	16.0	22.6	11.4	24.8	
		PC 18:3/22:6		Area			1.2	GM	27.0	25.4		%	84.0	77.4	2.2	7.2	
				Total			1.3	GM	27.9	22.2							**
874.6	PC 40:8	PC 18:3/22:5		Area			2.9	GM	19.9	10.5	**	%	3.9	10.0	26.4	17.5	**
		PC 20:4/20:4	PC 20:3/20:5	Area			2.5	GM	9.7	6.1	**	%	12.0	27.1	14.7	7.8	**
		PC 18:2/22:6		Area			1.2	Control	14.7	17.6		%	84.1	62.9	2.6	5.5	**
				Total			1.1	GM	12.2	12.6							
876.6	PC 40:7	PC 18:2/22:5		Area			1.8	GM	31.9	9.8	*	%	3.5	14.6	35.0	19.6	**
		PC 20:2/20:5		Area			1.9	GM	3.5	26.4	*	%	3.7	16.6	5.5	17.0	**
		PC 18:1/22:6 (a)		Area			2.3	Control	8.7	11.4	**	%	35.1	36.3	8.7	8.8	
		PC 18:1/22:6 (b)		Area			4.2	Control	11.1	22.7	**	%	57.7	32.5	4.2	10.1	**
				Total			2.4	Control	7.4	12.8	**	Ratio b/a	1.7	0.9	12.3	18.5	**
882.6	PC 40:4	PC 20:3/20:1		Area			N/A	GM	0.0	36.2	**	%	0.0	40.9	0.0	9.4	**
		PC 20:0/20:4		Area			N/A	GM	0.0	37.9	*	%	0.0	59.1	0.0	6.5	**
				Total			N/A	GM	0.0	36.6	**						
898.6	PC 42:10	PC 20:5/22:5		Area			2.4	GM	41.3	31.0	*	%	12.3	14.0	48.4	27.8	
		PC 20:4/22:6 (a)		Area			1.2	GM	33.8	11.5		%	46.4	28.5	11.2	13.4	**
		PC 20:4/22:6 (b)		Area			2.7	GM	23.5	3.0	**	%	41.3	57.5	4.1	2.9	**
				Total			2.0	GM	22.7	3.6	**	Ratio a/b	1.1	0.5	10.4	14.2	**
900.6	PC 42:9	PC 20:4/22:5		Area			6.9	GM	19.9	17.4	**	%	16.9	38.1	51.9	6.2	*
		PC 20:3/22:6		Area			1.9	GM	55.1	7.9		%	83.1	61.9	10.6	3.8	*
				Total			2.6	GM	49.3	11.4	**						

Supplementary Table S15. LC-MS/MS data for gill tissue at the final timepoint. Green and red represent high and low absolute peak areas, respectively. p < 0.01 = **, p= 0.01-0.05 = *. Stars with a red background denote falling below the Benjamini-Hochberg critical value.

MW	Simple ID	ID1	ID2		Control	GM	Fold	Highest	RSD%	RSD%	P		Control	GM	RSD%	RSD%	P
746.5	PE O-38:7	PE P-18:1/20:5		Area			3.1	Control	47.7	18.3		%	**61.8**	**42.5**	9.6	20.0	*
		PE P-16:0/22:6		Area			1.3	Control	28.7	18.5		%	**38.2**	**57.5**	15.5	14.7	*
				Total			2.1	Control	40.7	7.7							
748.5	PC 30:1	PC 14:0/16:1 (a)		Area			4.3	Control	12.1	42.2	**	%	**69.4**	**100.0**	8.5	0.0	**
		PC 14:0/16:1 (b)		Area			N/A	Control	34.8	0.0	*	%	**30.6**	**0.0**	19.3	0.0	**
				Total			9.4	Control	18.7	97.5	**		**2.4**	**0**	29.6	0	*
762.5	PE 38:6	PE 18:1/20:5		Area			5.1	Control	16.8	47.7	**	%	**21.0**	**7.5**	12.4	47.9	**
		PE 16:0/22:6		Area			1.5	Control	24.2	15.1		%	**79.0**	**92.5**	3.3	3.9	**
				Total			1.8	Control	22.0	13.2	*						
764.5	PE 38:5	PE 16:0/22:5	PE 18:1/20:4	Area			1.7	Control	13.8	3.1	**	%	**56.4**	**58.4**	13.4	6.7	
		PE 18:0/20:5		Area			1.9	Control	37.4	14.4		%	**43.6**	**41.6**	17.3	9.4	
				Total			1.7	Control	22.1	5.5	*						
774.5	PE O-40:7	PE P-18:1/22:5		Area			2.9	Control	37.9	21.9	*	%	**30.5**	**15.9**	10.5	34.0	*
		PE P-18:0/22:6		Area			1.2	Control	41.4	19.7		%	**69.5**	**84.1**	4.6	6.4	*
				Total			1.5	Control	40.0	13.9							
776.5	PC 32:1	PC 16:0/16:1 (a)	PC 14:0/18:1	Area			2.7	Control	8.2	16.9	**	%	**70.0**	**44.4**	2.6	5.7	**
		PC 16:0/16:1 (b)		Area			1.0	GM	6.4	8.0		%	**25.5**	**43.4**	4.0	6.1	**
		PC 16:0/16:1 (c)	PC 14:0/18:1	Area			1.6	GM	14.6	9.7	**	%	**4.5**	**12.2**	18.8	8.8	**
				Total			1.7	Control	6.5	11.2	**						
822.5	PC 36:6	PC 16:1/20:5		Area			4.1	Control	28.6	61.0	*	%	**30.6**	**25.9**	7.2	13.4	
		PC 14:0/22:6		Area			3.3	Control	24.9	46.0	*	%	**69.4**	**74.1**	3.2	4.7	
				Total			3.5	Control	25.6	49.8	*						
826.6	PC 36:4	PC 18:2/18:2		Area			1.3	Control	46.6	11.6		%	**6.7**	**3.4**	43.1	17.4	
		PC 18:1/18:3		Area			1.4	GM	20.3	23.3		%	**16.6**	**14.4**	19.2	13.5	
		PC 16:0/20:4 (a)		Area			1.1	GM	9.7	24.3		%	**9.1**	**6.2**	13.1	10.0	*
		PC 16:0/20:4 (b)		Area			1.7	GM	15.5	13.1	**	%	**67.6**	**76.0**	8.1	3.4	
				Total			1.6	GM	10.9	14.1	*	Ratio b/a	**7.5**	**12.3**	12.6	12.3	**
830.6	PC 36:2	PC 18:1/18:1		Area			3.0	Control	21.9	19.8	**	%	**70.5**	**39.7**	4.4	5.8	**
		PC 16:0/20:2 (a)		Area			1.1	GM	6.0	9.8		%	**8.2**	**15.3**	11.8	7.6	**
		PC 18:0/18:2	PC 16:0/20:2 (b)	Area			1.3	GM	8.0	10.6	*	%	**21.3**	**44.9**	10.2	3.3	**
				Total			1.7	Control	17.5	14.0	*	Ratio b/a	**2.6**	**2.9**	5.6	6.3	
836.6	PC O-38:6	PC P-18:0/20:5		Area			2.8	Control	26.3	34.9	*	%	**36.7**	**24.1**	18.2	25.4	
		PC O-16:0/22:6		Area			1.6	Control	1.4	14.4	**	%	**63.3**	**75.9**	10.5	8.1	
				Total			1.9	Control	9.2	17.0	**						
850.6	PC 38:6	PC 18:1/20:5		Area			3.1	Control	25.8	50.2	*	%	**19.4**	**7.7**	11.9	42.4	**
		PC 16:0/22:6		Area			1.1	Control	10.9	5.1		%	**80.6**	**92.3**	2.9	3.5	**
				Total			1.3	Control	13.7	8.1							
854.6	PC 38:4	PC 18:1/20:3		Area			1.9	Control	18.5	7.8	*	%	**14.2**	**4.9**	20.3	17.8	**
		PC 16:0/22:4		Area			1.7	GM	21.4	21.0	*	%	**16.8**	**19.0**	17.3	8.4	
		PC 18:0/20:4		Area			1.7	GM	7.9	12.1	**	%	**69.1**	**76.1**	6.2	1.7	
				Total			1.5	GM	5.7	12.9	*						
876.6	PC 40:7	PC 18:1/22:6 (a)		Area			1.6	Control	21.3	19.7		%	**31.6**	**49.9**	29.4	18.5	
		PC 18:1/22:6 (b)		Area			3.2	Control	23.0	49.1	*	%	**68.4**	**50.1**	13.6	18.5	
				Total			2.4	Control	9.6	32.2	**	Ratio b/a	**2.4**	**1.0**	47.0	36.2	

Supplementary Table S16. LC-MS/MS data for gill tissue at the intermediate timepoint. Green and red represent high and low absolute peak areas, respectively. p < 0.01 = **, p= 0.01-0.05 = *. Stars with a red background denote falling below the Benjamini-Hochberg critical value.

MW	Simple ID	ID1	ID2		Control	GM	Fold	Highest	RSD%	RSD%	P		Control	GM	RSD%	RSD%	P
748.5	PC 30:1	PC 14:0/16:1 (a)		Area			5.1	Control	37.4	19.7	*	%	**35.1**	**34.7**	10.8	19.3	
		PC 14:0/16:1 (b)		Area			4.8	Control	37.5	30.6	*	%	**40.0**	**41.1**	16.5	6.1	
		PC 14:1/16:0		Area			4.9	Control	28.2	36.5	**	%	**24.9**	**24.2**	17.2	27.6	
				Total			4.9	Control	31.3	24.3	*	Ratio a/b	**0.9**	**0.8**	26.1	21.5	
776.5	PC 32:1	PC 16:0/16:1 (a)	PC 14:0/18:1	Area			2.9	Control	2.1	18.7	**	%	**50.8**	**38.5**	2.0	6.8	**
		PC 16:0/16:1 (b)		Area			1.8	Control	5.8	26.1	**	%	**36.9**	**45.2**	3.4	3.1	**
		PC 16:0/16:1 (c)		Area			2.3	GM	24.2	34.2		%	**2.7**	**13.4**	21.7	13.7	**
		PC 16:0/16:1 (d)		Area			7.1	Control	16.1	36.4	**	%	**9.5**	**2.9**	15.0	14.7	**
				Total			2.2	Control	4.0	24.3	**						
790.5	PC 33:1	PC 16:0/17:1 (a)	PC 15:0/18:1	Area			2.6	Control	9.8	22.8	**	%	**71.8**	**67.3**	7.7	10.4	
		PC 16:0/17:1 (b)		Area			2.1	Control	28.8	32.5		%	**28.2**	**32.7**	19.6	21.4	
				total			2.4	Control	11.7	22.7	**	Ratio a/b	**2.6**	**2.1**	25.0	30.6	
802.6	PC 34:2	PC 16:1/18:1		Area			6.3	Control	14.6	5.5	**	%	**15.7**	**3.3**	21.4	11.6	**
		PC 16:0/18:2 (a)		Area			1.4	Control	12.1	15.3	*	%	**79.6**	**76.7**	4.0	3.8	
		PC 16:0/18:2 (b)		Area			3.2	GM	13.6	22.5	**	%	**4.7**	**20.0**	5.6	15.1	**
				Total			1.3	Control	8.5	14.5	*	Ratio a/b	**17.0**	**3.9**	4.0	19.1	**
822.5	PC 36:6	PC 16:1/20:5		Area			10.1	Control	22.1	173.2	**	%	**19.6**	**33.3**	7.9	173.2	
		PC 14:0/22:6		Area			6.2	Control	15.8	99.6	**	%	**80.4**	**66.7**	1.9	86.6	
				total			6.7	Control	16.8	67.8	**						
826.6	PC 36:4	PC 18:2/18:2		Area			1.3	Control	28.1	34.7		%	**12.3**	**6.4**	17.7	23.0	*
		PC 18:1/18:3		Area			1.9	Control	10.5	34.6	*	%	**26.8**	**9.3**	4.7	15.8	**
		PC 16:0/20:4		Area			2.0	GM	15.8	19.9	*	%	**60.9**	**84.3**	3.7	2.5	**
				Total			1.5	GM	14.8	21.5							
828.6	PC 36:3	PC 18:1/18:2		Area			2.6	Control	7.9	27.1	**	%	**79.1**	**50.6**	3.0	6.2	**
		PC 16:0/20:3 (a)		Area			1.1	Control	15.9	35.1		%	**19.9**	**31.1**	11.1	17.2	*
		PC 16:0/20:3 (b)		Area			11.4	GM	19.9	22.4	**	%	**1.0**	**18.3**	18.2	13.3	**
				Total			1.6	Control	8.3	26.7	*	Ratio a/b	**20.7**	**1.7**	8.5	27.9	**
830.6	PC 36:2	PC 18:1/18:1		Area			5.6	Control	10.9	17.1	**	%	**66.3**	**30.9**	6.2	10.5	**
		PC 16:0/20:2 (a)		Area			1.7	Control	11.5	36.6	*	%	**12.0**	**17.6**	15.7	17.9	
		PC 16:0/20:2 (b)	PC 18:0/18:2	Area			1.1	Control	7.5	24.5		%	**21.7**	**51.5**	10.2	9.1	**
				Total			2.6	Control	5.7	22.3	**	Ratio b/a	**1.8**	**3.0**	5.7	23.8	*
850.6	PC 38:6	PC 18:2/20:4		Area			N/A	GM	0.0	13.7	**	%	**0.0**	**7.3**	0.0	6.7	**
		PC 18:1/20:5		Area			4.3	Control	7.1	17.3	**	%	**20.2**	**8.0**	11.6	2.0	**
		PC 16:0/22:6		Area			1.6	Control	11.8	17.8	*	%	**79.8**	**84.7**	2.9	0.7	*
				Total			1.7	Control	9.4	17.3	**						
854.6	PC 38:4	PC 16:0/22:4		Area			3.5	GM	17.0	27.0	*	%	**15.4**	**30.8**	30.5	8.9	**
		PC 18:0/20:4		Area			1.4	GM	23.6	24.4		%	**84.6**	**69.2**	5.6	4.0	**
				total			1.7	GM	17.8	24.2							
876.6	PC 40:7	PC 18:1/22:6 (a)		Area			3.2	Control	13.4	40.7	**	%	**36.4**	**45.2**	5.7	15.0	
		PC 18:1/22:6 (b)		Area			4.8	Control	11.2	18.0	**	%	**63.6**	**54.8**	3.3	12.3	
				total			4.0	Control	11.3	28.5	**	Ratio b/a	**1.8**	**1.2**	9.1	29.2	

Supplementary Table S17. LC-MS/MS data for intestine tissue at the final timepoint. Green and red represent high and low absolute peak areas, respectively. p < 0.01 = **, p= 0.01-0.05 = *. Stars with a red background denote falling below the Benjamini-Hochberg critical value.

MW	Simple ID	ID1	ID2		Control	GM	Fold	Higest	RSD%	RSD%	P		Control	GM	RSD%	RSD%	P
734.5	PE 36:6	PE 16:1/20:5		Area			N/A	Control	38.1	0.0	*	%	39.2	0.0	21.9	0.0	**
		PE 14:0/22:6		Area			N/A	Control	44.5	0.0	*	%	60.8	0.0	14.1	0.0	**
				total			N/A	Control	38.8	0.0	*						
740.5	PE 36:3	PE 18:1/18:2		Area			3.0	Control	40.1	18.4	*	%	71.9	50.9	5.7	11.6	**
		PE 16:0/20:3		Area			1.1	Control	25.5	42.2		%	28.1	49.1	14.7	12.0	**
				total			2.0	Control	35.2	30.1							
742.5	PE 36:2	PE 18:1/18:1		Area			13.7	Control	36.6	56.4	*	%	64.3	18.3	8.7	37.6	**
		PE 18:0/18:2 (a)		Area			7.0	Control	15.6	34.9	**	%	21.5	12.4	20.1	44.5	
		PE 18:0/18:2 (b)		Area			1.3	GM	20.9	17.1		%	14.2	69.3	15.2	2.0	**
				Total			4.0	Control	29.2	18.8	*	Ratio a/b	1.5	0.2	18.2	43.1	**
760.5	PE 38:7	PE 18:2/20:5		Area			1.5	Control	28.5	22.6		%	80.6	94.9	4.1	2.3	**
		PE 16:1/22:6		Area			7.0	Control	47.0	48.5	*	%	19.4	5.1	17.2	42.6	**
				total			1.8	Control	31.9	21.9							
762.5	PE 38:6	PE 18:2/20:4		Area			2.1	GM	35.0	11.1	**	%	2.9	9.9	12.0	11.3	**
		PE 18:1/20:5		Area			5.9	Control	28.6	22.5	**	%	37.3	9.9	6.5	12.6	**
		PE 16:0/22:6		Area			1.2	Control	22.5	16.8		%	59.9	80.3	4.6	2.5	**
				Total			1.6	Control	24.8	15.9							
764.5	PE 38:5	PE 16:0/22:5	PE 18:1/20:4	Area			1.2	Control	20.6	23.8		%	34.1	37.4	3.8	4.1	*
		PE 18:0/20:5		Area			1.4	Control	22.5	18.3		%	65.9	62.6	2.0	2.5	*
				total			1.3	Control	21.8	20.3							
765.5	PG 36:6	PG 16:1/20:5		Area			N/A	Control	26.3	0.0	**	%	49.8	0.0	18.2	0.0	**
		PG 14:0/22:6		Area			N/A	Control	30.1	0.0	**	%	50.2	0.0	18.0	0.0	**
				total			N/A	Control	22.1	0.0	**						
766.5	PE 38:4	PE 16:0/22:4		Area			2.2	GM	23.0	28.8	*	%	11.1	11.2	12.3	17.0	
		PE 18:0/20:4		Area			2.1	GM	9.5	11.9	**	%	88.9	88.8	1.5	2.2	
				total			2.2	GM	11.0	13.8	**						
788.5	PE 40:7	PE 18:2/22:5		Area			1.1	GM	53.2	30.1		%	6.4	18.4	20.2	10.5	**
		PE 20:2/20:5		Area			1.3	Control	18.2	34.0		%	5.5	10.2	22.8	22.9	*
		PE 18:1/22:6		Area			3.1	Control	31.2	18.5	*	%	88.1	71.4	1.3	5.4	**
				Total			2.5	Control	31.8	21.3	*						
790.5	PE 40:6	PE 18:1/22:5		Area			2.6	Control	30.4	32.0	*	%	13.7	5.2	7.3	15.1	**
		PE 18:0/22:6		Area			1.1	GM	22.5	18.3		%	86.3	94.8	1.2	0.8	**
				total			1.0	Control	23.6	19.1							
793.5	PG 38:6	PG 18:1/20:5		Area			3.6	Control	22.1	36.4	**	%	42.4	15.7	10.0	16.2	**
		PG 16:0/22:6		Area			1.1	GM	9.8	18.1		%	57.6	84.3	7.3	3.0	**
				total			1.4	Control	13.6	20.9							
794.6	PE 40:4	PE 18:0/22:4		Area			2.8	GM	34.9	15.3	**	%	65.3	57.6	8.5	5.3	
		PE 20:0/20:4		Area			3.9	GM	37.6	17.8	**	%	34.7	42.4	16.0	7.2	
				total			3.1	GM	34.1	15.0	**						
798.5	PC 34:4	PC 16:1/18:3		Area			2.2	Control	33.8	11.2		%	59.2	54.0	16.6	73.7	
		PC 16:0/18:4		Area			1.0	Control	24.9	89.8		%	40.8	46.0	24.1	86.7	
				total			1.5	Control	25.4	51.4							
802.6	PC 34:2	PC 16:1/18:1		Area			7.7	Control	19.4	58.8	**	%	19.7	3.4	4.9	51.8	**
		PC 16:0/18:2		Area			1.1	Control	14.7	20.9		%	80.3	96.6	1.2	1.8	**
				total			1.4	Control	15.6	21.8							
818.6	PE 42:6	PE 20:1/22:5		Area			1.0	Control	27.0	21.5		%	39.3	24.9	4.4	5.9	**
		PE 20:0/22:6		Area			2.0	GM	19.2	22.1	*	%	60.7	75.1	2.8	2.0	**
				total			1.6	GM	22.3	21.6							
820.6	PE 42:5	PE 20:1/22:4		Area			2.0	GM	32.3	28.3		%	69.1	59.4	6.9	4.7	*
		PE 20:0/22:5		Area			3.1	GM	12.7	19.5	**	%	30.9	40.6	15.5	6.9	*
				total			2.3	GM	26.4	24.7	*						
822.5	PC 36:6	PC 18:3/18:3		Area			4.3	GM	29.7	44.6	*	%	4.5	53.9	11.7	18.1	**
		PC 16:1/20:5		Area			7.2	Control	35.4	65.0	*	%	46.2	17.4	4.6	17.6	**
		PC 14:0/22:6		Area			4.8	Control	34.7	63.4	*	%	49.4	28.7	3.4	24.6	**
				Total			2.8	Control	34.6	50.0	*						
824.5	PC 36:5	PC 18:2/18:3		Area			2.0	GM	30.0	28.4		%	5.8	13.9	24.3	5.2	**
		PC 16:0/20:5		Area			1.3	Control	25.1	26.7		%	94.2	86.1	1.5	0.8	**
				total			1.2	Control	24.8	26.9							
826.6	PC 36:4	PC 18:2/18:2		Area			1.0	Control	21.2	25.3		%	20.4	14.5	1.0	4.7	**
		PC 18:1/18:3		Area			1.5	Control	26.2	37.9		%	42.2	20.4	7.5	16.0	**
		PC 16:0/20:4 (a)		Area			1.0	GM	16.3	15.9		%	16.1	12.2	5.0	9.4	**
		PC 16:0/20:4 (b)		Area			3.3	GM	16.4	20.3	**	%	21.3	52.8	14.2	4.5	**
				Total			1.3	GM	20.7	24.1		Ratio b/a	1.3	4.3	13.1	4.9	**
828.6	PC 36:3	PC 18:1/18:2		Area			2.9	Control	15.0	36.8	**	%	61.3	28.1	4.5	9.7	**
		PC 16:0/20:3 (a)		Area			1.2	GM	20.2	19.8		%	34.4	58.3	6.4	9.8	**
		PC 16:0/20:3 (b)		Area			2.5	GM	19.3	49.7		%	4.3	13.7	17.5	31.0	*
				Total			1.4	Control	15.9	28.5		Ratio a/b	8.2	4.7	13.8	47.3	

830.6	PC 36:2	PC 18:1/18:1		Area			6.1	Control	17.3	40.7	**	%	**71.6**	**28.6**	1.7	18.3	**
		PC 16:0/20:2		Area			1.4	Control	11.4	5.8	*	%	**9.6**	**17.5**	18.9	28.4	
		PC 18:0/18:2		Area			1.1	GM	22.7	23.8		%	**18.8**	**53.9**	5.7	1.3	**
				Total			2.5	Control	17.0	24.0	**						
846.5	PC 38:8	PC 18:3/20:5		Area			1.5	GM	38.8	43.3		%	**89.4**	**94.0**	4.0	1.2	
		PC 16:2/22:6		Area			1.1	Control	28.3	54.5		%	**10.6**	**6.0**	34.3	18.3	
				total			1.5	GM	36.7	43.9							
850.6	PC 38:6	PC 18:3/20:3		Area			2.0	GM	31.4	14.5	*	%	**2.1**	**5.1**	16.5	10.3	**
		PC 18:2/20:4		Area			4.2	GM	54.2	48.7		%	**1.2**	**6.3**	32.0	41.0	*
		PC 18:1/20:5		Area			3.4	Control	29.6	39.7	*	%	**34.4**	**12.1**	7.1	18.4	**
		PC 16:0/22:6		Area			1.0	GM	20.8	20.3		%	**62.3**	**76.5**	5.0	4.9	**
				Total			1.2	Control	24.2	22.9							
854.6	PC 38:4	PC 18:1/20:3		Area			1.1	GM	12.2	33.0		%	**42.5**	**24.3**	8.4	10.0	**
		PC 16:0/22:4		Area			2.1	GM	15.6	15.4	**	%	**17.1**	**19.8**	3.5	11.8	
		PC 18:0/20:4		Area			2.6	GM	28.7	25.8	*	%	**40.3**	**55.9**	9.8	2.9	**
				Total			1.9	GM	18.9	25.3	*						
872.5	PC 40:9	PC 20:4/20:5		Area			1.2	Control	33.2	22.2		%	**32.4**	**16.6**	17.9	8.5	*
		PC 18:3/22:6		Area			2.1	GM	21.5	25.7	*	%	**67.6**	**83.4**	8.6	1.7	*
				total			1.7	GM	23.6	24.8							
874.6	PC 40:8	PC 18:3/22:5		Area			2.0	GM	19.8	23.6	*	%	**14.2**	**20.4**	5.8	7.2	**
		PC 20:3/20:5		Area			2.1	GM	8.1	39.7		%	**16.3**	**22.2**	20.2	15.0	
		PC 18:2/22:6 (a)		Area			1.1	GM	28.0	26.9		%	**68.5**	**52.5**	5.4	5.6	**
		PC 18:2/22:6 (b)		Area			7.8	GM	54.2	31.1	**	%	**0.9**	**4.9**	42.7	11.6	**
				Total			1.4	GM	22.5	28.7		Ratio a/b	**90.5**	**10.8**	53.7	16.0	*
876.6	PC 40:7	PC 18:2/22:5		Area			1.3	GM	32.0	23.8		%	**12.1**	**28.4**	7.6	20.1	**
		PC 20:2/20:5		Area			1.5	GM	24.0	30.7		%	**5.3**	**13.6**	8.9	11.5	**
		PC 18:1/22:6		Area			2.5	Control	29.4	31.8	*	%	**82.6**	**57.9**	1.5	7.7	**
				Total			1.7	Control	29.3	25.8							
878.6	PC 40:6	PC 18:1/22:5		Area			3.1	Control	21.1	35.3	**	%	**29.6**	**8.7**	2.2	11.9	**
		PC 20:1/20:5		Area			1.4	GM	28.1	33.1		%	**16.8**	**21.2**	5.7	10.6	*
		PC 18:0/22:6		Area			1.4	GM	21.2	23.0		%	**53.7**	**70.1**	1.8	4.2	**
				Total			1.1	GM	22.3	26.0							
880.6	PC 40:5	PC 18:0/22:5		Area			1.6	GM	27.0	17.7		%	**80.4**	**76.6**	4.9	6.3	
		PC 20:0/20:5	PC 20:1/20:4	Area			2.0	GM	45.3	39.8		%	**19.6**	**23.4**	19.9	20.8	
				total			1.7	GM	30.0	22.8							
882.6	PC 40:4	PC 18:0/22:4	PC 20:1/20:3	Area			2.6	GM	39.8	12.3	**	%	**67.3**	**56.4**	4.8	9.5	*
		PC 20:0/20:4		Area			4.3	GM	28.8	24.7	**	%	**32.7**	**43.6**	9.8	12.3	*
				total			3.2	GM	35.9	15.6	**						
898.6	PC 42:10	PC 20:5/22:5		Area			1.1	Control	41.7	33.6		%	**45.3**	**30.2**	21.5	9.7	
		PC 20:4/22:6 (a)		Area			1.3	GM	10.0	7.6	*	%	**42.3**	**39.0**	27.1	23.1	
		PC 20:4/22:6 (b)		Area			3.5	GM	34.0	42.4	*	%	**12.4**	**30.8**	14.4	19.8	**
				Total			1.4	GM	24.7	25.5		Ratio a/b	**3.5**	**1.3**	43.1	43.6	
900.6	PC 42:9	PC 20:4/22:5		Area			1.7	GM	13.0	20.2	*	%	**22.8**	**16.5**	29.7	18.7	
		PC 20:5/22:4		Area			4.0	GM	55.0	39.0	*	%	**11.2**	**19.9**	32.4	25.4	
		PC 20:3/22:6		Area			2.2	GM	28.4	8.2	**	%	**66.1**	**63.6**	7.4	7.9	
				Total			2.3	GM	22.9	14.0	**						
906.6	PC 42:6	PC 20:2/22:4		Area			1.0	GM	17.0	18.9		%	**20.7**	**11.3**	20.2	27.9	*
		PC 20:1/22:5		Area			1.2	GM	42.3	23.2		%	**53.1**	**33.5**	7.2	3.6	**
		PC 20:0/22:6		Area			3.9	GM	38.9	26.0	**	%	**26.3**	**55.2**	2.9	4.6	**
				Total			1.9	GM	36.1	22.0							

Supplementary Table S18. LC-MS/MS data for intestine tissue at the intermediate timepoint. Green and red represent high and low absolute peak areas, respectively. p < 0.01 = **, p= 0.01-0.05 = *. Stars with a red background denote falling below the Benjamini-Hochberg critical value.

MW	Simple ID	ID1	ID2		Control	GM	Fold	Highest	RSD%	RSD%	P		Control	GM	RSD%	RSD%	P
740.5	PE 36:3	PE 18:1/18:2		Area			2.6	Control	18.2	48.8	*	%	77.4	52.3	2.8	8.6	**
		PE 16:0/20:3		Area			1.2	GM	8.9	37.8		%	22.6	47.7	9.5	9.4	**
				total			1.8	Control	15.8	43.6							
742.5	PE 36:2	PE 18:1/18:1		Area			5.3	Control	19.2	59.5	**	%	71.2	27.8	8.1	29.3	**
		PE 18:0/18:2		Area			1.2	GM	9.7	51.8		%	28.8	72.2	20.0	11.3	**
				total			2.2	Control	12.3	51.5	*						
762.5	PE 38:6	PE 18:2/20:4		Area			1.1	GM	124.3	36.4		%	7.9	10.3	130.8	11.3	
		PE 18:1/20:5		Area			3.0	Control	87.9	52.7		%	17.8	7.8	86.8	7.5	
		PE 16:0/22:6		Area			1.2	Control	16.3	45.8		%	74.3	81.9	7.1	0.9	
				Total			1.3	Control	17.9	45.3							
766.5	PE 38:4	PE 18:1/20:3		Area			2.0	Control	11.8	60.9		%	16.9	2.3	6.2	23.2	**
		PE 16:0/22:4		Area			3.9	GM	30.6	50.9		%	8.1	8.7	22.6	10.7	
		PE 18:0/20:4		Area			4.2	GM	11.4	39.8	*	%	75.0	89.0	1.1	1.6	**
				Total			3.5	GM	12.1	41.2	*						
768.5	PE 38:3	PE 18:2/20:1		Area			1.8	Control	10.6	65.0		%	61.5	39.8	7.5	17.4	*
		PE 18:0/20:3		Area			1.2	GM	19.1	45.7		%	38.5	60.2	11.9	11.6	*
				total			1.2	Control	11.0	51.6							
774.5	PC 32:2	PC 14:0/18:2		Area			1.1	GM	16.3	53.7		%	43.2	78.1	6.6	5.9	**
		PC 16:1/16:1		Area			3.9	Control	26.8	76.0	*	%	56.8	21.9	5.0	21.2	**
				total			1.6	Control	22.3	58.7							
788.5	PE 40:7	PE 18:3/22:4		Area			2.5	GM	21.5	43.3		%	1.8	10.4	17.9	8.8	**
		PE 20:2/20:5		Area			1.3	Control	39.1	52.6		%	3.3	5.8	25.7	3.2	**
		PE 18:1/22:6 (a)		Area			2.8	Control	13.8	53.7	**	%	93.4	76.5	0.9	0.6	**
		PE 18:1/22:6 (b)		Area			2.2	GM	19.9	62.1		%	1.5	7.3	28.6	8.5	**
				Total			2.3	Control	14.2	53.2	*	Ratio a/b	65.5	10.5	33.8	7.8	*
792.6	PE 40:5	PE 18:0/22:5 (a)	PE 20:1/20:4	Area			2.3	GM	15.2	48.1		%	66.1	76.1	2.0	5.5	*
		PE 20:0/20:5		Area			1.9	GM	19.2	63.1		%	11.9	10.5	6.7	13.1	
		PE 18:0/22:5 (b)		Area			1.3	GM	4.3	68.8		%	22.0	13.4	8.5	20.8	*
				Total			2.0	GM	13.2	52.7		Ratio a/b	3.0	5.9	10.8	28.1	*
794.6	PE 40:4	PE 18:1/22:3		Area			6.5	GM	7.9	44.1	*	%	21.5	23.4	5.0	15.7	
		PE 18:0/22:4		Area			4.8	GM	15.1	36.2	*	%	66.4	55.4	4.4	4.8	**
		PE 20:0/20:4		Area			10.4	GM	7.7	38.7	*	%	12.1	21.2	17.5	5.5	**
				Total			5.9	GM	10.7	38.0	*						
802.6	PC 34:2	PC 16:0/18:2 (a)		Area			1.5	Control	5.2	47.4		%	97.9	87.0	0.4	2.2	**
		PC 16:0/18:2 (b)	PC 14:0/20:2	Area			4.4	GM	25.7	40.3	*	%	2.1	13.0	19.8	14.7	**
				total			1.4	Control	5.6	46.1		Ratio a/b	47.4	6.8	19.0	16.4	**
810.5	PE 42:10	PE 20:4/22:6 (a)		Area			1.7	GM	21.2	44.6		%	82.4	64.2	3.0	6.6	**
		PE 20:4/22:6 (b)		Area			4.7	GM	5.7	60.9		%	17.6	35.8	13.9	11.8	**
				total			2.2	GM	18.1	50.6		Ratio a/b	4.8	1.8	18.2	18.9	**
818.6	PE 42:6	PE 20:1/22:5		Area			1.1	Control	18.7	63.6		%	26.4	10.4	14.1	10.8	**
		PE 20:0/22:6		Area			2.8	GM	8.7	63.4		%	73.6	89.6	5.1	1.3	**
				total			2.3	GM	9.1	63.4							
820.6	PE 42:5	PE 20:1/22:4		Area			2.0	GM	34.0	56.0		%	84.3	74.1	10.4	2.1	
		PE 20:0/22:5		Area			4.3	GM	29.5	62.5		%	15.7	25.9	55.8	5.9	
				total			2.3	GM	25.4	57.7							
822.5	PC 36:6	PC 16:1/20:5		Area			2.4	Control	39.9	61.7		%	26.0	26.8	19.5	8.0	
		PC 14:0/22:6		Area			2.5	Control	14.2	55.8	*	%	74.0	73.2	6.9	2.9	
				total			2.4	Control	21.1	57.4	*						
824.5	PC 36:5	PC 18:2/18:3		Area			1.1	GM	18.4	78.0		%	6.6	5.4	9.6	33.3	
		PC 16:0/20:5		Area			1.2	GM	16.3	45.3		%	93.4	94.6	0.7	1.9	
				total			1.2	GM	16.3	46.9							
826.6	PC 36:4	PC 18:2/18:2		Area			1.5	Control	7.1	62.7		%	32.5	13.4	11.2	15.3	**
		PC 18:1/18:3		Area			2.2	Control	30.5	62.0		%	36.5	10.5	16.5	17.7	**
		PC 16:0/20:4 (a)		Area			1.3	GM	6.9	43.6		%	7.8	6.3	9.8	16.2	
		PC 16:0/20:4 (b)		Area			4.7	GM	10.6	44.9	*	%	23.2	69.9	7.2	4.2	**
				Total			1.6	GM	16.2	48.5		Ratio b/a	3.0	11.3	4.8	11.8	**

828.6	PC 36:3	PC 18:1/18:2		Area			3.2	Control	12.7	47.9	**	%	**70.3**	**36.2**	6.6	2.1	**
		PC 16:0/20:3 (a)		Area			1.2	GM	17.2	44.2		%	**22.8**	**47.0**	23.3	4.2	**
		PC 16:0/20:3 (b)		Area			1.5	GM	15.6	53.7		%	**6.9**	**16.7**	10.7	7.9	**
				Total			1.7	Control	6.9	47.0		Ratio a/b	**3.4**	**2.8**	33.8	11.9	
830.6	PC 36:2	PC 18:1/18:1		Area			6.9	Control	21.3	37.8	**	%	**59.5**	**16.6**	10.7	13.1	**
		PC 16:0/20:2		Area			1.1	Control	4.7	53.8		%	**8.5**	**13.8**	14.2	12.0	*
		PC 18:0/18:2		Area			1.2	GM	9.1	42.3		%	**32.0**	**69.6**	16.2	1.9	**
				Total			1.9	Control	12.4	42.7	*						
850.6	PC 38:6	PC 18:2/20:4		Area			9.0	GM	33.4	54.5	*	%	**0.8**	**8.0**	30.3	2.4	**
		PC 18:1/20:5		Area			1.6	Control	19.1	55.0		%	**16.0**	**10.8**	16.2	8.8	*
		PC 16:0/22:6		Area			1.1	Control	7.3	51.4		%	**83.1**	**81.2**	3.4	1.2	
				Total			1.1	Control	7.5	52.0							
852.6	PC 38:5	PC 16:0/22:5 (a)	PC 18:1/20:4	Area			1.3	GM	10.0	40.3		%	**43.2**	**35.2**	0.7	9.3	*
		PC 18:0/20:5		Area			2.0	GM	12.1	43.7		%	**47.0**	**58.8**	1.6	6.6	**
		PC 16:0/22:5 (b)		Area			1.0	Control	8.8	46.9		%	**9.8**	**6.0**	6.0	11.9	**
				Total			1.6	GM	10.7	42.2		Ratio a/b	**4.4**	**5.9**	5.7	6.4	**
854.6	PC 38:4	PC 18:2/20:2		Area			1.9	Control	11.3	53.7		%	**26.9**	**6.9**	3.7	17.5	**
		PC 18:3/20:1	PC 18:1/20:3	Area			1.1	Control	5.6	49.4		%	**30.2**	**13.5**	11.4	13.7	**
		PC 18:0/20:4		Area			3.8	GM	17.3	42.8	*	%	**42.9**	**79.6**	7.1	1.6	**
				Total			2.0	GM	10.1	44.2							
856.5	PC 38:3	PC 18:2/20:1	PC 18:1/20:2	Area			2.0	Control	13.7	46.9	*	%	**78.0**	**55.8**	2.0	5.7	**
		PC 18:0/20:3		Area			1.4	GM	13.1	59.1		%	**22.0**	**44.2**	7.2	7.2	**
				total			1.4	Control	13.1	52.4							
872.5	PC 40:9	PC 20:4/20:5		Area			2.1	GM	30.6	58.1		%	**15.8**	**23.3**	21.0	6.3	*
		PC 18:3/22:6		Area			1.4	GM	19.4	66.4		%	**84.2**	**76.7**	3.9	1.9	*
				total			1.5	GM	19.3	64.5							
874.6	PC 40:8	PC 18:3/22:5		Area			2.1	GM	34.8	64.1		%	**4.8**	**7.5**	21.7	1.6	*
		PC 20:4/20:4	C20:3/20:5	Area			3.0	GM	43.3	61.9		%	**10.1**	**22.9**	29.8	1.4	**
		PC 18:2/22:6		Area			1.1	GM	10.3	63.1		%	**85.1**	**69.6**	4.7	0.5	**
				Total			1.4	GM	14.4	62.9							
876.6	PC 40:7	PC 18:2/22:5		Area			1.8	GM	9.0	53.1		%	**6.1**	**18.9**	16.3	5.6	**
		PC 20:2/20:5		Area			1.8	GM	39.2	42.5		%	**6.9**	**23.4**	30.1	12.4	**
		PC 18:1/22:6		Area			2.6	Control	9.3	62.1	*	%	**87.0**	**57.7**	2.2	6.4	**
				Total			1.7	Control	10.2	55.9							
880.6	PC 40:5	PC 18:0/22:5	C16:0/24:5	Area			2.2	GM	15.9	47.7		%	**80.2**	**73.0**	1.8	1.1	**
		PC 20:0/20:5		Area			3.4	GM	15.1	48.2		%	**19.8**	**27.0**	7.2	3.0	**
				total			2.5	GM	15.4	47.8							
882.6	PC 40:4	PC 20:1/20:3 (a)		Area			1.7	Control	12.3	60.6		%	**71.8**	**20.6**	1.5	12.9	**
		PC 20:1/20:3 (b)	PC 18:0/22:4	Area			3.3	GM	13.8	40.5	*	%	**20.3**	**35.3**	5.4	11.8	**
		PC 20:0/20:4		Area			11.6	GM	7.5	58.7		%	**7.8**	**44.1**	4.4	8.2	**
				Total			2.0	GM	12.0	52.6		Ratio a/b	**3.5**	**0.6**	6.8	22.5	**
898.6	PC 42:10	PC 20:5/22:5		Area			2.5	GM	38.5	40.1		%	**17.8**	**17.9**	7.1	9.4	
		PC 20:4/22:6 (a)		Area			1.6	GM	29.6	56.9		%	**56.7**	**34.0**	4.0	12.1	**
		PC 20:4/22:6 (b)		Area			4.7	GM	31.4	38.3	*	%	**25.5**	**48.1**	7.0	6.0	**
				Total			2.5	GM	31.4	45.1		Ratio a/b	**2.2**	**0.7**	10.3	18.2	**
900.6	PC 42:9	PC 20:4/22:5		Area			4.7	GM	15.7	41.9	*	%	**11.2**	**33.8**	35.9	24.7	*
		PC 20:3/22:6		Area			1.1	GM	38.6	53.2		%	**88.8**	**66.2**	4.6	12.6	*
				total			1.5	GM	33.9	48.0							
906.6	PC 42:6	PC 20:0/22:6 (a)		Area			1.1	Control	2.2	45.9		%	**38.8**	**26.8**	3.6	23.7	*
		PC 20:1/22:5		Area			1.1	Control	21.1	63.8		%	**24.7**	**15.4**	17.0	7.3	*
		PC 20:0/22:6 (b)		Area			2.2	GM	10.4	60.5		%	**36.5**	**57.8**	10.6	10.9	**
				Total			1.4	GM	5.8	56.4		Ratio b/a	**0.9**	**2.3**	10.9	30.0	*

Supplementary Table S19. LC-MS/MS data for liver tissue at the final timepoint. Green and red represent high and low absolute peak areas, respectively. p < 0.01 = **, p= 0.01-0.05 = *. Stars with a red background denote falling below the Benjamini-Hochberg critical value.

MW	Simple ID	ID1	ID2		Control	GM	Fold	Highest	RSD%	RSD%	P		Control	GM	RSD%	RSD%	P
742.5	PE 36:2	PE 18:1/18:1		Area			8.7	Control	10.7	37.1	**	%	76.7	29.2	2.3	23.7	**
		PE 18:0/18:2		Area			1.1	Control	1.7	25.0		%	23.3	70.8	7.6	9.8	**
				total			3.3	Control	8.4	24.7	**						
762.5	PE 38:6	PE 18:2/20:4		Area			3.8	GM	36.7	4.2	**	%	4.4	23.6	5.4	19.6	**
		PE 18:1/20:5	PE 18:2/20:4	Area			2.7	Control	32.8	44.4	*	%	40.4	19.6	8.4	24.4	**
		PE 16:0/22:6		Area			1.3	Control	32.9	22.4		%	55.2	56.8	5.8	5.4	
				Total			1.4	Control	32.5	21.7							
764.5	PE 38:5	PE 18:1/20:4	PE 16:0/22:5	Area			1.4	GM	24.5	22.4		%	26.5	40.1	1.8	10.9	**
		PE 18:0/20:5		Area			1.3	Control	24.0	5.8		%	73.5	59.9	0.7	7.3	**
				Total			1.1	Control	24.1	11.7							
766.5	PE 38:4	PE 18:1/20:3		Area			2.4	Control	43.6	33.0		%	27.1	4.0	27.2	37.7	**
		PE 18:0/20:4		Area			4.1	GM	14.2	9.6	**	%	67.2	90.7	9.3	1.9	**
		PE 18:3/22:5		Area			3.0	GM	18.7	30.7	*	%	5.6	5.3	21.8	25.1	
				Total			3.0	GM	21.8	8.9	**						
786.5	PE 40:8	PE 20:3/20:5		Area			1.9	GM	43.0	22.1		%	4.8	7.6	42.5	12.1	
		PE 18:2/22:6		Area			1.1	GM	45.9	23.5		%	95.2	92.4	2.1	1.0	
				Total			1.2	GM	45.2	23.2							
788.5	PE 40:7	PE 18:2/22:5		Area			1.1	GM	31.7	21.9		%	5.3	14.1	10.3	25.5	*
		PE 20:2/20:5		Area			1.1	GM	29.5	14.0		%	4.9	13.7	14.7	30.5	*
		PE 18:1/22:6		Area			2.9	Control	40.6	41.3		%	89.9	72.2	1.4	8.8	**
				Total			2.4	Control	39.4	32.7							
790.5	PE 40:6	PE 18:1/22:5 (a)		Area			2.6	Control	30.0	31.2	*	%	11.0	4.3	10.6	3.4	**
		PE 20:1/20:5		Area			1.4	Control	31.6	24.4		%	12.7	9.6	12.7	20.2	
		PE 18:1/22:5 (b)		Area			5.2	Control	29.6	60.2	*	%	5.4	1.1	7.1	53.5	**
		PE 18:0/22:6		Area			1.2	GM	26.3	29.2		%	70.9	85.0	3.4	2.9	**
				Total			1.0	Control	27.0	28.2		Ratio a/b	2.1	5.4	18.0	74.6	
792.6	PE 40:5	PE 18:0/22:5 (a)		Area			1.8	GM	22.1	19.5	*	%	75.7	82.3	6.2	3.7	
		PE 20:0/20:5		Area			2.2	GM	35.5	20.1	*	%	9.9	13.3	40.4	26.8	
		PE 18:0/22:5 (b)		Area			2.0	Control	36.3	33.4		%	14.4	4.4	15.1	18.6	**
				Total			1.6	GM	21.9	17.4	*	Ratio a/b	5.4	18.9	20.3	18.5	**
793.5	PG 38:6	PG 18:1/20:5		Area			8.3	Control	19.7	3.0	**	%	28.9	15.2	7.3	31.6	*
		PG 16:0/22:6		Area			3.4	Control	9.3	30.9	**	%	71.1	84.8	3.0	5.7	*
				Total			4.1	Control	12.3	26.4	**						
794.6	PE 40:4	PE 20:1/20:3		Area			N/A	GM	0.0	54.2	*	%	0.0	19.8	0.0	35.0	**
		PE 18:0/22:4		Area			N/A	GM	0.0	13.2	**	%	0.0	37.7	0.0	10.6	**
		PE 20:0/20:4		Area			N/A	GM	0.0	8.2	**	%	0.0	42.5	0.0	12.0	**
				Total			N/A	GM	0.0	17.9	**						
818.6	PE 42:6	PE 20:1/22:5		Area			1.2	Control	27.4	45.9		%	14.9	6.3	17.2	34.7	*
		PE 20:0/22:6		Area			2.1	GM	7.3	11.6	**	%	85.1	93.7	3.0	2.3	*
				Total			1.9	GM	10.3	13.9	**						
822.5	PC 36:6	PC 18:3/18:3		Area			6.7	GM	56.5	33.3	*	%	3.0	41.4	53.8	29.2	**
		PC 16:1/20:5	PC 18:2/18:4	Area			3.4	Control	54.9	26.8		%	25.1	16.0	9.0	12.8	**
		PC 14:0/22:6		Area			3.6	Control	51.4	40.4		%	71.9	42.6	0.9	23.9	**
				Total			2.1	Control	51.9	23.0							
826.6	PC 36:4	PC 18:2/18:2		Area			1.0	GM	20.3	5.8		%	25.1	16.8	5.8	8.7	**
		PC 18:1/18:3		Area			1.0	Control	19.7	22.1		%	38.4	23.7	13.8	9.0	*
		PC 16:0/20:4 (a)		Area			1.0	GM	20.8	27.7		%	15.8	10.2	15.5	22.8	*
		PC 16:0/20:4 (b)		Area			3.7	GM	31.7	13.1	**	%	20.7	49.3	21.6	5.6	**
				Total			1.6	GM	18.9	13.9	*	Ratio b/a	1.3	5.1	12.6	25.3	**
828.6	PC 36:3	PC 18:1/18:2		Area			2.0	Control	12.3	11.1	**	%	65.3	36.8	7.2	14.5	**
		PC 16:0/20:3	PC 18:0/18:3	Area			1.7	GM	29.0	29.6		%	34.7	63.2	13.6	8.4	**
				Total			1.1	Control	16.7	22.6							
830.6	PC 36:2	PC 18:1/18:1		Area			3.9	Control	10.8	27.6	**	%	59.0	26.4	11.4	9.7	**
		PC 16:0/20:2 (a)		Area			1.2	Control	28.2	12.3		%	13.5	19.8	27.9	12.6	
		PC 18:0/18:2	PC 16:0/20:2 (b)	Area			1.1	GM	13.4	21.1		%	27.5	53.8	11.7	0.8	**
				Total			1.8	Control	2.4	20.6	**	Ratio b/a	2.1	2.8	21.9	11.9	
848.5	PC 38:7	PC 18:3/20:4		Area			5.8	GM	23.3	44.8	*	%	1.7	6.5	18.8	46.0	*
		PC 18:2/20:5		Area			1.7	GM	50.4	19.1		%	64.0	77.5	10.6	5.0	*
		PC 16:1/22:6 (a)		Area			2.1	Control	38.5	11.3		%	24.1	8.0	12.2	12.9	**
		PC 16:1/22:6 (b)		Area			1.2	GM	40.2	36.0		%	10.2	8.1	36.4	26.1	
				Total			1.4	GM	45.0	17.5		Ratio a/b	2.5	1.1	23.7	42.9	*
854.6	PC 38:4	PC 18:2/20:2		Area			1.2	Control	48.2	39.2		%	25.5	10.6	21.2	30.8	*
		PC 18:3/20:1	PC 18:1/20:3	Area			1.4	GM	23.3	48.6		%	27.5	17.9	8.2	27.8	*
		PC 16:0/22:4	PC 18:0/20:4 (a)	Area			2.0	GM	31.4	2.7	**	%	17.7	17.8	7.9	21.2	
		PC 18:0/20:4 (b)		Area			3.9	GM	17.3	19.9	**	%	29.4	53.7	13.5	7.1	**
				Total			2.1	GM	29.4	21.6	*	Ratio b/a	1.7	3.1	19.8	19.4	*

872.5	PC 40:9	PC 20:4/20:5		Area			2.2	GM	76.4	24.2		%	**27.5**	**20.5**	48.5	7.5	
		PC 18:3/22:6		Area			3.6	GM	6.9	16.0	**	%	**72.5**	**79.5**	18.4	1.9	
				Total			3.2	GM	27.2	17.7	**						
874.6	PC 40:8	PC 18:3/22:5		Area			4.6	GM	59.2	21.0	**	%	**7.1**	**16.0**	20.3	14.3	**
		PC 20:3/20:5	PC 20:4/20:4	Area			4.6	GM	42.8	22.9	**	%	**12.9**	**28.2**	24.6	12.3	**
		PC 18:2/22:6		Area			1.5	GM	36.8	11.5		%	**80.0**	**55.9**	4.2	3.4	**
				Total			2.1	GM	38.5	14.2	*						
878.6	PC 40:6	PC 20:1/20:5		Area			1.2	GM	53.1	25.7		%	**22.0**	**24.4**	17.4	22.0	
		PC 18:0/22:6		Area			1.1	GM	35.8	35.4		%	**78.0**	**75.6**	4.9	7.1	
				Total			1.1	GM	39.7	31.9							
880.6	PC 40:5	PC 18:0/22:5 (a)		Area			1.5	GM	39.8	30.9		%	**80.1**	**76.5**	4.9	4.7	
		PC 20:0/20:5		Area			3.3	GM	68.3	24.4	*	%	**9.0**	**20.6**	32.6	15.2	**
		PC 18:0/22:5 (b)		Area			2.3	Control	39.2	37.2		%	**11.0**	**2.9**	17.2	19.6	**
				Total			1.6	GM	42.1	29.0		Ratio a/b	**7.5**	**26.9**	19.4	25.8	**
881.5	PI 38:6	PI 18:1/20:5		Area			4.8	Control	28.5	25.9	**	%	**58.4**	**41.0**	12.6	32.5	
		PI 16:0/22:6		Area			2.1	Control	2.7	41.7	**	%	**41.6**	**59.0**	17.7	22.5	
				Total			3.2	Control	17.7	27.2	**						
882.6	PC 40:4	PC 20:1/20:3		Area			3.0	GM	29.8	26.6	*	%	**46.1**	**32.2**	26.8	12.6	
		PC 18:0/22:4		Area			3.7	GM	36.7	22.4	**	%	**40.9**	**35.2**	30.1	5.2	
		PC 20:0/20:4		Area			10.1	GM	57.7	13.1	**	%	**13.0**	**32.7**	35.0	9.6	**
				Total			4.3	GM	24.2	19.7	**						
883.5	PI 38:5	PI 18:1/20:4	PI 18:1/20:4	Area			2.7	Control	19.3	18.6	**	%	**52.5**	**73.6**	1.9	1.7	**
		PI 18:0/20:5	PI 18:0/20:5	Area			6.8	Control	19.4	11.7	**	%	**47.5**	**26.4**	2.1	4.7	**
				Total			3.8	Control	19.3	16.8	**						
898.6	PC 42:10	PC 20:5/22:5		Area			2.7	GM	22.6	28.1	*	%	**41.3**	**35.9**	15.2	6.8	
		PC 20:4/22:6 (a)		Area			3.0	GM	52.4	20.9	*	%	**40.3**	**41.6**	14.0	1.8	
		PC 20:4/22:6 (b)		Area			3.6	GM	43.9	16.9	**	%	**18.4**	**22.5**	4.7	7.4	*
				Total			3.0	GM	38.9	22.4	*	Ratio a/b	**2.2**	**1.9**	10.9	5.7	
900.6	PC 42:9	PC 20:4/22:5		Area			7.4	GM	44.9	6.0	**	%	**10.5**	**19.2**	40.1	12.4	*
		PC 20:3/22:6		Area			3.5	GM	37.3	11.6	**	%	**89.5**	**80.8**	4.7	2.9	*
				Total			3.9	GM	34.4	8.8	**						
904.6	PC 42:7	PC 20:2/22:5		Area			1.4	GM	49.5	28.9		%	**12.0**	**12.6**	4.5	5.1	
		PC 20:1/22:6		Area			1.4	GM	51.4	33.3		%	**88.0**	**87.4**	0.6	0.7	
				Total			1.4	GM	51.1	32.7							
906.6	PC 42:6	PC 20:0/22:6 (a)	PC 20:2/22:4	Area			1.3	GM	35.0	37.7		%	**21.0**	**12.8**	19.6	16.7	*
		PC 20:1/22:5		Area			1.4	GM	59.3	22.7		%	**48.1**	**37.2**	15.1	5.2	
		PC 20:0/22:6 (b)		Area			3.3	GM	33.2	24.6	*	%	**30.9**	**50.0**	13.3	6.7	**
				Total			2.0	GM	46.3	25.0		Ratio b/a	**1.5**	**4.0**	16.5	24.9	*
926.6	PC 44:10	PC 22:5/22:5		Area			2.7	GM	42.7	6.8	**	%	**51.5**	**44.6**	12.0	16.0	
		PC 20:5/24:5		Area			2.2	GM	52.1	49.4		%	**26.4**	**19.0**	30.1	37.9	
		PC 22:4/22:6		Area			5.7	GM	9.8	17.1	**	%	**22.1**	**36.4**	34.6	1.9	*
				Total			3.2	GM	35.4	16.6	**						

Supplementary Table S20. LC-MS/MS data for liver tissue at the intermediate timepoint. Green and red represent high and low absolute peak areas, respectively. p < 0.01 = **, p= 0.01-0.05 = *. Stars with a red background denote falling below the Benamini-Hochberg critical value.

MW	Simple ID	ID1	ID2		Control	GM	Fold	Highest	RSD%	RSD%	P		Control	GM	RSD%	RSD%	P
762.5	PE 38:6	PE 18:2/20:4		Area			7.7	GM	30.8	22.0	**	%	2.5	31.1	30.3	8.2	**
		PE 18:1/20:5		Area			5.6	Control	7.0	24.5	**	%	42.7	12.6	7.8	9.3	**
		PE 16:0/22:6		Area			1.6	Control	8.0	33.0		%	54.8	56.3	4.7	5.9	
				Total			1.6	Control	4.6	28.4	*						
764.5	PE 38:5	PE 16:0/22:5	PE 18:1/20:4	Area			1.7	GM	7.6	22.4	*	%	25.0	37.4	8.4	2.8	**
		PE 18:0/20:5		Area			1.1	Control	10.4	24.8		%	75.0	62.6	2.8	1.7	**
				total			1.1	GM	8.4	23.9							
784.5	PE 40:9	PE 20:4/20:5		Area			1.0	Control	20.4	28.4		%	6.5	5.6	22.0	3.7	
		PE 18:3/22:6		Area			1.1	GM	3.3	28.8		%	93.5	94.4	1.5	0.2	
				total			1.1	GM	2.0	28.8							
788.5	PE 40:7	PE 18:2/22:5		Area			1.5	GM	17.6	20.3		%	3.3	15.5	14.4	12.9	**
		PE 20:2/20:5		Area			1.3	GM	5.6	35.1		%	5.3	20.1	3.3	5.7	**
		PE 18:1/22:6		Area			4.3	Control	2.6	31.6	**	%	91.5	64.4	0.6	1.4	**
				Total			3.0	Control	3.2	30.6	**						
790.5	PE 40:6	PE 20:1/20:5		Area			2.5	Control	10.0	25.0	**	%	19.7	8.5	4.8	3.5	**
		PE 18:0/22:6		Area			1.0	GM	4.7	26.8		%	80.3	91.5	1.2	0.3	**
				total			1.1	Control	5.6	26.6							
792.6	PE 40:5	PE 18:0/22:5	PE 20:1/20:4	Area			2.9	GM	6.7	20.6	**	%	81.2	86.5	3.4	2.0	*
		PE 20:0/20:5		Area			2.0	GM	12.5	30.4	*	%	18.8	13.5	14.9	13.1	*
				total			2.7	GM	3.7	21.4	**						
794.6	PE 40:4	PE 20:1/20:3		Area			2.9	Control	20.8	26.4	**	%	76.9	11.3	3.2	10.2	**
		PE 18:0/22:4		Area			6.7	GM	35.2	22.4	**	%	14.8	44.2	15.1	15.0	**
		PE 20:0/20:4		Area			13.3	GM	19.5	52.0	*	%	8.3	44.5	6.5	17.5	**
				Total			2.4	GM	22.3	35.5							
814.5	PE 42:8	PE 20:2/22:6 (a)		Area			1.1	Control	12.4	30.5		%	93.2	82.2	1.6	1.5	**
		PE 20:2/22:6 (b)		Area			2.8	GM	11.5	33.2	*	%	6.8	17.8	21.5	6.9	**
				total			1.1	GM	10.9	30.8		Ratio a/b	14.1	4.6	23.8	8.3	**
818.6	PE 42:6	PE 20:1/22:5		Area			1.1	GM	7.3	21.2		%	24.7	14.7	13.0	10.7	**
		PE 20:0/22:6		Area			2.0	GM	15.8	23.0	*	%	75.3	85.3	4.3	1.8	**
				total			1.8	GM	12.3	22.2	*						
822.5	PC 36:6	PC 18:3/18:3		Area			1.6	GM	15.3	24.0		%	4.3	21.6	15.7	10.3	**
		PC 16:1/20:5		Area			3.3	Control	12.6	24.9	**	%	19.0	17.8	13.5	7.7	
		PC 14:0/22:6		Area			3.8	Control	4.5	33.2	**	%	76.7	60.7	2.8	5.9	**
				Total			3.1	Control	2.1	29.3	**						
826.6	PC 36:4	PC 18:2/18:2		Area			1.6	Control	6.8	21.3	**	%	28.9	15.6	5.0	2.8	**
		PC 18:1/18:3		Area			2.6	Control	22.5	28.3	*	%	43.3	14.5	14.4	9.1	**
		PC 16:0/20:4 (a)		Area			1.2	Control	11.5	7.8		%	11.8	9.1	20.3	17.4	
		PC 16:0/20:4 (c)		Area			1.4	Control	35.9	45.8		%	3.2	1.9	45.6	26.7	
		PC 16:0/20:4 (b)		Area			5.3	GM	9.5	24.0	**	%	12.8	58.9	14.6	1.4	**
				Total			1.1	GM	8.9	22.9		Ratio b/a	1.1	6.6	7.9	17.8	**
828.6	PC 36:3	PC 18:1/18:2		Area			3.4	Control	6.6	18.6	**	%	70.0	33.7	6.3	3.6	**
		PC 16:0/20:3 (a)	PC 18:1/18:2	Area			1.0	GM	15.7	20.1		%	29.4	49.8	15.0	2.2	**
		PC 16:0/20:3 (b)		Area			15.9	GM	33.5	19.0	**	%	0.6	16.5	36.2	1.0	**
				Total			1.6	Control	2.6	19.2	**	Ratio a/b	49.4	3.0	33.0	1.6	**
830.6	PC 36:2	PC 18:1/18:1		Area			6.5	Control	17.8	6.2	**	%	51.4	23.7	9.0	18.7	**
		PC 16:0/20:2		Area			3.0	Control	10.6	29.8	**	%	22.8	21.5	15.0	9.3	
		PC 18:0/18:2		Area			1.3	Control	8.0	26.4		%	25.7	54.9	11.9	5.3	**
				Total			2.9	Control	8.5	22.1	**						
848.5	PC 38:7	PC 18:2/20:5		Area			1.7	GM	8.5	24.6	*	%	53.6	85.2	3.6	2.4	**
		PC 16:1/22:6 (a)		Area			3.9	Control	3.7	34.9	**	%	31.4	7.4	2.7	17.8	**
		PC 16:1/22:6 (b)		Area			1.8	Control	6.7	34.5	*	%	15.1	7.4	10.3	11.7	**
				Total			1.1	GM	4.9	25.4		Ratio a/b	2.1	1.0	10.6	12.2	**
850.6	PC 38:6	PC 18:2/20:4		Area			9.6	GM	13.9	24.2	**	%	1.1	15.7	13.9	1.4	**
		PC 18:1/20:5		Area			2.0	Control	14.9	26.7	*	%	17.3	12.5	11.5	7.2	*
		PC 16:0/22:6		Area			1.7	Control	1.0	23.7	**	%	81.6	71.9	2.5	1.6	**
				Total			1.5	Control	3.2	24.0	*						
852.6	PC 38:5	PC 18:1/20:4 (a)		Area			1.4	Control	1.8	17.1	*	%	7.7	4.1	12.8	21.0	**
		PC 16:0/22:5	PC 18:1/20:4 (b)	Area			1.4	GM	2.9	23.1		%	51.5	51.3	11.0	4.6	
		PC 18:0/20:5		Area			1.5	GM	31.4	29.1		%	40.8	44.7	16.2	5.8	
				Total			1.4	GM	14.4	25.0		Ratio b/a	6.7	12.9	3.5	23.2	*
854.6	PC 38:4	PC 18:2/20:2		Area			2.1	Control	14.1	17.8	**	%	35.4	10.3	7.6	6.2	**
		PC 18:3/20:1	PC 18:1/20:3	Area			1.1	GM	13.0	9.6		%	34.8	22.4	6.2	7.6	**
		PC 16:0/22:4		Area			2.7	GM	28.9	14.5	**	%	11.0	17.8	21.1	1.4	**
		PC 18:0/20:4		Total			4.5	GM	33.0	15.4	**	%	18.8	49.4	37.5	3.8	**
				%			1.7	GM	7.6	13.5	**						
856.6	PC 38:3	PC 18:2/20:1	PC 18:1/20:2	Area			3.1	Control	1.3	19.9	**	%	90.5	65.6	0.7	2.4	**
		PC 18:0/20:3		Area			1.6	GM	8.9	26.6		%	9.5	34.4	6.9	4.6	**
				total			2.2	Control	2.0	22.2	**						

858.6	PC 38:2	PC 18:1/20:1		Area			3.6	Control	19.5	28.9	**	%	82.9	52.8	3.5	13.9	**
		PC 18:0/20:2		Area			1.3	GM	3.2	43.5		%	17.1	47.2	16.8	15.5	**
				total			2.3	Control	15.9	34.6	*						
867.5	PG 44:11	PG 22:5/22:6 (a)		Area			1.7	GM	21.7	42.1		%	97.3	90.7	0.7	2.5	**
		PG 22:5/22:6 (b)		Area			6.6	GM	24.4	56.8		%	2.7	9.3	24.3	24.8	**
				total			1.8	GM	21.4	43.5		Ratio a/b	36.8	10.3	22.0	31.1	**
872.5	PC 40:9	PC 20:4/20:5 (a)		Area			3.7	GM	12.0	20.6	**	%	12.3	26.3	13.5	3.3	**
		PC 18:3/22:6		Area			1.2	GM	5.9	25.1		%	85.0	60.4	2.8	3.2	**
		PC 20:4/20:5 (b)		Area			8.6	GM	57.2	24.1	**	%	2.7	13.3	59.6	13.9	**
				Total			1.7	GM	3.2	23.0	*	Ratio a/b	6.6	2.0	82.6	15.1	
874.6	PC 40:8	PC 18:3/22:5		Area			2.9	GM	14.1	22.3	**	%	4.8	11.2	14.6	2.7	**
		PC 20:3/20:5	PC 20:4/20:4	Area			5.3	GM	35.8	21.8	**	%	7.2	30.7	34.9	2.9	**
		PC 18:2/22:6		Area			1.2	Control	2.1	23.2		%	88.0	58.1	2.1	1.2	**
				Total			1.2	GM	1.7	22.5							
876.6	PC 40:7	PC 18:2/22:5		Area			1.7	GM	3.2	24.3	*	%	8.2	25.4	4.5	10.2	**
		PC 20:2/20:5	C20:3/20:4	Area			2.4	GM	24.4	32.4	*	%	7.4	32.3	30.1	8.4	**
		PC 18:1/22:6		Area			3.7	Control	9.8	23.8	**	%	84.4	42.3	3.0	4.2	**
				Total			1.8	Control	6.7	26.0	**						
880.6	PC 40:5	PC 18:0/22:5		Area			1.7	GM	14.8	16.6	*	%	81.5	70.3	2.1	2.4	**
		PC 20:0/20:5		Area			3.1	GM	25.9	18.8	**	%	18.5	29.7	9.2	5.6	**
				total			2.0	GM	16.9	16.8	*						
881.5	PI 38:6	PI 18:1/20:5		Area			5.5	Control	14.8	27.0	**	%	62.3	54.2	8.4	6.0	
		PI 16:1/22:6		Area			4.0	Control	7.4	21.6	**	%	37.7	45.8	13.9	7.1	
				total			4.8	Control	6.7	24.0	**						
882.6	PC 40:4	PC 20:1/20:3		Area			1.2	Control	17.4	16.4		%	78.3	32.0	4.7	15.3	**
		PC 18:0/22:4		Area			4.6	GM	16.9	22.0	**	%	15.5	34.2	23.4	8.3	**
		PC 20:0/20:4		Area			11.8	GM	12.5	50.1	*	%	6.2	33.8	1.6	22.9	**
				Total			2.1	GM	13.5	28.4	*						
883.5	PI 38:5	PI 18:1/20:4		Area			3.6	Control	3.9	22.5	**	%	45.5	66.6	9.2	18.8	
		PI 18:0/20:5		Area			8.2	Control	15.4	54.8	**	%	54.5	33.4	7.7	37.5	
				total			5.2	Control	7.9	23.6	**						
886.6	PC 40:2	PC 20:1/20:1		Area			N/A	Control	8.2	0.0	**	%	32.6	0.0	14.7	0.0	**
		PC 18:1/22:1		Area			N/A	Control	15.8	0.0	**	%	67.4	0.0	7.1	0.0	**
				total			N/A	Control	9.6	0.0	**						
898.6	PC 42:10	PC 20:5/22:5		Area			5.0	GM	31.2	25.7	**	%	21.3	32.1	26.6	11.7	
		PC 20:4/22:6 (a)		Area			2.0	GM	20.1	23.4	*	%	61.7	37.3	12.6	9.1	**
		PC 20:4/22:6 (b)		Area			5.9	GM	14.4	13.0	**	%	17.1	30.6	16.6	7.2	**
				Total			3.3	GM	13.6	19.2	**	Ratio a/b	3.7	1.2	28.6	11.9	*
900.6	PC 42:9	PC 20:5/22:4		Area			21.2	GM	38.1	18.7	**	%	3.3	29.0	30.7	3.4	**
		PC 20:3/22:6		Area			1.8	GM	10.0	19.6	*	%	96.7	71.0	1.0	1.4	**
				total			2.4	GM	10.9	19.2	**						**
906.6	PC 42:6	PC 20:0/22:6 (a)		Area			1.1	GM	2.1	19.4		%	19.0	17.0	18.2	7.4	
		PC 20:1/22:5		Area			1.5	Control	30.4	26.0		%	42.7	23.2	11.8	18.7	**
		PC 20:0/22:6 (b)		Area			1.9	GM	14.4	32.5		%	38.3	59.8	4.9	8.5	**
				Total			1.2	GM	18.1	26.2		Ratio b/a	2.1	3.5	16.5	15.2	*
913.6	PI 40:4	PI 18:0/22:4		Area			2.2	GM	40.9	19.0	*	%	50.0	34.1	20.0	24.4	
		PI 20:0/20:4		Area			4.5	GM	9.1	18.5	**	%	50.0	65.9	19.9	12.6	
				total			3.3	GM	20.7	8.3	**						
926.6	PC 44:10	PC 22:5/22:5		Area			5.3	GM	23.1	16.0	**	%	67.8	47.8	14.8	5.0	*
		PC 22:4/22:6		Area			12.2	GM	38.8	22.1	**	%	32.2	52.2	31.1	4.5	*
				total			7.6	GM	22.5	18.9	**						
